# Phenoxazine Derivative ST61 Displays an Oxidative-Stress-Mediated Cytotoxic Activity Against Human Cancer Cells

**DOI:** 10.3390/biom16050689

**Published:** 2026-05-06

**Authors:** Adamantia Papadopoulou, Socratis Avgeris, Savvas Thysiadis, Christos Siokatas, Stelios Astaras, Faye Soukou, Eleni Mavrogonatou, Harris Pratsinis, Gerassimos E. Voutsinas, Vasiliki Sarli, Dimitris Kletsas

**Affiliations:** 1 Laboratory of Cell Proliferation and Ageing, Institute of Biosciences and Applications, National Centre for Scientific Research “Demokritos”, 15341 Athens, Greece; apapad@bio.demokritos.gr (A.P.); steliosastaras@gmail.com (S.A.); soukouf@bio.demokritos.gr (F.S.); elmavro@bio.demokritos.gr (E.M.); hprats@bio.demokritos.gr (H.P.); 2 Laboratory of Molecular Carcinogenesis and Rare Disease Genetics, Institute of Biosciences and Applications, National Centre for Scientific Research “Demokritos”, 15341 Athens, Greece; savgeris@bio.demokritos.gr (S.A.); mvoutsin@bio.demokritos.gr (G.E.V.); 3 Organic Chemistry Laboratory, School of Chemistry, Faculty of Sciences, Aristotle University of Thessaloniki, 54124 Thessaloniki, Greece; sthisiadis@hotmail.com (S.T.); siokatas@chem.auth.gr (C.S.); sarli@chem.auth.gr (V.S.)

**Keywords:** phenoxazines, anti-cancer activity, oxidative stress, ROS generation, HSP27, MCF7 breast cancer cells

## Abstract

Ninety-five structurally diverse chemically synthesized compounds, retrieved from the chemical library of the national research infrastructure OPENSCREEN-GR, were subjected here to a structure-agnostic bioactivity-driven screen focused on putative anti-cancer activities using a number of human cell lines derived from diverse types of cancer. Interestingly, the top four compounds that displayed a broad anti-cancer activity (with no apparent photosensitizing activity) during unbiased biological evaluation were then identified as phenoxazine derivatives. In addition to their cytotoxic activity, phenoxazine derivatives BS115 and ST61 were also found to be cytostatic by inducing a p53-independent G2/M cell cycle arrest as well as a G0/G1 arrest only in cells harboring a functional p53. A kinetics analysis using two multiplex immunoassays and siRNA-mediated knockdown of HSP27 revealed that the latter protein is a key molecule in the response of cancer cells to ST61 via its phosphorylation by p38 MAPK. The main mechanism underlying ST61’s anti-cancer activity was found to involve oxidative stress, as scavenging of ST61-induced reactive oxygen species by N-acetyl-cysteine led to the abrogation of the compound’s cytotoxic effect.

## 1. Introduction

Despite higher percentages in timely diagnosis and more accurate prognosis for a number of cancers, as well as early intervention and improved treatment, cancer is still the major cause of mortality worldwide [1]. Accordingly, the scientific interest in the search for new compounds offering high tumor selectivity with minimal side-effects on normal tissues is ever growing. Bioactive compounds originate from two principal sources. On the one hand, naturally occurring compounds exhibit either specific or broad therapeutic potential and have therefore been widely used in the treatment of human diseases [2]. However, given that natural products are complex mixtures containing a great number of chemical constituents that may act synergistically or competitively, identification of the active components that may be responsible for their efficacy is often challenging. On the other hand, rationally designed synthetic molecules represent another important source of bioactive compounds [3]. These compounds offer the advantage of being chemically synthesized under controlled conditions, with well-defined chemical structures often derived from previously identified scaffolds with targeted biological activities, and they may be inspired by and based on structures of natural-product-isolated compounds [4,5]. Furthermore, biological evaluation of synthesized molecules may reveal novel structural features and provide insight into additional or alternative functions leading to drug repurposing [6], an approach facilitated by technologies of high-throughput screening [7]. An important class of synthesized heterocycles, i.e., phenoxazine derivatives, have attracted attention due to a wide range of biological properties, including anti-bacterial, anti-viral, anti-tumor, antioxidant, and anti-inflammatory activities [8,9,10,11,12,13,14].

**Figure 1:**
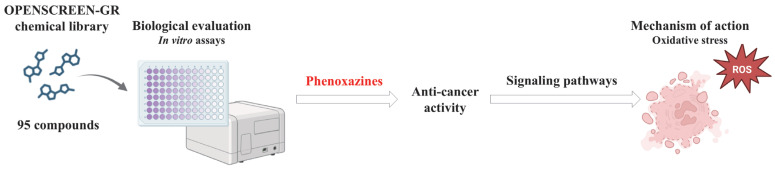
Graphical representation of the workflow followed in the current study for the bioactivity-guided screening of 95 compounds derived from the OPENSCREEN-GR chemical library, focusing on their putative anti-cancer properties as well as the elucidation of their mechanism of action.

The goal of the current work was the bioactivity-driven screening of a series of well-defined compounds from the structurally diverse chemical library of OPENSCREEN-GR, the latter being a national research infrastructure aiming at evaluating the biological activity of compounds of natural and synthetic origin (https://openscreen.bio.demokritos.gr/). Ninety-five chemically synthesized compounds were screened here following a structure-agnostic workflow for putative anti-cancer activities against several human cancer cell lines, such as epidermoid squamous carcinoma, non-small-cell lung cancer, breast cancer, fibrosarcoma, bladder cancer and metastatic melanoma cells. Interestingly, the hit compounds emerging from the blind and unbiased biological evaluation to display a broad anti-cancer activity shared the phenoxazine core. Further investigation into the mechanism of action of the most promising compound revealed that its anti-cancer activity is mediated by the selective increase in intracellular reactive oxygen species (ROS) in cancer cells (Figure 1).

## 2. Materials and Methods

### 2.1. Synthesis and Structural Characterization of Phenoxazine Derivatives

**Scheme 1:**
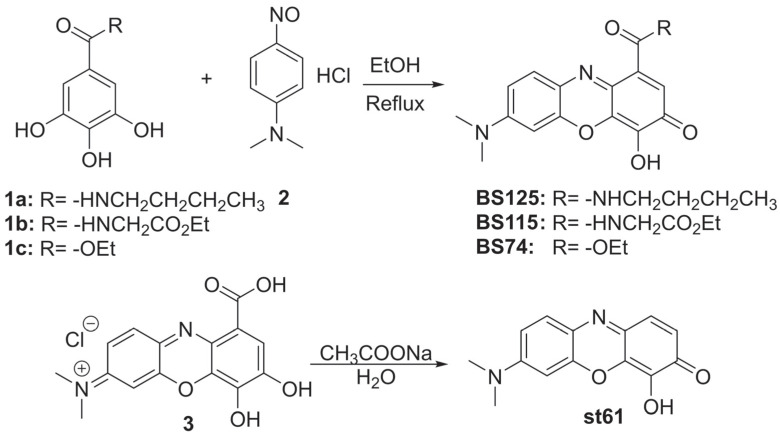
Synthesis of phenoxazine derivatives.

Phenoxazine derivatives were synthesized according to Scheme 1. The synthesis of phenoxazine ST61 was performed as previously reported by our group and others [15] using the decarboxylation of gallocyanine in the presence of CH_3_COONa in water and with heating. The other phenoxazines were synthesized using the classical method starting from the reaction of gallamide or gallate derivatives and N,N-dimethyl-4-nitrosoaniline hydrochloride under reflux in EtOH.

All reactions were conducted under an argon atmosphere using commercial reagents without additional purification. The progress of all reactions was monitored by TLC, visualized under UV light, and developed with aqueous ceric sulfate/phosphomolybdic acid, ethanolic p-anisaldehyde solution, and heat. ^1^H and ^13^C NMR spectra were recorded at 500 MHz and 126 MHz, respectively, on an Agilent DD2 spectrometer (500 MHz, Varian, Palo Alto, CA, USA) with tetramethylsilane (TMS) as an internal standard. Chemical shifts are reported in δ (ppm) from internal reference peaks (TMS ^1^H 0.00; CDCl_3_ ^1^H 7.26, ^13^C 77.16, (CD_3_)_2_SO ^1^H 2.50, ^13^C 39.52, pyridine-*d*5 ^1^H 8.74, 7.58, 7.22; ^13^C 150.35, 135.91, 123.87). LC-MS analysis was performed on a Shimadzu LC-20AD system connected to a Shimadzu LCMS-2010EV (Shimadzu, Kyoto, Japan) equipped with a C_18_ analytical column (Supelco Discovery C18, 5 μm, 250 × 4.6 mm).

N-butyl-7-(dimethylamino)-4-hydroxy-3-oxo-3*H*-phenoxazine-1-carboxamide (BS125): N-butyl-3,4,5-trihydroxybenzamide 1a (67 mg, 0.298 mmol) and freshly prepared N,N-dimethyl-4-nitrosoaniline hydrochloride 2 (67 mg, 0.358 mmol) were added to 1 mL of ethanol. The reaction mixture was refluxed for 1 h, during which the solution turned into a purple color. Then, the reaction mixture was cooled, and the crystals were collected by suction filtration on a filter paper and washed with chilled EtOH. The solid was dried under reduced pressure to give 55 mg of BS125 as a dark purple solid (52% yield). BS125: ^1^H NMR (500 MHz, pyridine-*d*5) δ 9.88 (s, 1H), 8.24 (s, 1H), 7.64 (d, *J* = 9.0 Hz, 1H), 6.70 (dd, *J* = 9.0, 2.7 Hz, 1H), 6.55 (d, *J* = 2.6 Hz, 1H), 3.66 (dd, *J* = 12.7, 6.8 Hz, 2H), 2.91 (s, 6H), 1.75–1.61 (m, 2H), 1.47 (dd, *J* = 15.0, 7.4 Hz, 2H), 0.94 (t, *J* = 7.4 Hz, 3H); ^13^C NMR (126 MHz, pyridine-*d*5) δ 179.7, 164.2, 154.8, 147.6, 141.4, 137.9, 133.2, 133.0, 132.6, 131.9, 125.5, 111.3, 97.4, 40.5 (2C), 40.3, 32.3, 21.1, 14.4 (Supplementary Figure S1); FT-IR (KBr): 3515, 3460, 2948, 2860, 1640, 1598, 1547, 1482,1420, 1380, 1301, 1265, 1195, 1132, 1028; ESI-MS analysis, positive mode: *m*/*z* calcd mass for C_19_H_22_N_3_O_4_^+^ [M + H]^+^ = 356.16 was found 356 (Supplementary Figure S2).

Methyl 2-(7-(dimethylamino)-4-hydroxy-3-oxo-3*H*-phenoxazine-1-carboxamido)acetate (BS115): ethyl 2-(3,4,5-trihydroxybenzamido)acetate 1b (70 mg, 0.275 mmol) and freshly prepared N,N-dimethyl-4-nitrosoaniline hydrochloride 2 (62 mg, 0.33 mmol) were added to 1 mL of ethanol. The reaction mixture was refluxed for 1 h, during which the solution turned into a purple color. Then, the reaction mixture was cooled, and the crystals were collected by filtration on a filter paper and washed with chilled EtOH. The solid was dried under reduced pressure to give 50 mg of BS115 as a dark purple solid (59% yield). BS115: ^1^H NMR (500 MHz, pyridine-*d*5) δ 10.69 (s, 1H), 8.24 (s, 1H), 7.75 (d, *J* = 8.8 Hz, 1H), 6.66 (d, *J* = 8.8 Hz, 1H), 6.53 (s, 1H), 4.57 (d, *J* = 5.0 Hz, 2H), 4.25 (q, *J* = 7.1 Hz, 2H), 2.92 (s, 6H), 1.20 (t, *J* = 7.1 Hz, 3H); ^13^C NMR (126 MHz, pyridine) δ 179.3, 170.6, 164.2, 154.7, 147.5, 140.9, 137.8, 133.3, 133.1, 131.9, 131.1, 125.4, 111.1, 97.2, 61.6, 43.0, 40.3 (2C), 14.5 (Supplementary Figure S3); FT-IR (KBr): 3437, 3293, 2977, 1741, 1655, 1599, 1554, 1523, 1488, 1406, 1382, 1300, 1259, 1203, 1138, 1088, 1042, 1014; ESI-MS, positive mode: *m*/*z* calcd mass for C_19_H_20_N_3_O_6_^+^ [M + H]^+^ = 386.14 was found 386 (Supplementary Figure S4).

Ethyl 7-(dimethylamino)-4-hydroxy-3-oxo-3H-phenoxazine-1-carboxylate (BS74, Supplementary Figure S5): BS74 was synthesized following a previously described synthetic method reported by our group [16]. Ethyl gallate 1c (200 mg, 1.01 mmol) and freshly prepared N,N-dimethyl-4-nitrosoaniline hydrochloride 2 (225 mg, 1.21 mmol) were charged into a round flask and dissolved in 3.5 mL of ethanol. The reaction mixture was refluxed for 1 h. Then, it was cooled, and the crystals were collected with filtration. The solid was washed with chilled ethanol followed by recrystallization from ethanol (35% yield). The spectroscopic data agree with the literature values. BS74: ^1^H NMR (500 MHz, pyridine-*d*5) δ 7.68 (d, *J* = 9.1 Hz, 1H), 7.44 (s, 1H), 6.61 (dd, *J* = 9.1, 2.5 Hz, 1H), 6.50 (d, *J* = 2.5 Hz, 1H), 4.45 (q, *J* = 7.1 Hz, 2H), 2.84 (s, 6H), 1.30 (t, *J* = 7.1 Hz, 3H).

7-(Dimethylamino)-4-hydroxy-3*H*-phenoxazin-3-one (ST61, Supplementary Figure S6): ST61 was synthesized according to a previously described method from the decarboxylation of gallocyanine hydrochloride [15]. Sodium acetate (1 g, 12.19 mmol) was added to a solution of gallocyanine hydrochloride 3 (1 g, 2.97 mmol) in water (100 mL). The reaction mixture was refluxed for 5 h and then cooled at 0 °C. The reaction mixture was allowed to cool at 0 °C and neutralized with a saturated solution of sodium carbonate. The crystals formed were collected by filtration. To improve purity, the solid was refluxed in ethanol (9 mL) for 10 min and stored at 8 °C overnight. The product was then collected by filtration as a dark blue solid (650 mg, yield 85%). The spectroscopic data agree with the literature values. ST61: ^1^H NMR (500 MHz, DMSO-*d*6) δ 7.49 (d, *J* = 9.2 Hz, 1H), 7.24 (d, *J* = 8.5 Hz, 1H), 6.84–6.68 (m, 2H), 6.60 (s, 1H), 3.11 (s, 6H).

### 2.2. Cells and Cell Culture Conditions

Cell lines used in the current study were as follows: A-431 epidermoid carcinoma cells, derived from an 85-year-old female; A549 cells, isolated from the lung tissue of a 58-year-old male with lung cancer; breast adenocarcinoma MCF7 cells, isolated from a 69-year-old female, and MDA-MB-231 cells, isolated from a 51-year-old female; HT-1080 epithelial cells, derived from the connective tissue of a 35-year-old male fibrosarcoma patient; T24 urinary bladder transitional carcinoma cells, isolated from an 81-year-old female (all obtained from the American Tissue Culture Collection ATCC, Rockville, MD, USA, and retrieved from the pre-existing cell bank of the Laboratory of Cell Proliferation and Ageing, NCSR “Demokritos”); and highly metastatic melanoma WM-266-4 cells, derived from a 58-year-old female’s skin metastasis with stage III melanoma (purchased from the European Collection of Authenticated Cell Cultures ECACC/Sigma-Aldrich, Munich, Germany, and retrieved from the cell bank of the Laboratory of Molecular Carcinogenesis and Rare Disease Genetics, NCSR “Demokritos”). Primary cultures of normal human fibroblasts established after dissection and collagenase digestion of normal tissues from the breast stroma of consenting female volunteers undergoing surgery that were used in the current study were also from the pre-existing cell bank of the Laboratory of Cell Proliferation and Ageing [17,18].

All cells were cultured in Dulbecco’s Modified Eagle Medium (DMEM, Capricorn Scientific GmbH, Ebsdorfergrund, Germany) supplemented with penicillin (100 U/mL)/streptomycin (100 mg/mL) (obtained from Biowest, Nuaillé, France) and 10% (*v*/*v*) fetal bovine serum (FBS, from Gibco BRL, Invitrogen, Paisley, UK) in a humidified atmosphere of 5% CO_2_ at 37 °C, were subcultured when necessary using a trypsin/citrate (0.25%:0.30% *w*/*v*) solution and were regularly tested to ensure their mycoplasma-free status.

### 2.3. Cell Viability Assays

The MTT cell viability assay was performed as previously reported [19]. In brief, cells were cultured in 96-well plates until they became sub-confluent. After the addition of the compounds under investigation at the selected concentrations, cells were incubated for 24 or 72 h. Cells treated with the diluent (dimethyl sulfoxide, DMSO) at the respective concentrations served as the control samples. At the end of the incubation period, the culture medium was aspirated and replaced with 1 mg/mL 3-(4,5-dimethylthiazol-2-yl)-2,5-diphenyltetrazolium bromide (MTT, Sigma, St. Louis, MO, USA) in phenol-red-free DMEM (PAN Biotech GmbH, Aidenbach, Germany), after which the cells were further incubated for 4 h. The resulting formazan crystals were dissolved in isopropanol, the optical density at 550 nm was measured using a Spark microplate analyzer (Tecan, Männedorf, Switzerland) and cell viability was estimated as the percentage of the respective control samples.

The neutral red uptake assay was performed as reported before [20,21]. In brief, after treatment, culture medium was aspirated and replaced by a neutral red solution (20 μg/mL) in phenol-red-free DMEM, and cells were further incubated for 4 h before a wash with phosphate-buffered saline (PBS). The dye was extracted using an acidified ethanol solution. Fluorescence was measured by a Tecan Spark microplate reader at excitation and emission wavelengths of 530 and 645 nm, respectively. Cell viability was expressed as a % ratio of untreated cells. Each MTT and neutral red experiment was performed in triplicate. All experiments were conducted in the dark to avoid photosensitization of the compounds.

### 2.4. Cell Cycle Analysis by Flow Cytometry

For cell cycle analysis, cells treated with the selected concentrations of the compounds for 24 and 48 h were harvested by trypsinization, washed once with PBS, fixed with 50% (*v*/*v*) ice-cold ethanol and stored at 4 °C. Staining was performed with a solution containing 50 μg/mL propidium iodide (PI) (Sigma), 5 mM MgCl_2_ and 10 μg/mL RNase A (Sigma). Flow-cytometric analysis was conducted using a FACSCalibur flow cytometer and the Cell Quest software v. 3.1 (Becton Dickinson, San Jose, CA, USA), while cell cycle distribution was quantified using the Modfit software v. 2.0 (Verity Software House, Topsham, ME, USA). Each experiment was performed in triplicate.

### 2.5. Multiplex Immunoassays

Phosphorylation levels of ATF-2 (Thr71), ERK1/2 (Thr202/Tyr204, Thr185/Tyr187), HSP27 (Ser78), SAPK/JNK (Thr183/Tyr185), MEK1 (Ser217/Ser221), p38 MAPK (Thr180/Tyr182), p53 (Ser15), p90 RSK (Ser380) and STAT3 (Ser727) in MCF7 cells were estimated using the 9-Plex Bio-Plex Pro™ Cell Signaling MAPK Panel (Bio-Rad Laboratories Inc., Hercules, CA, USA), while expression and phosphorylation levels of ATR, MDM2 and p21 and of Chk1 (Ser345), Chk2 (Thr68), H2A.X (Ser139) and p53 (Ser15), respectively, were detected using the 7-Plex MILLIPLEX^®^ DNA Damage/Genotoxicity Magnetic Bead kit (Merck KGaA, Darmstadt, Germany), according to the manufacturers’ instructions, as previously reported [22]. In brief, cell lysates of MCF7 cells were prepared, the total protein concentration was measured using the Bradford reagent (Bio-Rad Laboratories) and samples were stored at −80 °C. For the Luminex analysis, samples as well as positive and negative cell lysate controls provided by the kits were diluted in assay buffer to the appropriate concentration. Data was acquired by the Luminex Bio-Plex MAGPIX^®^ Multiplex Reader using the xPONENT software v. 4.2 (Luminex Corporation, Austin, TX, USA). Results are presented as the ratio of the net median fluorescence intensity (MFI) of treated samples at the selected time points to the net MFI of the untreated control (t = 0). Each experiment was performed in triplicate.

### 2.6. Protein Extraction and Western Blot Analysis

Total protein extraction was performed as reported previously using a Laemmli sample buffer containing protease and phosphatase inhibitors (Sigma) [23]. Separated proteins in SDS polyacrylamide gels that were transferred to polyvinylidene fluoride (PVDF) membranes were subjected to Western blot analysis using antibodies raised against phospho-p38 MAPK (Thr180/Tyr182), p38 MAPK, phospho-SAPK/JNK (Thr183/Tyr185), SAPK/JNK, phospho-p53 (Ser15), phospho-Chk1 (Ser345), phospho-Chk2 (Thr68), phospho-HSP27 (Ser78), phospho-Akt (Ser473) (all purchased from Cell Signaling Technology, Hertfordshire, UK), phospho-ERK1/2 (Thr202/Tyr204), ERK2 (both supplied by BD Transduction Laboratories, Bedford, MA, USA) and glyceraldehyde 3-phosphate dehydrogenase (GAPDH, provided by Santa Cruz Biotechnology, Santa Cruz, CA, USA). Secondary horseradish-peroxidase-conjugated antibodies were obtained from Sigma. Immune complexes were visualized using an ECL reagent (Merck). Densitometric analysis was conducted with ImageJ v.1.54 (NIH, Bethesda, MD, USA).

### 2.7. Transfection Experiments

Inhibition of HSP27 expression in MCF7 cells was performed by using the commercially available antisense oligonucleotide OGX-427 (MedChemExpress, South Brunswick Township, NJ, USA), designed to bind to HSP27 mRNA. Transfection experiments were performed as described in the past [20,24] with slight modifications. Briefly, MCF7 cells were transfected with 30 nM of either a predesigned scramble (5′-UAAUGUAUUGGAACGCAUATT-3′, Eurofins Genomics, Ebersberg, Germany) or OGX-427 in serum-free OptiMEM I medium using lipofectamine 2000 (Invitrogen). After a 5 h incubation in a humidified atmosphere of 5% CO_2_ at 37 °C, the transfection medium was replaced by culture medium supplemented with 10% (*v*/*v*) FBS, and cells were further incubated for 72 h before treatment with the compound for another 24 h. Cell viability was expressed as the % ratio of cells treated with scramble in the absence of the compound. Each experiment was performed in sextuplicate.

### 2.8. Estimation of Intracellular Levels of Reactive Oxygen Species (ROS)

Intracellular ROS levels were measured using a 2′,7′-dichlorfluorescein-diacetate (DCFH-DA) assay, as described before [24]. Cells were cultured in clear-bottomed black 96-well microplates (Porvair Ltd., Fareham, UK) in DMEM supplemented with 10% (*v*/*v*) FBS. When confluent, cells were pre-incubated with 10 μM of freshly diluted in serum-free DMEM DCFH-DA (Sigma) for 1 h at 37 °C and then exposed to the compound at concentrations ranging from 0 to 25 μΜ and further incubated at 37 °C. Experiments were conducted in the dark to avoid photosensitization of the compound. Fluorescence was recorded at various time points (0–72 h) using a fluorescent microplate reader (Fluostar, Optima BMG LABTECH excitation wavelength: 485 nm, emission wavelength: 520 nm). ROS production was expressed as the % ratio of the untreated control. Each experiment was conducted in triplicate.

### 2.9. RNA Extraction and Reverse Transcription Quantitative Polymerase Chain Reaction (RT-qPCR)

Total RNA was extracted using the Trizol reagent (Invitrogen) and quantified using a Nanodrop ND-1000 spectrophotometer (Nanodrop Technologies, Wilmington, DE, USA) before first-strand cDNA synthesis with a PrimeScript RT Reagent Kit (Takara, Tokyo, Japan) according to the manufacturer’s instructions, as reported before [20,24]. qPCR reactions were performed in an AriaMx Real-time PCR System (Agilent Technologies, Santa Clara, CA, USA) with cDNA at a final dilution of 1:100 using a KAPA SYBR FAST qPCR kit (KAPA Biosystems, Boston, MA, USA), and data were collected and processed with the Agilent Aria software v. 2.1. GAPDH served as the reference gene, and mRNA levels were quantified using the 2^−ΔΔCt^ method [25]. Primer sequences used in this study were as follows: HO-1 forward: 5′-GCC-CTT-CAG-CAT-CCT-CAG-TTC-C-3′, HO-1 reverse: 5′-AGT-GGT-CATGGC-CGT-GTC-AAC-3′), GAPDH forward: 5′-GAG-TCC-ACT-GGC-GTC-TTC-3′, GAPDH reverse: 5′-GCA-TTG-CTG-ATG-ATC-TTG-AGG-3′). Each experiment was conducted in duplicate.

## 3. Results

### 3.1. Screening of Compounds “A” and “B” for Anti-Cancer Activity

Ninety-five structurally diverse compounds from the chemical library of OPENSCREEN-GR (**1A**–**48A** and **1B**–**47B**) were initially screened regarding their cytotoxic activity against seven cancer cell lines of different origins/types of cancer. Cancer cell lines tested were human epidermoid squamous carcinoma A-431 cells, human non-small-cell lung cancer A549 cells, human breast cancer MCF7 and MDA-MB-231 cells, human fibrosarcoma HT-1080 cells, human bladder cancer T24 cells and metastatic human melanoma WM-266-4 cells (Supplementary Figures S7–S13).

**Figure 2:**
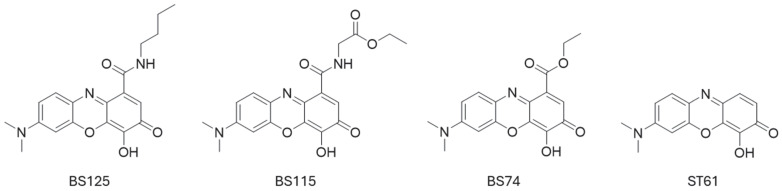
Chemical structure of the four compounds with the most potent anti-cancer activity.

As summarized in Table 1 and Table 2, >10 compounds showed a broad anti-cancer activity (i.e., they were toxic for ≥5 of the tested cancer cell lines). The chemical structure of selected active compounds is provided in Supplementary Table S1 [26,27,28]. Compounds **37A**, **42A**, **11B** and **12B**, in particular, corresponding to the chemical structures BS125, BS115, BS74 and ST61 (Figure 2), were found to exhibit a substantial cytotoxic effect on all or the majority of the cancer cell lines investigated in the current study, with the most potent amongst them being compounds BS115 (**42A**) and ST61 (**12B**). Notably, these four most active compounds possess a common phenoxazine scaffold.

### 3.2. Effect of Compounds ST61 and BS115 on the Cell Cycle Progression of Cancer Cells

**Figure 3:**
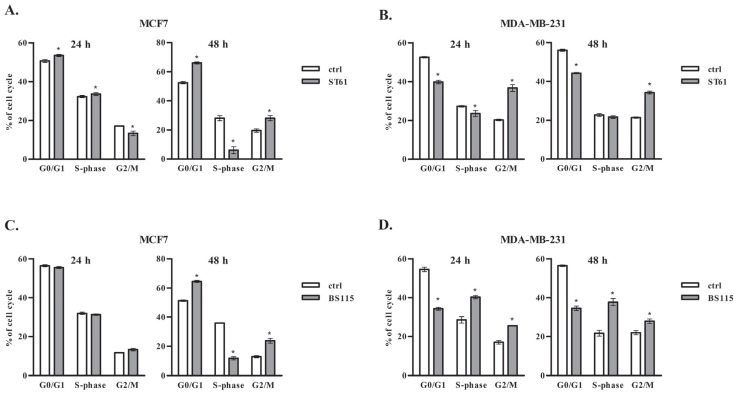
Cell cycle distribution of MCF7 and MDA-MB-231 cancer cells treated with compounds ST61 (**A**,**B**) and BS115 (**C**,**D**). Cells were incubated with the compounds for 24 and 48 h, fixed, stained with propidium iodide and analyzed by flow cytometry. Data presented are mean values ± standard deviations of a representative experiment (*n* = 4, conducted in triplicate). Asterisks represent statistically significant differences in comparison to the respective control (ctrl) for *p* < 0.05 (Student’s *t*-test).

The most potent compounds ST61 and BS115 were then assessed regarding their effect on the cell cycle progression of MCF7 and MDA-MB-231 breast cancer cells. The selection of cell lines was based on their p53 status (MCF7 cells possess a wild-type functional p53, while MDA-MB-231 cells bear a mutant R280K p53), given that the onco-suppressive protein p53 is a main regulator of cell cycle/proliferation. Consistent with the expression of a functional p53 in MCF7 cells, compounds ST61 and BS115 were found to induce activation of both the G1 and G2 checkpoints of the cell cycle (Figure 3A,C). In contrast, compounds ST61 and BS115 induced only a G2/M and an S-phase and G2/M cell cycle arrest, respectively, in MDA-MB-231 cells harboring a mutant p53 (Figure 3B,D). The requirement of a functional p53 for the ST61- and BS115-induced activation of the G1 checkpoint in cancer cells was confirmed by the activation in T24 cells (also bearing a mutant p53, with an in-frame deletion of Y126) of only a G2/M and not a G1 arrest (Supplementary Figure S14).

**Table 2:** Summary of the cytotoxic activity of compounds **1B**–**47B** at 10 μM in A-431, A549, MCF7, MDA-MB-231, HT-1080, T24 and WM-266-4 cancer cells treated with the compounds for 72 h.

	Cell Line	A-431	A549	MCF7	MDA-MB-231	HT-1080	T24	WM-266-4			
Compound											
**1B**											
**2B**											
**3B**											
**4B**											**viability (%)**
**5B**											**<20**
**6B**											**21–40**
**7B**											**41–60**
**8B**											**61–80**
**9B**											**>80**
**10B**											
**11B**											
**12B**											
**13B**											
**14B**											
**15B**											
**16B**											
**17B**											
**18B**											
**19B**											
**20B**											
**21B**											
**22B**											
**23B**											
**24B**											
**25B**											
**26B**											
**27B**											
**28B**											
**29B**											
**30B**											
**31B**											
**32B**											
**33B**											
**34B**											
**35B**											
**36B**											
**37B**											
**38B**											
**39B**											
**40B**											
**41B**											
**42B**											
**43B**											
**44B**											
**45B**											
**46B**											
**47B**											

### 3.3. Mode of Action of Compound ST61 in MCF7 Breast Cancer Cells

**Figure 4:**
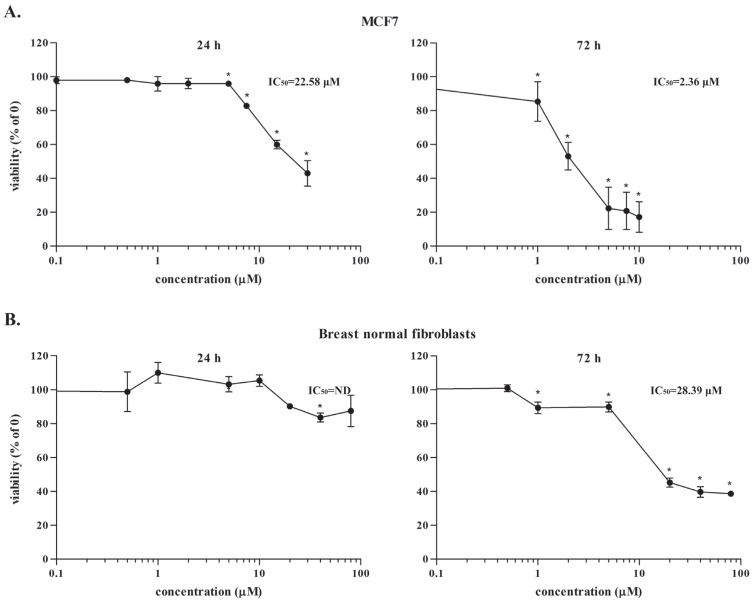
Cytotoxic activity of compound ST61 in MCF7 breast cancer cells (**A**) and normal breast fibroblasts (**B**) for 24 and 72 h. Cells were incubated with concentrations of the compound ranging from 0 to 100 μΜ for 24 or 72 h before aspiration of the culture medium and incubation with 1 mg/mL MTT solution for another 4 h at 37 °C. Optical density of the solubilized formazan crystals (formed only in living cells) was measured at 550 nm, and cell viability was calculated as the percentage ratio of the control sample. Data presented are mean values ± standard deviations (*n* = 3). Asterisks denote statistically significant differences in comparison to 0 μΜ (Student’s *t*-test, *p* < 0.05).

Given the similar effect of ST61 and BS115 on cell cycle progression in cancer cells, the mechanism of action of the most potent compound between the two was further investigated. While cytotoxic for breast cancer cells, compound ST61 was slightly cytotoxic or much less cytotoxic for normal breast fibroblasts after a 24 h or a 72 h incubation, respectively (Figure 4). More specifically, while ST61’s IC_50_ at 24 h was ~25 μM in MCF7 cells, the compound was not cytotoxic for normal breast fibroblasts, even at the highest concentration tested (i.e., 100 μΜ). Furthermore, ST61’s IC_50_ at 72 h was found to be >10-fold higher in normal breast fibroblasts than in MCF7 cells (~28 and ~2 μM in normal breast fibroblasts and MCF7 cells, respectively), demonstrating a pronounced selective activity against cancer cells, a key requirement for a promising anti-cancer agent.

**Figure 5:**
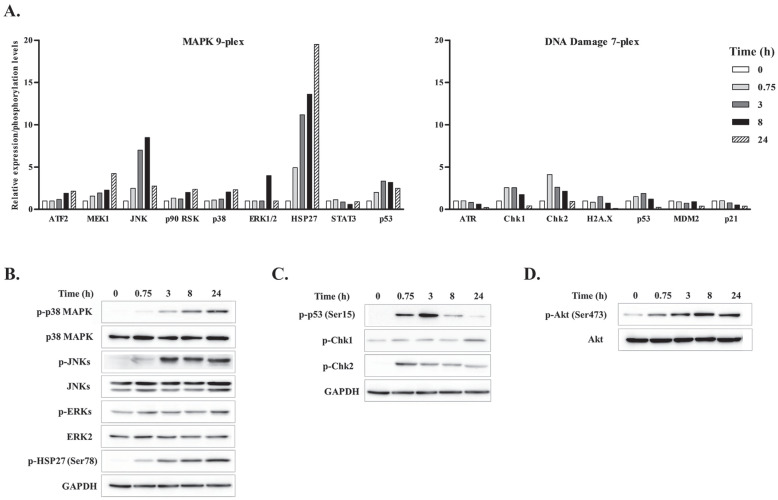
Relative phosphorylation levels of MAPKs, their upstream regulators and downstream targets, and relative expression and phosphorylation levels of proteins involved in the DNA damage response of ST61-treated MCF7 cells. (**A**) MCF7 cells were incubated with ST61 at its 24 h IC_50_ for 0, 0.75, 3, 8 and 24 h before sample collection and Luminex assay. Data presented in the graphs are the ratios of the net MFI of the treated cells to that of the untreated controls. Changes were assessed in phosphorylated ATF-2 (Thr71), MEK1 (Ser217/Ser221), SAPK/JNK (p-JNKs, Thr183/Tyr185), p90 RSK (Ser380), p38 MAPK (p-p38 MAPK, Thr180/Tyr182), ERK1/2 (p-ERKs, Thr202/Tyr204, Thr185/Tyr187), HSP27 (Ser78), STAT3 (Ser727), p53 (p-p53, Ser15), Chk1 (p-Chk1, Ser345), Chk2 (p-Chk2, Thr68) and H2A.X (Ser139) as well as in the total protein levels of ATR, MDM2 and p21. (**B**) Validation by Western blot analysis of the ST61-induced phosphorylation pattern for the three members of the MAPK superfamily (p38 MAPK, ERK1/2 and SAPK/JNK) as well as for the most highly phosphorylated HSP27. (**C**) Validation by Western blot analysis of the ST61-induced phosphorylation pattern for the most highly phosphorylated DNA damage response proteins, p53, Chk1 and Chk2. (**D**) Western blot analysis of Akt phosphorylation at Ser473 on Luminex samples from ST61-treated MCF7 cells. Representative blots (*n* = 3) are presented here. The non-phosphorylated forms of kinases and glyceraldehyde 3-phosphate dehydrogenase (GAPDH) served as loading controls. Original images can be found in Supplementary Materials.

Subsequently, a kinetics analysis using two multiplex immunoassays was conducted to elucidate the effect of ST61 at its 24 h IC_50_ on various protein targets in MCF7 cells at several time points from 0 to 24 h (Figure 5A). With the exception of STAT3, all target proteins included in the MAPK 9-plex were found to become phosphorylated by ST61 in a time-dependent manner. SAPK/JNK and HSP27 displayed the highest ST61-induced phosphorylation. In addition, Chk1, Chk2 and p53—which belong to the DNA damage response pathway—were also found to be phosphorylated by ST61, indicating a genotoxic effect of the compound on MCF7 breast cancer cells (bearing a wild-type functional p53, as mentioned above). ST61-induced phosphorylation was further assessed by Western blot analysis for the three members of the MAPK superfamily (p38 MAPK, ERK1/2 and SAPK/JNK) and the most highly induced protein, i.e., HSP27, as well as for the DNA damage kinases Chk1 and Chk2 and for p53. Western blot analysis validated the multiplex immunoassays’ results in most cases, since it confirmed ST61-mediated enhanced phosphorylation for p38 MAPK, SAPK/JNK, HSP27, Chk2 and p53 (Figure 5B,C and Figure S15). In addition to the multiplex immunoassays’ targets, given the cytotoxic effect of ST61 on MCF7 cells, the phosphorylation status of the known survival protein Akt on Ser473 was also explored by Western blot analysis. Indeed, Akt was found to be phosphorylated in ST61-treated MCF7 cells in a time-dependent manner (Figure 5D and Figure S15).

### 3.4. HSP27 Is a Key Molecule in the Response of MCF7 Breast Cancer Cells to ST61, and Its Phosphorylation Is Mediated by p38 MAPK

**Figure 6:**
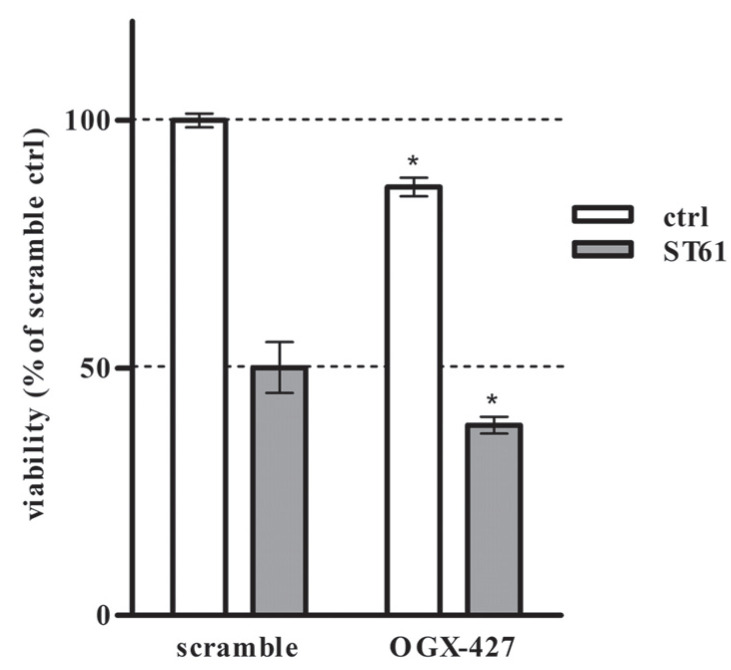
Effect of the inhibition of HSP27 expression on ST61-treated MCF7 cell viability. MCF7 cells were transfected with 30 nM of either a predesigned scramble or OGX-427. Cells were further incubated for 72 h before ST61 addition at its 24 h IC_50_ concentration (25 μΜ) for another 24 h, and their cell viability was assessed. Cell viability was expressed as the % ratio of cells treated with scramble in the absence of the compound. A representative experiment is depicted here (*n* = 3). Asterisks denote statistically significant differences in comparison to the respective scramble (Student’s *t*-test, *p* < 0.05).

As mentioned above, multiplex immunoassays revealed that HSP27 is highly phosphorylated in MCF7 breast cancer cells as a response to ST61. Indeed, the important role of HSP27 in the protection of MCF7 cells was confirmed after its inhibition by OGX-427 (an antisense oligonucleotide designed to bind to HSP27 mRNA), which significantly enhanced MCF7 cells’ vulnerability in untreated and ST61-treated cells (Figure 6).

**Figure 7:**
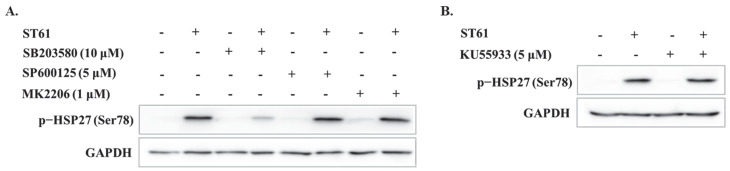
Effect of p38 MAPK, SAPK/JNK, Akt and p53 inhibition on ST61-induced HSP27 phosphorylation in MCF7 breast cancer cells. (**A**) Cells were pre-incubated with 10 μΜ of the p38 MAPK inhibitor SB203580, 5 μΜ of the SAPK/JNK inhibitor SP600125 and 1 μM of the Akt inhibitor MK2206 for 1 h before their exposure to ST61 at its 24 h IC_50_ for 24 h, protein extraction and Western blot analysis using a primary antibody raised against the phosphorylated form of HSP27 (p-HSP27). (**B**) Cells were pre-incubated with 5 μΜ of the ATM inhibitor KU55933 for 1 h before their exposure to ST61 at its 24 h IC_50_ for 3 h, protein extraction and Western blot analysis using a primary antibody raised against the phosphorylated form of HSP27 (p-HSP27, *n* = 2). Glyceraldehyde 3-phosphate dehydrogenase (GAPDH) served as the loading control. Original images can be found in Supplementary Materials.

To elucidate whether HSP27 is a downstream target of the proteins also confirmed to be activated by the compound (i.e., of p38 MAPK, SAPK/JNK, Akt and p53), MCF7 cells were exposed to ST61 in the presence of specific pharmacological inhibitors for each kinase in question (i.e., SB203580, SP600125 and MK2206 for p38 MAPK, SAPK/JNK and Akt, respectively) or for an upstream kinase regulator (i.e., the ATM kinase inhibitor KU55933 for p53, after it was first validated that ST61-induced p53 phosphorylation is indeed mediated by ATM kinase, as shown in Supplementary Figure S16). While SAPK/JNK, Akt and the ATM-p53 axis were not found to be involved in ST61-induced HSP27 phosphorylation, p38 MAPK inhibition markedly reduced ST61-induced HSP27 activation to a high degree, with HSP27 phosphorylation levels almost reaching basal ones (Figure 7A,B and Figure S17). Interestingly, the molecular mechanism underlying ST61-induced cytotoxicity and HSP27 phosphorylation (in which p53 does not participate) seems to be different from the one regulating the p53-dependent ST61-induced G1 arrest of the cell cycle in MCF7 cells described above. ST61-mediated induction of the p38 MAPK/HSP27 axis was also shown in A-431 epidermoid squamous carcinoma cells (Supplementary Figure S18).

### 3.5. ST61’s Anti-Cancer Activity Is Mediated by Oxidative Stress

**Figure 8:**
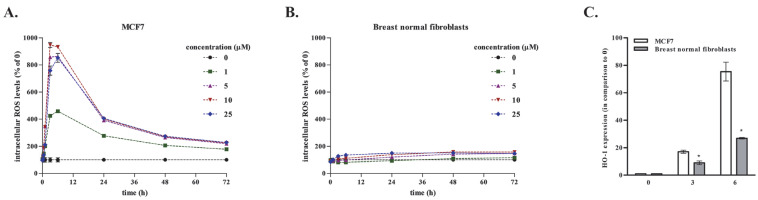
Increase in intracellular ROS levels in ST61-treated MCF7 breast cancer cells. (**A**,**B**) MCF7 cells and normal breast fibroblasts, respectively, were incubated with 10 μΜ of DCFH-DA before the addition of ST61 at concentrations ranging from 0 to 25 μΜ, and the estimation of intracellular levels of reactive oxygen species (ROS) was conducted by recording fluorescence (excitation wavelength: 485 nm, emission wavelength: 520 nm). ROS production was expressed as the % ratio of the untreated control (0 μΜ). A representative experiment (*n* = 3, conducted in triplicate) is presented here. (**C**) MCF7 cells and normal breast fibroblasts were treated with ST61 at its 24 h IC_50_ before sample collection and RNA extraction 0, 3 and 6 h post-exposure. Extracted RNA was then used for RT-qPCR analysis for HO-1, with glyceraldehyde 3-phosphate dehydrogenase (GAPDH) serving as the reference gene. Numerical values are the means ± standard deviations (*n* ≥ 2, conducted in duplicate). Asterisks denote statistically significant differences between MCF7 and normal breast fibroblasts (Student’s *t*-test, *p* < 0.05).

Given that both p38 MAPK and HSP27 are known to be activated by oxidative stress, we explored the hypothesis of ST61-induced cytotoxicity in MCF7 breast cancer cells being mediated by elevated levels of ROS. As shown in Figure 8A, ST61 caused a dose-dependent increase in intracellular ROS levels in MCF7 cancer cells that reached their maximum values at concentrations ≥ 5 μΜ. The effect was considerably smaller in normal breast fibroblasts (Figure 8B). In accordance, a markedly higher induction of the oxidative-stress-inducible enzyme heme oxygenase 1 (HO-1) gene expression was observed in MCF7 cells compared to normal breast fibroblasts (Figure 8C). Interestingly, ST61 was also found to elevate ROS levels in all other cancer cell lines tested, i.e., in A-431 epidermoid squamous carcinoma, A549 non-small-cell lung cancer, MDA-MB-231 triple-negative breast cancer and T24 bladder cancer cells (Supplementary Figure S19).

**Figure 9:**
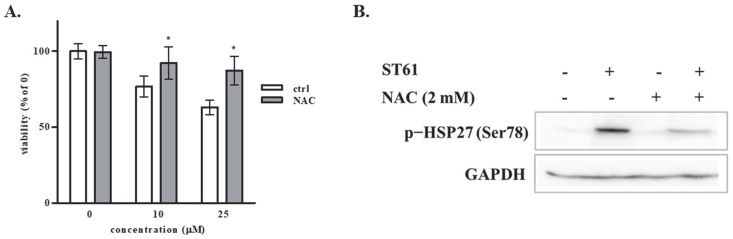
Reactive oxygen species (ROS) scavenging reduces ST61-induced cytotoxicity in MCF7 breast cancer cells. (**A**) Cells were pre-incubated with 2 mM of N-acetyl-cysteine (NAC) for 16 h and exposed to 10 and 25 μΜ of ST61 for 24 h before aspiration of the culture medium and incubation with 1 mg/mL MTT solution for another 4 h at 37 °C. Optical density of the solubilized formazan crystals was measured at 550 nm, and cell viability was calculated as a percentage of the untreated control. Data presented are mean values ± standard deviations (*n* = 2, conducted in triplicate). Asterisks denote statistically significant differences in comparison to the respective sample without NAC (Student’s *t*-test, *p < 0.05). (**B**) Cells were pre-incubated with 2 mM of NAC for 16 h before their exposure to ST61 at its 24 h IC_50_ for 6 h, protein extraction and Western blot analysis using a primary antibody raised against the phosphorylated form of HSP27 (p-HSP27, *n* = 2). Glyceraldehyde 3-phosphate dehydrogenase (GAPDH) served as the loading control. Original images can be found in Supplementary Materials.

To confirm that ST61 exerts its anti-cancer activity through enhanced ROS production, cells were pre-incubated with the known ROS scavenger N-acetyl-cysteine (NAC) before their exposure to 10 and 25 μΜ of the compound for 24 h. Indeed, pre-incubation with NAC highly restricted ST61-induced cytotoxicity in MCF7, A-431, A549, MDA-MB-231 and T24 cells (Figure 9A and Figure S20). In the absence of the main cytotoxic trigger, HSP27 phosphorylation seemed to be no longer required for the protection of the cells, as shown in Figure 9B and Figure S21.

## 4. Discussion

In the present study, a selected well-defined compound set from the structurally diverse chemical library of OPENSCREEN-GR was subjected to an in vitro structure-agnostic bioactivity-driven screening, focusing on anti-cancer activity. Biological evaluation was deliberately blind to provide unbiased insight into structures and/or scaffolds exhibiting the desired function. Four compounds out of the 95 tested ones stood out for their broad anti-cancer activity, since they were found to be highly cytotoxic against seven cancer cell lines deriving from diverse types of cancer, i.e., from epidermoid squamous carcinoma, non-small-cell lung cancer, breast cancer, fibrosarcoma, bladder cancer and metastatic melanoma, and they were then identified as phenoxazine derivatives. This finding is in accordance to the cytotoxic activity reported for phenoxazine compounds against glioblastoma cell lines [29], leukemia cells [30], and colorectal- and breast-cancer-derived cell lines [31].

The two most potent phenoxazine derivatives, ST61 and BS115, were further evaluated regarding their mode of anti-cancer action against three cancer cell lines of different origin (breast and bladder cancer) and different molecular profile (bearing a wild-type or mutant p53). In addition to their cytotoxic activity, compounds ST61 and BS115 were shown here to arrest all three cancer cell lines at the G2/M phase of their cell cycle, irrespective of their p53 status, as also previously shown for glioblastoma [29] and endometrial adenocarcinoma cells [32]. Interestingly, ST61- and BS115-treated MCF7 breast cancer cells—which possess a wild-type functional p53—were found to activate the G1 checkpoint of their cell cycle as well, which is in agreement with the G1 arrest previously reported for 2-aminophenoxazine-3-one-treated G-361 melanoma cells (also having a wild-type p53 status) [33]. The p53-dependent ST61-induced G1 arrest in cancer cells seemed to be a distinct response from the p53-independent ROS-mediated cytotoxicity provoked by the compound.

We subsequently investigated the mechanism of action of the most potent anti-cancer compound ST61 in wild-type p53-bearing MCF7 breast cancer cells. ST61 showed a 72 h IC_50_ value in the low micromolar range (~2 μM) and exhibited a much greater selectivity index for MCF7 cells compared to normal breast fibroblasts, the latter being almost entirely unaffected by a 24 h treatment with the compound even at a concentration of 100 μM and displaying a >10-fold higher 72 h IC_50_ value. In favor of the compound’s selective activity against cancer cells, ST61 showed a 72 h IC_50_ value in the low micromolar range for MDA-MB-231, A-431 and A549 cancer cells as well, while displaying a much higher 72 h IC_50_ value for normal human skin fibroblasts (Supplementary Table S2).

A kinetics analysis using two multiplex immunoassays pointed to HSP27 as a key molecule in the response of MCF7 to ST61’s cytotoxic effect. HSP27 is a stress-induced molecular chaperone that controls many cancer cell pro-survival and growth mechanisms such as apoptosis, autophagy, metastasis, angiogenesis and epithelial-to-mesenchymal transition [34] and has been found to be highly expressed and correlated with poor prognosis in bladder, prostate, breast and colorectal malignancies. Furthermore, HSP27 is known to be induced by cytotoxic agents in many cancers, conferring resistance to chemotherapy [35,36,37], while phosphorylated HSP27 has been recently reported to promote doxorubicin resistance in breast cancer cells by regulating dual phosphorylation of c-Myc [38]. In addition, phosphorylation levels of HSP27 at Ser78 have been shown to be elevated in gemcitabine-resistant in comparison to gemcitabine-sensitive pancreatic cancer cells [39], suggesting that phosphorylation in addition to enhanced expression may also be associated with the protection of neoplastic cells from stress. Thus, HSP27 depletion has been shown to reduce cell viability and to sensitize human colon carcinoma cells to doxorubicin [40], pancreatic cancer cells to gemcitabine [41] and lung cancer cells to erlotinib [36]. In the current study, inhibition of HSP27 in MCF7 breast cancer cells using the antisense oligonucleotide OGX-427 significantly increased their intrinsic vulnerability as well as their sensitivity to ST61 due to the decreased HSP27 expression and phosphorylation, since phosphorylated HSP27 at Ser78 may serve as a pro-survival stress response in cancer cells and thus be responsible for the enhancement of ST61-induced cytotoxicity by HSP27 depletion. Increased sensitivity provoked by HSP27 inhibition is in full accordance with previously reported data for the chemoresistant human pancreatic cancer cell line MiaPaCa-2, which became more sensitive to gemcitabine [41], and for the human lung cancer cell lines A549 and HCC827, which were sensitized to erlotinib [36].

ST61-induced HSP27 phosphorylation at Ser78 was abolished in MCF7 and A-431 cells when p38 MAPK activation was inhibited by its selective pharmacological inhibitor SB203580, evidencing that HSP27 phosphorylation is mainly regulated by p38 MAPK in ST61-treated breast and epidermoid cancer cells. This finding is in agreement with previous studies showing that HSP27 Ser78 phosphorylation is mainly regulated by the HER-2/*neu*-p38 MAPK pathway in the BT474 breast cancer cell line treated with heregulin α1 [42] and that MAPKAPK2 and HSP27 are downstream effectors of p38 MAPK in PC3 human prostate cancer cells [43]. In addition, p38 MAPK inhibition has been reported to reduce HSP27 activation and the hypoxia-mediated increase in androgen receptor activity in castration-resistant prostate cancer (CRPC) cells, while inhibition of p38 MAPK activity decreased proliferation and survival of CRPC cells in vitro and prolonged survival of tumor-bearing mice [44].

**Figure 10:**
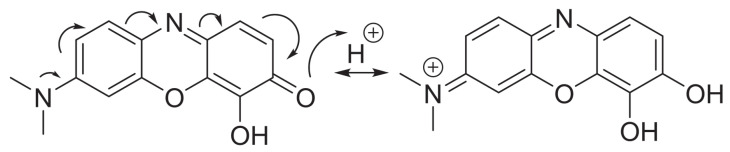
ST61 possesses phenol groups that can participate in redox reactions.

p38 MAPK has been shown to be phosphorylated by mitochondrial ROS in cardiomyocytes during hypoxia [45], and oxidative-stress-induced p38 MAPK phosphorylation has been linked to the activation of caspase-8- and -9-mediated apoptotic pathways in dopaminergic neurons [46]. On the other hand, HSP27 has been shown to regulate intracellular redox homeostasis and enhance cellular resistance to oxidative damage by protecting against lipid peroxidation and protein oxidation in cardiac cells [47,48] and to be a key mediator of prolactin-induced pancreatic beta-cell cytoprotection against oxidative stress [49]. Thus, we investigated the possibility that ST61 exerts its anti-cancer action via increased ROS production. Indeed, treatment with ST61 resulted in a dose-dependent increase in intracellular ROS levels in MCF7 breast cancer cells, whereas most importantly, only slight ST61-induced oxidative stress was observed in normal breast fibroblasts. Accordingly, the antioxidant response of normal breast fibroblasts was much more attenuated in comparison to that of breast cancer cells, as shown by their lower up-regulation of a key downstream target of the transcription factor Nrf2, i.e., HO-1 [50,51]. The subcellular source of ST61-induced ROS in cancer cells (e.g., mitochondria, NADPH oxidases, or redox cycling of ST61 itself) remains to be investigated. In favor of the oxidative-stress-mediated ST61 anti-cancer activity, pre-incubation of MCF7 cells with the well-known ROS scavenger NAC confined ST61’s cytotoxic effect, while in the absence of the oxidative stress stimulus, HSP27 was no longer phosphorylated at Ser78 in MCF7 breast cancer cells. The ROS-mediated anti-cancer activity of ST61 was also confirmed here in epidermoid carcinoma, lung cancer and bladder cancer cells and could be attributed to the structure of phenoxazine ST61, having both proton-donating and proton-accepting groups, along with a conjugated chromophoric system (Figure 10) and possessing phenol functionalities that exhibit unique redox properties, leading to ROS production [52].

Given that viability assessment by the MTT assay is based on cells’ mitochondrial functionality (which could be affected by ST61-mediated oxidative stress), the cytotoxic effect of the compound and the protective role of NAC were further confirmed here using an ROS-independent neutral red uptake assay [21], which estimates viability based on the general metabolic activity of the cells (Supplementary Figure S22). Phenoxazine-mediated ROS generation has been previously reported in the human glioblastoma cell line LN229 [53] and in RKO colorectal and MCF7 breast cancer cells [31]. It should be noted that therapeutic strategies aiming at enhancing ROS production hold promise for selectively eliminating cancer cells, since their antioxidant defenses are overwhelmed by their typically excessive basal ROS levels [54].

## 5. Conclusions

In conclusion, the phenoxazine derivative ST61, emerging in the current study from the in vitro bioactivity-driven screening of a series of compounds from the chemical library of the national research infrastructure OPENSCREEN-GR, proved to display a broad and selective anti-cancer activity with an IC_50_ in the low micromolar range, which was shown to be mediated by the production of ROS. Cancer cells responded to ST61-induced oxidative stress by the activation of a p38 MAPK-HSP27 axis. Our in vitro data support the notion that ST61 may represent a promising anti-cancer compound in various types of cancer, as a monotherapy or in a combinatorial treatment scheme, along with an HSP27 inhibitor.

## Figures and Tables

**Table 1:** Summary of the cytotoxic activity of compounds **1A**–**48A** at 10 μΜ in A-431, A549, MCF7, MDA-MB-231, HT-1080, T24 and WM-266-4 cancer cells treated with the compounds for 72 h.

	Cell Line	A-431	A549	MCF7	MDA-MB-231	HT-1080	T24	WM-266-4			
Compound											
**1A**											
**2A**											
**3A**											
**4A**											**viability (%)**
**5A**											**<20**
**6A**											**21–40**
**7A**											**41–60**
**8A**											**61–80**
**9A**											**>80**
**10A**											
**11A**											
**12A**											
**13A**											
**14A**											
**15A**											
**16A**											
**17A**											
**18A**											
**19A**											
**20A**											
**21A**											
**22A**											
**23A**											
**24A**											
**25A**											
**26A**											
**27A**											
**28A**											
**29A**											
**30A**											
**31A**											
**32A**											
**33A**											
**34A**											
**35A**											
**36A**											
**37A**											
**38A**											
**39A**											
**40A**											
**41A**											
**42A**											
**43A**											
**44A**											
**45A**											
**46A**											
**47A**											
**48A**											

## Data Availability

All data supporting reported results can be found within the article and the Supplementary Material.

## Supplementary Materials

The following supplementary materials can be downloaded at: https://www.mdpi.com/article/10.3390/biom16050689/s1, Figure S1: ^1^H-NMR and ^13^C-NMR spectra for BS125; Figure S2: Purity and ESI-MS analysis of BS125; Figure S3: ^1^H-NMR and ^13^C-NMR spectra for BS115; Figure S4: Purity and ESI-MS analysis of BS115; Figure S5: Purity and ESI-MS analysis of BS74; Figure S6: Purity and ESI-MS analysis of ST61; Figure S7: Cytotoxic activity of compounds **1A**–**48A** and **1B**–**47B** on human epidermoid squamous carcinoma A-431 cells; Figure S8: Cytotoxic activity of compounds **1A**–**48A** and **1B**–**47B** on human non-small-cell lung cancer A549 cells; Figure S9: Cytotoxic activity of compounds **1A**–**48A** and **1B**–**47B** on human breast cancer MCF7 cells; Figure S10: Cytotoxic activity of compounds **1A**–**48A** and **1B**–**47B** on human breast cancer MDA-MB-231 cells; Figure S11: Cytotoxic activity of compounds **1A**–**48A** and **1B**–**47B** on human fibrosarcoma HT-1080 cells; Figure S12: Cytotoxic activity of compounds **1A**–**48A** and **1B**–**47B** on human bladder cancer T24 cells; Figure S13: Cytotoxic activity of compounds **1A**–**48A** and **1B**–**47B** on metastatic human melanoma WM-266-4 cells; Figure S14: Cell cycle distribution of T24 cancer cells treated with compounds **ST61** and **BS115**; Figure S15: Densitometric analysis from three independent replicates for the quantification of Western blots presented in Figure 5; Figure S16: Western blot analysis of protein extracts from ST61-treated MCF7 breast cancer cells for the phosphorylation of p53 after their pre-incubation with the ATM kinase inhibitor KU55933; Figure S17: Densitometric analysis from two independent replicates for the quantification of the Western blots presented in Figure 7; Figure S18: Western blot analysis of protein extracts from ST61-treated A-431 epidermoid cancer cells for the phosphorylation of p38 MAPK as well as for the phosphorylation of HSP27 in the presence of the p38 MAPK kinase inhibitor SB203580; Figure S19: Increase in intracellular ROS levels in ST61-treated A-431, A549, MDA-MB-231 and T24 cancer cells; Figure S20: Cytotoxic activity of compound ST61 in the presence of N-acetyl-cysteine (NAC); Figure S21: Densitometric analysis from two independent replicates for the quantification of the Western blot presented in Figure 9; Figure S22: Cytotoxic activity of compound ST61 in the presence of N-acetyl-cysteine (NAC), as estimated by the neutral red uptake assay; Table S1: Chemical structures of selected compounds displaying anti-cancer activity in ≥5 of the tested cancer cell lines; Table S2: Calculated ST61 72 h IC_50_ values for MCF7, MDA-MB-231, A-431 and A549 cancer cells as well as for normal human skin and breast fibroblasts. File S1. Original WB images.

## Abbreviations

The following abbreviations are used in this manuscript:

CRPC	castration-resistant prostate cancer
DCFH-DA	2′,7′-dichlorfluorescein-diacetate
DMEM	Dulbecco’s modified eagle medium
DMSO	dimethyl sulfoxide
FBS	fetal bovine serum
GAPDH	glyceraldehyde 3-phosphate dehydrogenase
HO-1	heme oxygenase 1
MFI	median fluorescence intensity
MTT	3-(4,5-dimethylthiazol-2-yl)-2,5-diphenyltetrazolium bromide
NAC	N-acetyl-cysteine
PBS	phosphate-buffered saline
PI	propidium iodide
PVDF	polyvinylidene fluoride
ROS	reactive oxygen species
RT-qPCR	reverse transcription quantitative polymerase chain reaction
TMS	tetramethylsilane
