# Supplementary material for: Phenoxazine Derivative ST61 Displays an Oxidative-Stress-Mediated Cytotoxic Activity Against Human Cancer Cells
Source: Biomolecules. 2026 May 6;16(5):689. doi: 10.3390/biom16050689 (PMC13204190; doi:10.3390/biom16050689)
Supplement: Supplementary file 1 [file biomolecules-16-00689-s001.zip › biomolecules-4275260-supplementary.pdf]

Supporting Information

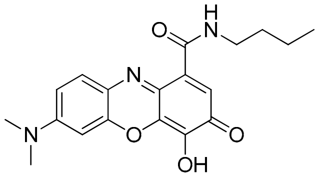

BS125

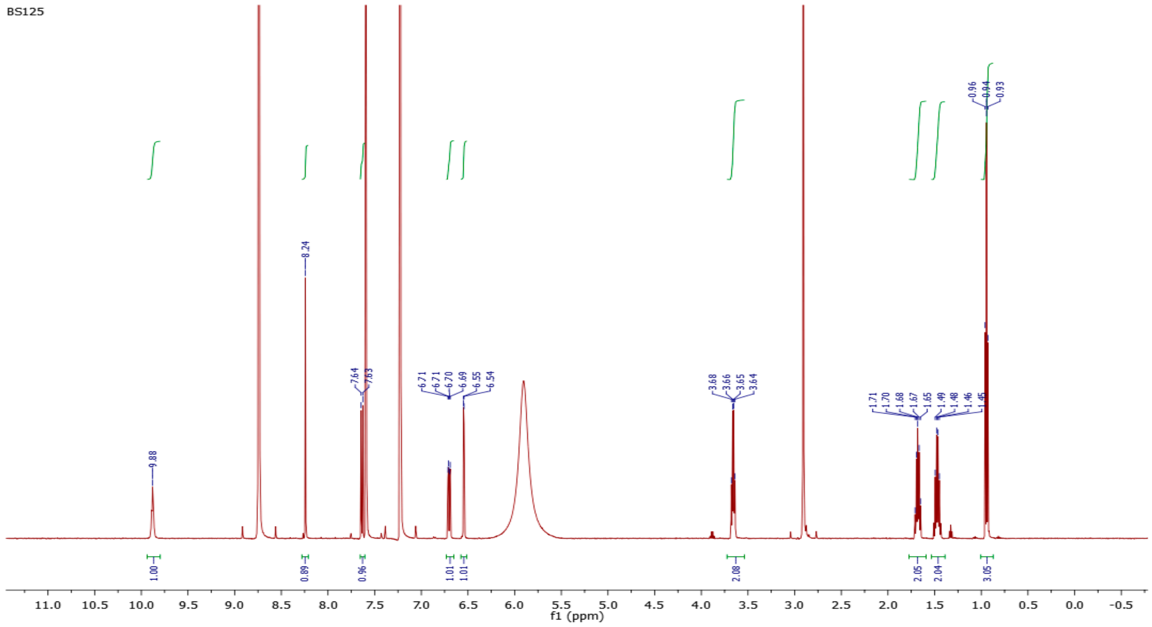

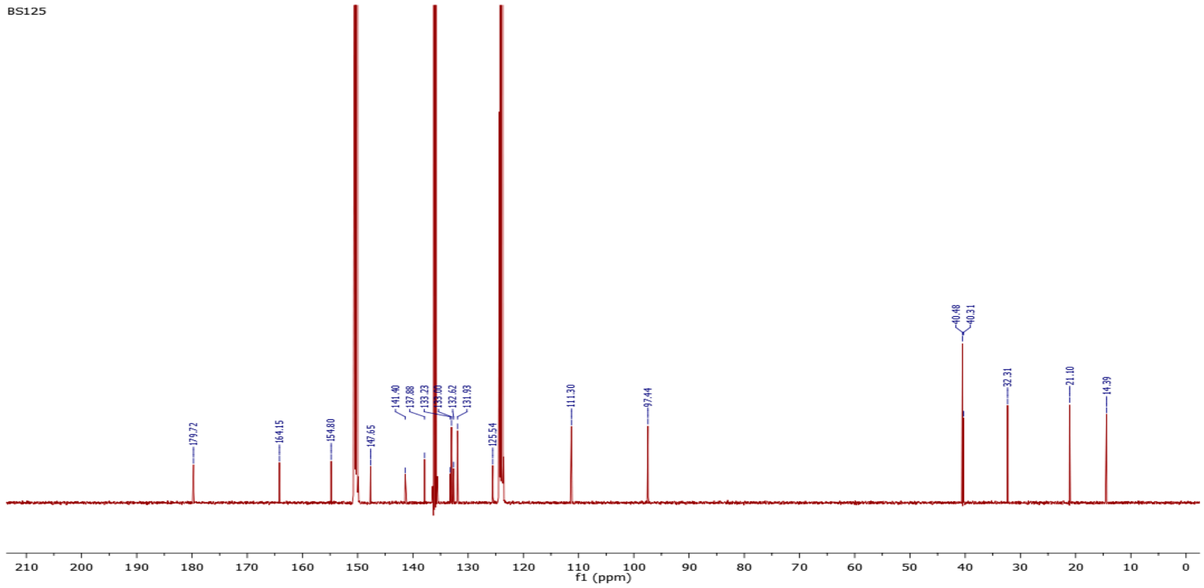

Figure S1. ^{1}H-NMR and ^{13}C-NMR spectra for **BS125**.

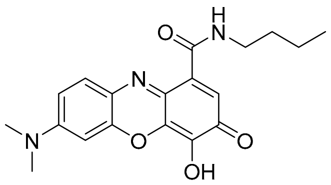

BS125

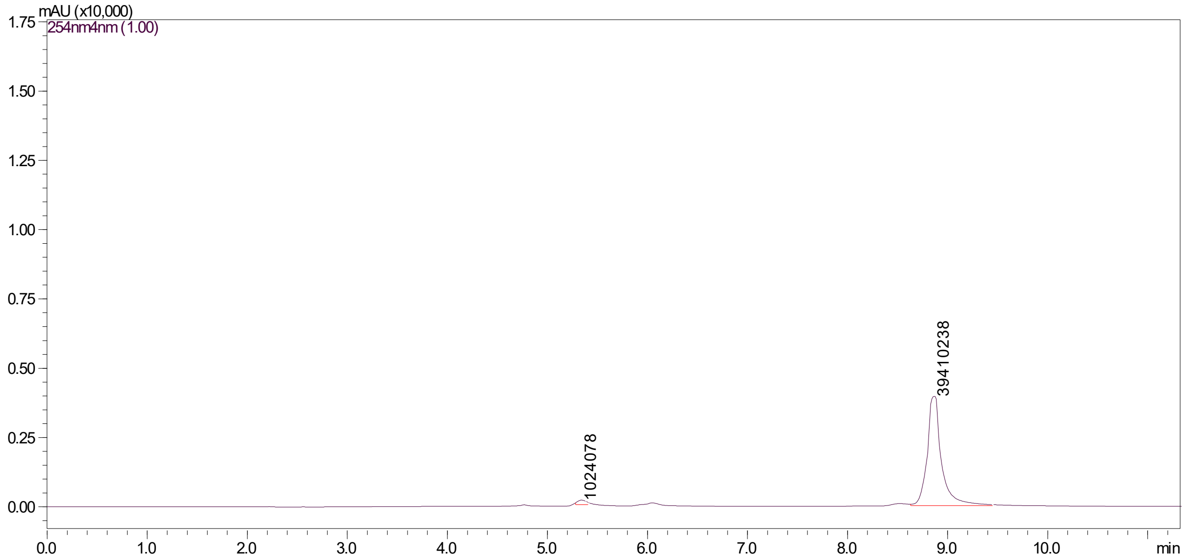

peak 1=1024078
peak 2= 39410238
total=40434316
purity % = (area of peak of interest)/(total area)*100% = (39410238/40434316)*100% = 97.46%
Method= 100% MeOH

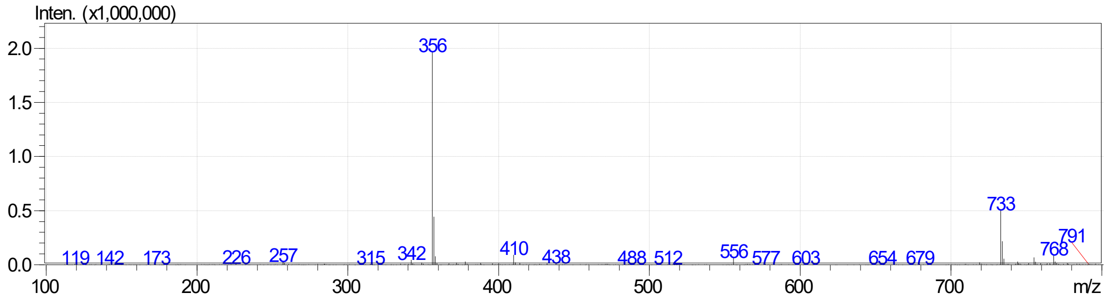

Figure S2. Purity and ESI-MS analysis of BS125. ESI-MS analysis, positive mode: m/z calcd mass for $C_{19}H_{22}N_3O_4^+$ $[M+H]^+$=356,16, was found 356.

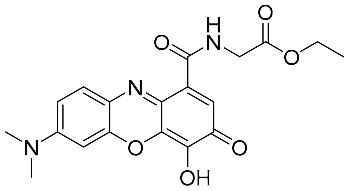

BS115

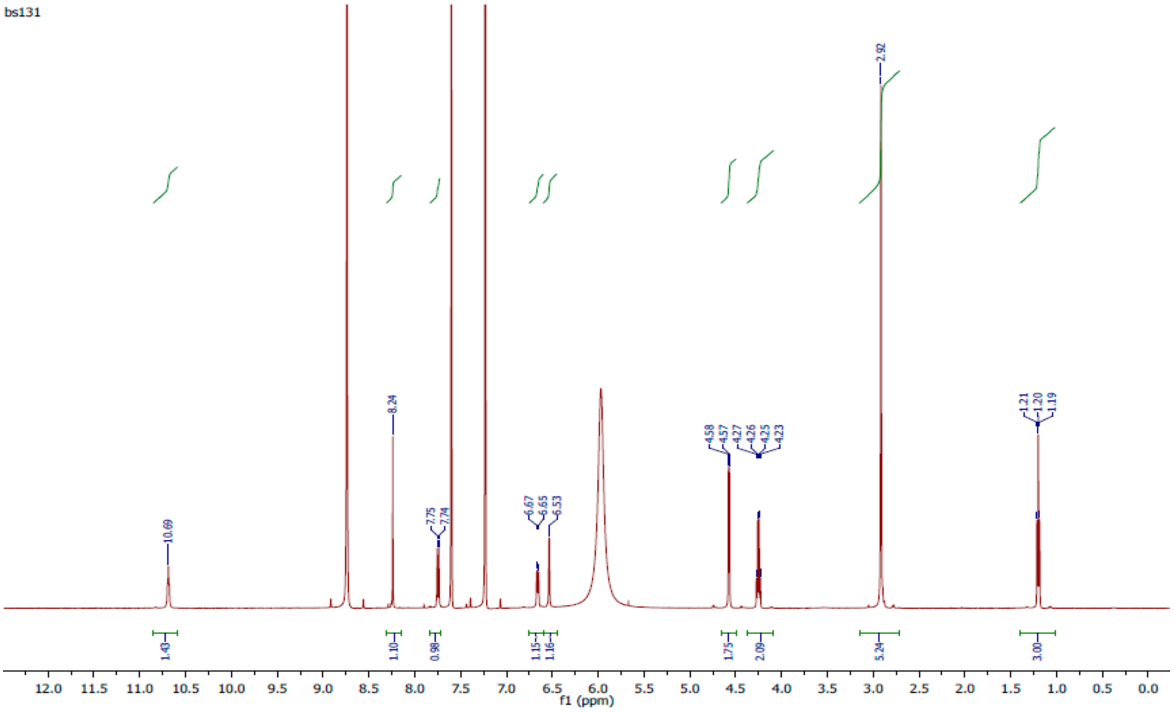

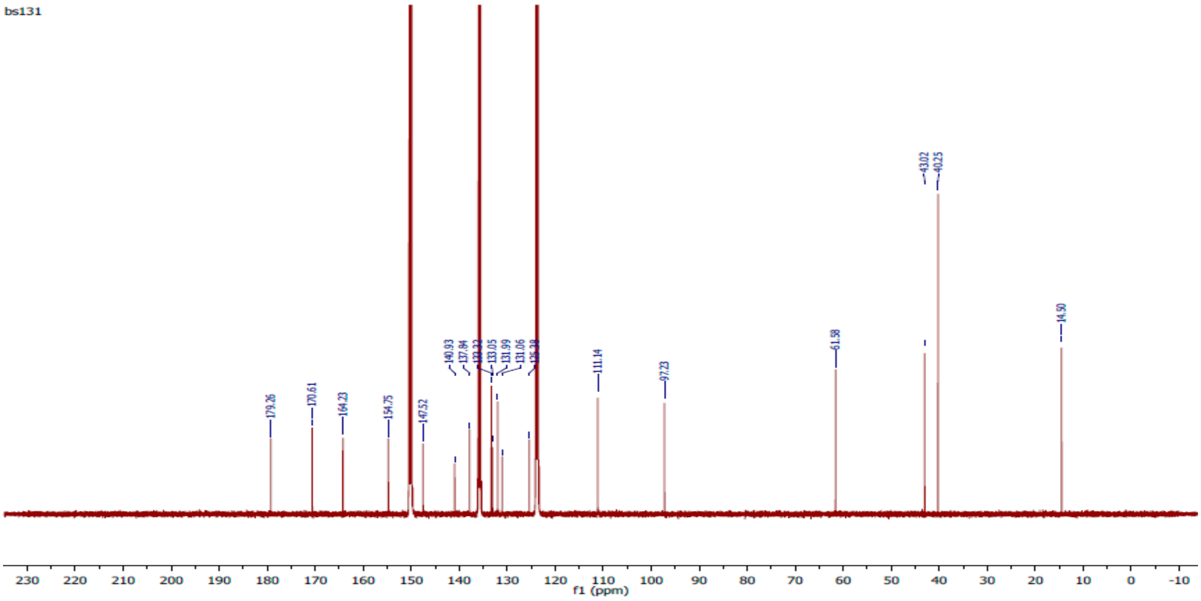

Figure S3. ^{1}H-NMR and ^{13}C-NMR spectra for **BS115**.

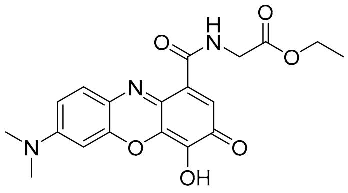

BS115

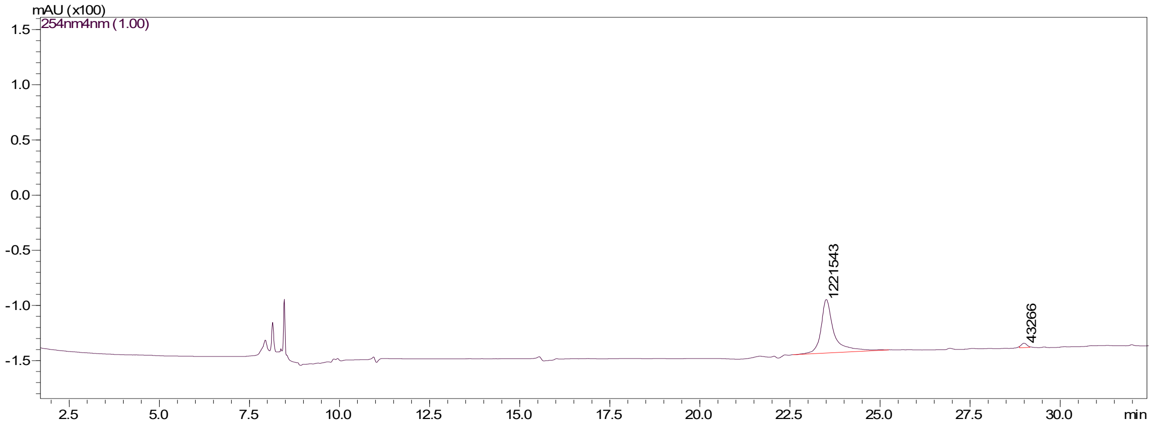

peak 1=1221543

peak 2=43266

total 1264809

purity % = (area of peak of interest)/(total area)*100% = (1221543/1264809)*100% = 96.57%

Method=Pump A CAN; Pump B 90/10 H_2O/CAN

t (min)	A(%)	B(%)
3	0	100
22	86.5	13.5
25	91	9
29	91	9
31	95.5	4.5
37	95.5	4.5
39	0	100
44	0	100

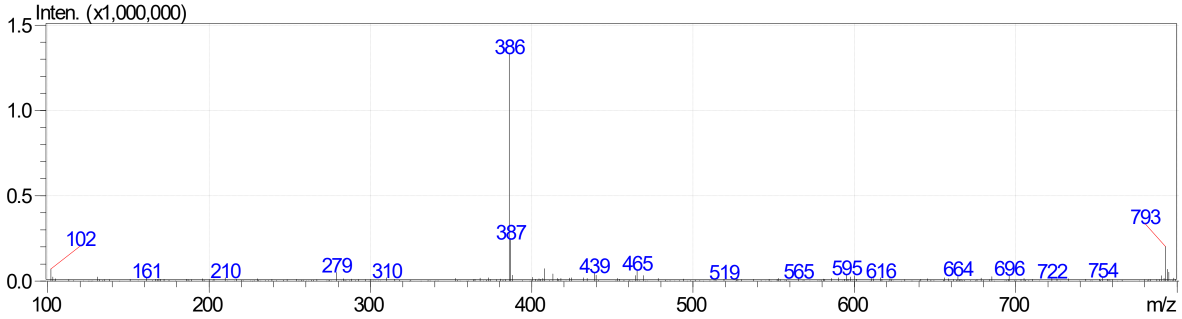

Figure S4. Purity and ESI-MS analysis of BS115. ESI-MS analysis, positive mode: m/z calcd mass for $C_{19}H_{20}N_3O_6^+$ $[M+H]^+$=386,14, was found 385.95.

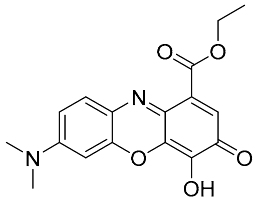

BS74

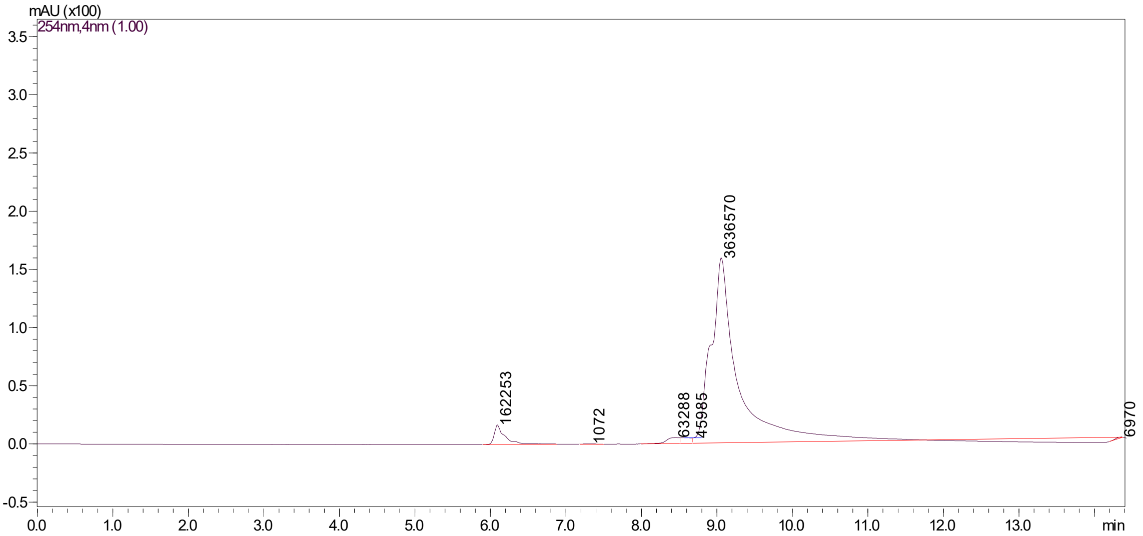

peak 1=162253
peak 2=3636570
total=3798823
purity % = (area of peak of interest)/(total area)*100% = (3636570/3798823)*100% = 95.72%
found 328.85
Method= 100% MeOH

(+)

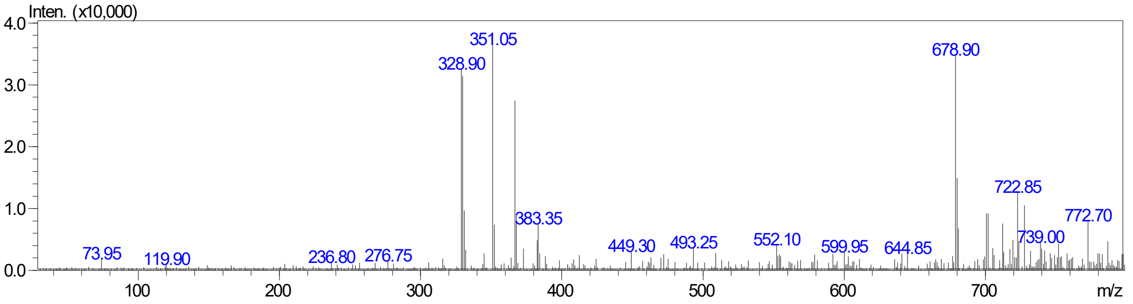

Figure S5. Purity and ESI-MS analysis of BS74. ESI-MS analysis, positive mode: m/z calcd mass for $C_{17}H_{16}N_2O_5^+$ $[M]^+$= 328.32, was found 328.90.

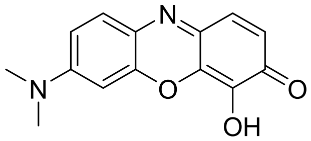

ST61

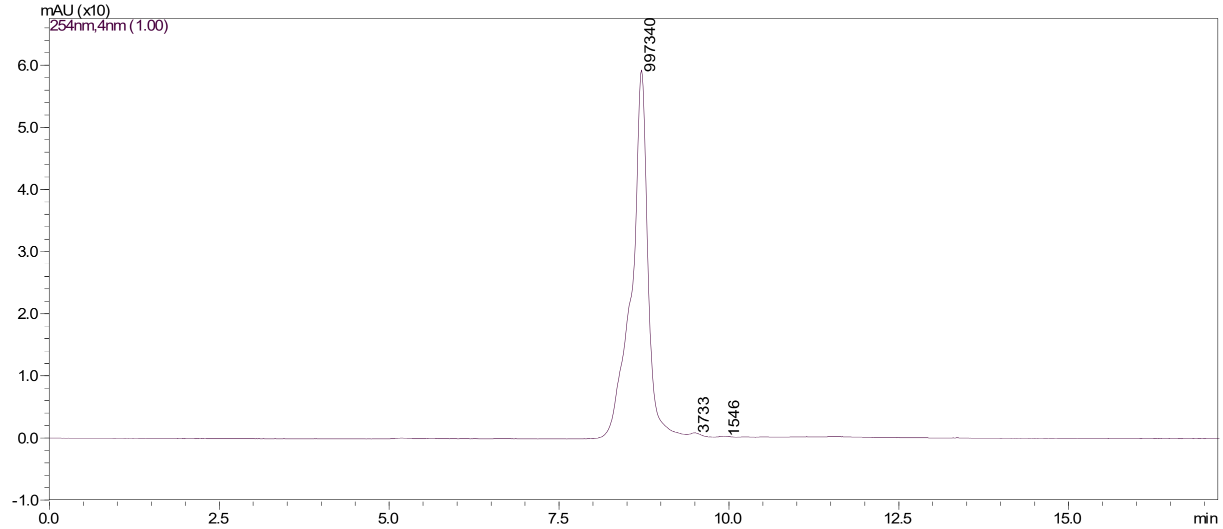

Rt = 8.68 mins

purity % = (area of peak of interest)/(total area)*100% = (997340/1002619)*100% = 99.5%

Method= 100% MeOH

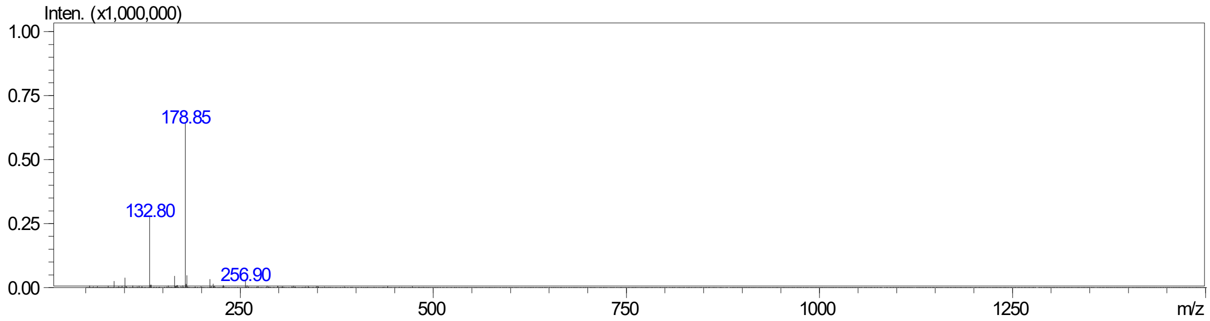

Figure S6. Purity and ESI-MS analysis of ST61. ESI-MS analysis, positive mode: m/z calcd mass for $C_{14}H_{12}N_2O_3^+$ $[M]^+$= 256.26, was found 256.90.

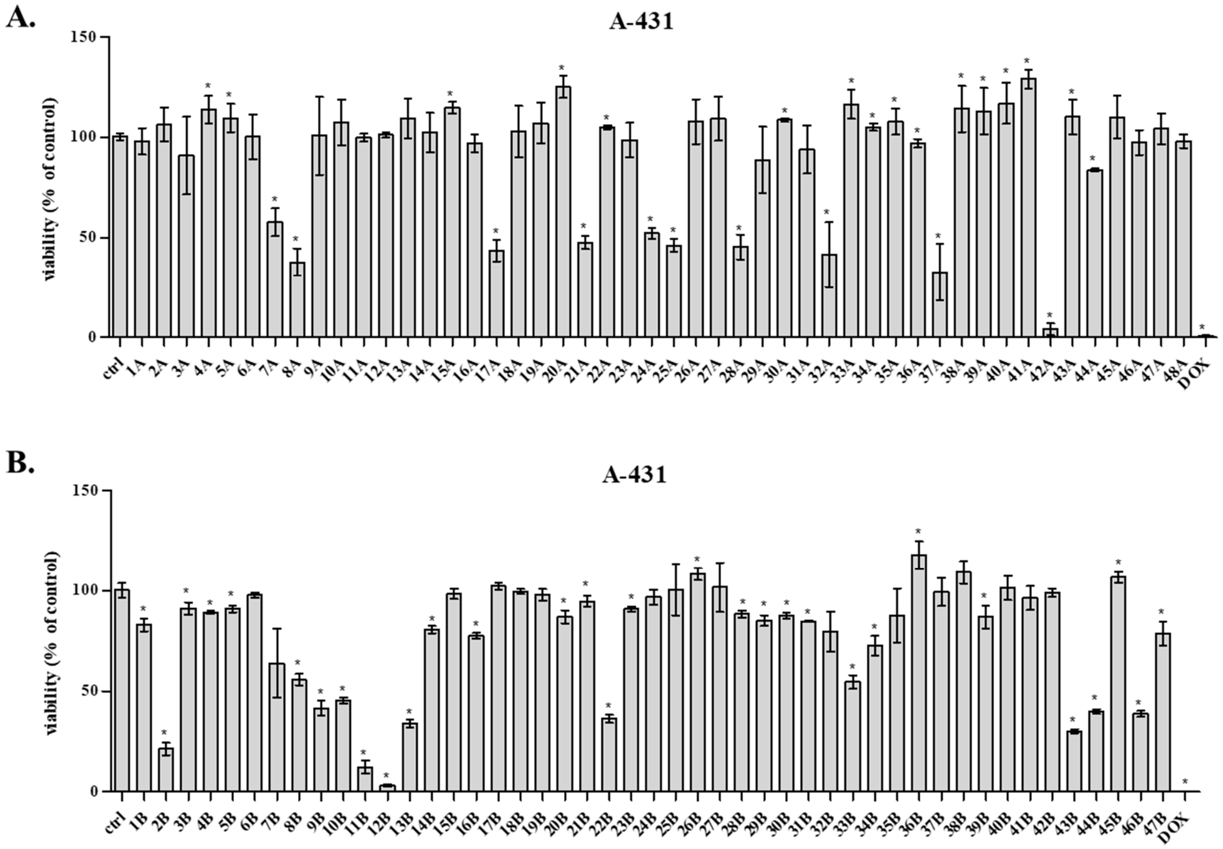

Figure S7. Cytotoxic activity of compounds 1A-48A (A) and 1B-47B (B) on human epidermoid squamous carcinoma A-431 cells. Cells were incubated with 10 μM of the compounds for 72 h before aspiration of the culture medium and incubation with 1 mg/mL MTT solution for another 4 h at 37°C. Optical density of the solubilized formazan crystals (formed only in living cells) was measured at 550 nm and cell viability was calculated as a percent ratio of the untreated control sample. Data presented are mean values ± standard deviations (n=3). The known-anticancer drug doxorubicin (DOX) used at the concentration of 5 μM served as the positive control. Asterisks denote statistically significant differences in comparison to the untreated control (ctrl) for p<0.05 (Student′ t-test).

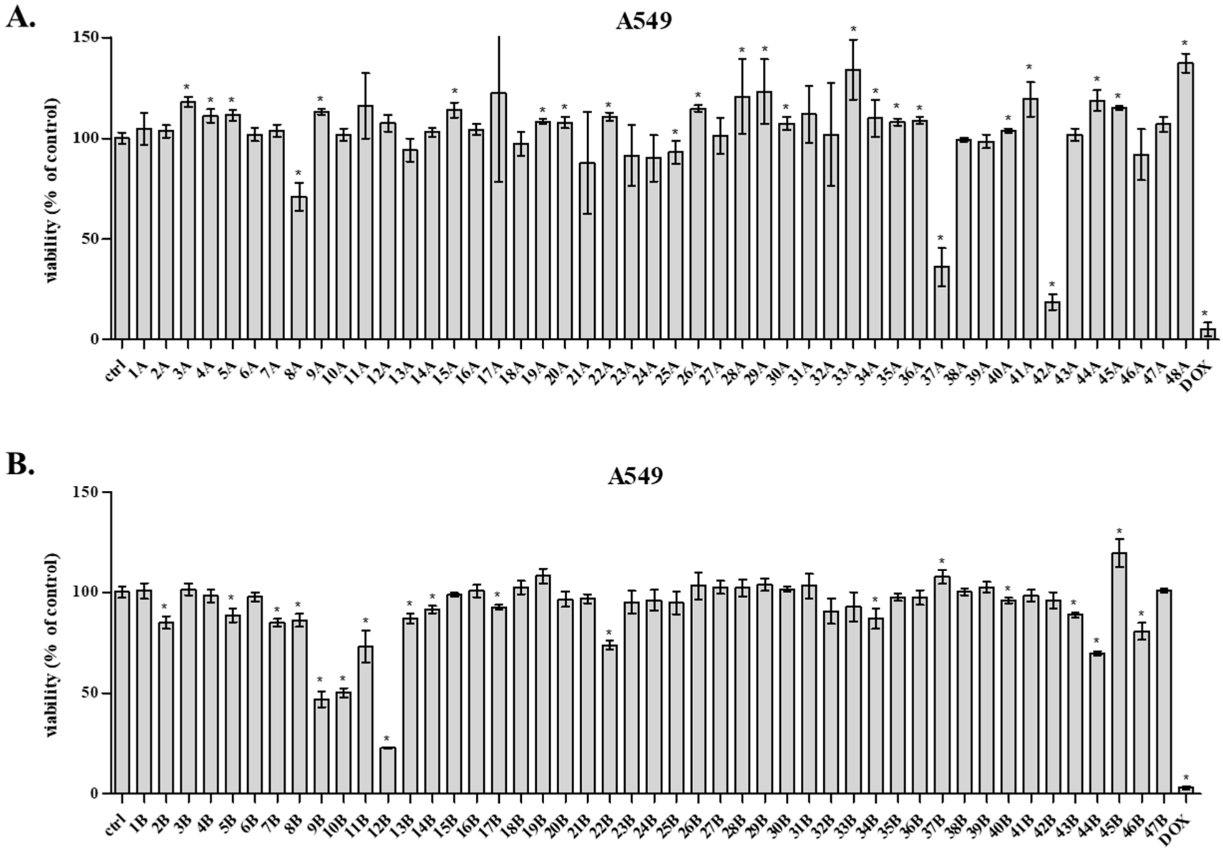

Figure S8. Cytotoxic activity of compounds 1A-48A (A) and 1B-47B (B) on human non-small cell lung cancer A549 cells. Cells were incubated with 10 μM of the compounds for 72 h before aspiration of the culture medium and incubation with 1 mg/mL MTT solution for another 4 h at 37°C. Optical density of the solubilized formazan crystals (formed only in living cells) was measured at 550 nm and cell viability was calculated as a percent ratio of the untreated control sample. Data presented are mean values ± standard deviations (n=3). The known-anticancer drug doxorubicin (DOX) used at the concentration of 5 μM served as the positive control. Asterisks denote statistically significant differences in comparison to the untreated control (ctrl) for $p<0.05$ (Student′ t-test).

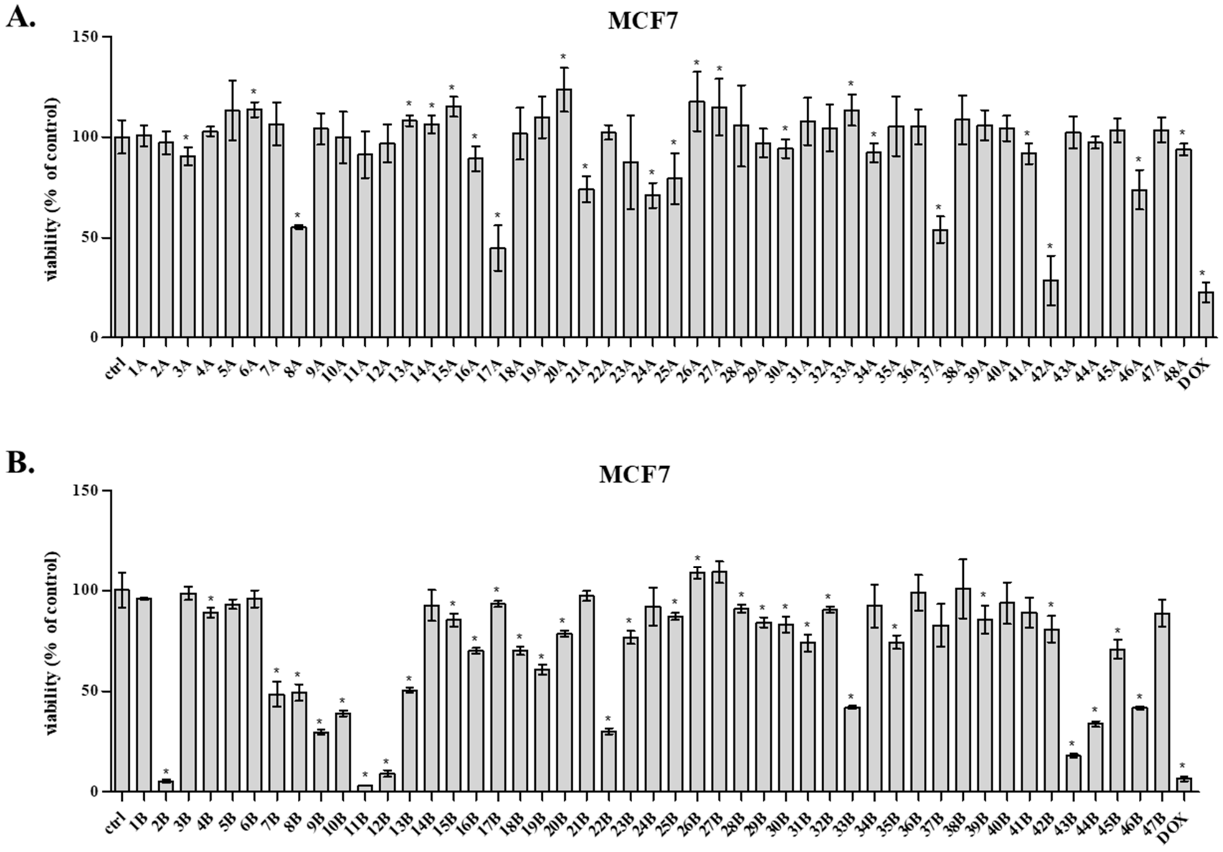

Figure S9. Cytotoxic activity of compounds 1A-48A (A) and 1B-47B (B) on human breast cancer MCF7 cells. Cells were incubated with 10 μM of the compounds for 72 h before aspiration of the culture medium and incubation with 1 mg/mL MTT solution for another 4 h at 37°C. Optical density of the solubilized formazan crystals (formed only in living cells) was measured at 550 nm and cell viability was calculated as a percent ratio of the untreated control sample. Data presented are mean values ± standard deviations (n=3). The known-anticancer drug doxorubicin (DOX) used at the concentration of 5 μM served as the positive control. Asterisks denote statistically significant differences in comparison to the untreated control (ctrl) for p<0.05 (Student' t-test).

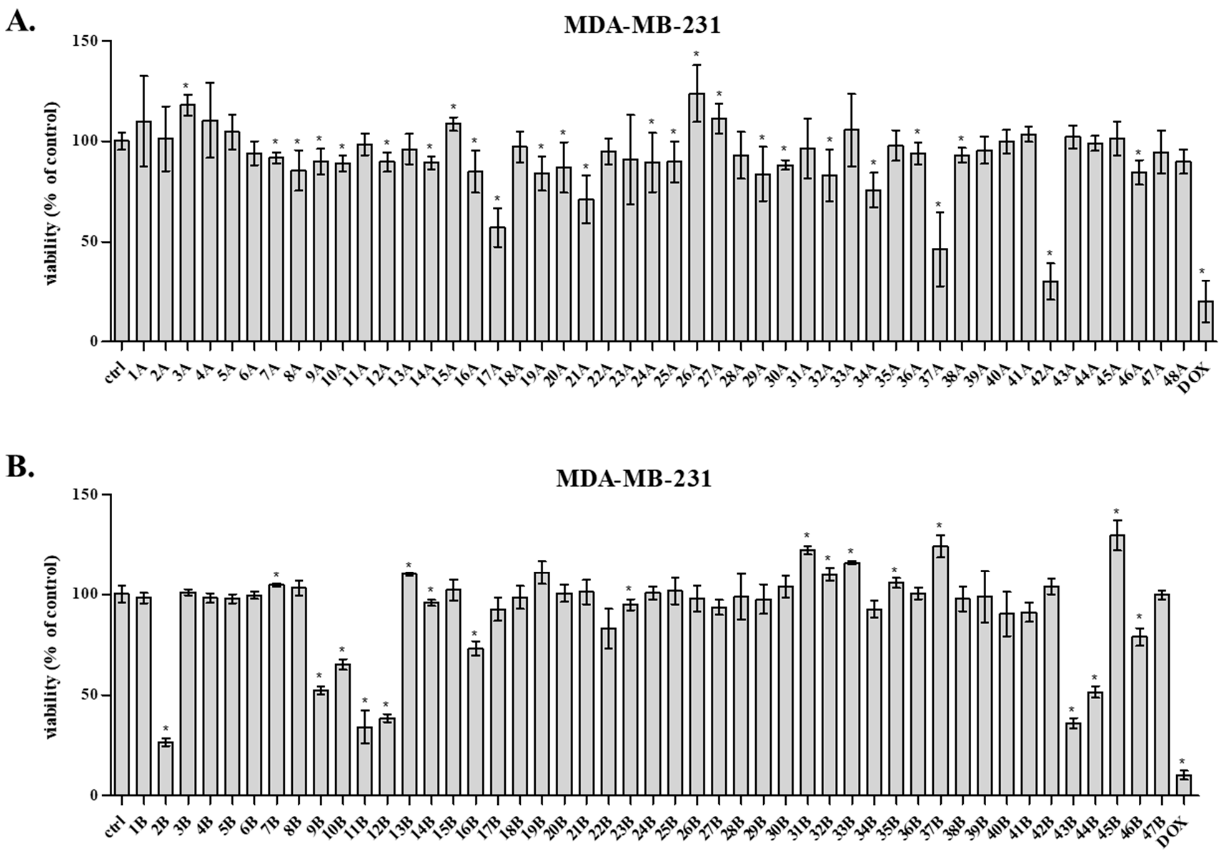

Figure S10. Cytotoxic activity of compounds 1A-48A (A) and 1B-47B (B) on human breast cancer MDA-MB-231 cells. Cells were incubated with 10 μM of the compounds for 72 h before aspiration of the culture medium and incubation with 1 mg/mL MTT solution for another 4 h at 37°C. Optical density of the solubilized formazan crystals (formed only in living cells) was measured at 550 nm and cell viability was calculated as a percent ratio of the untreated control sample. Data presented are mean values ± standard deviations (n=3). The known-anticancer drug doxorubicin (DOX) used at the concentration of 5 μM served as the positive control. Asterisks denote statistically significant differences in comparison to the untreated control (ctrl) for $p<0.05$ (Student' t-test).

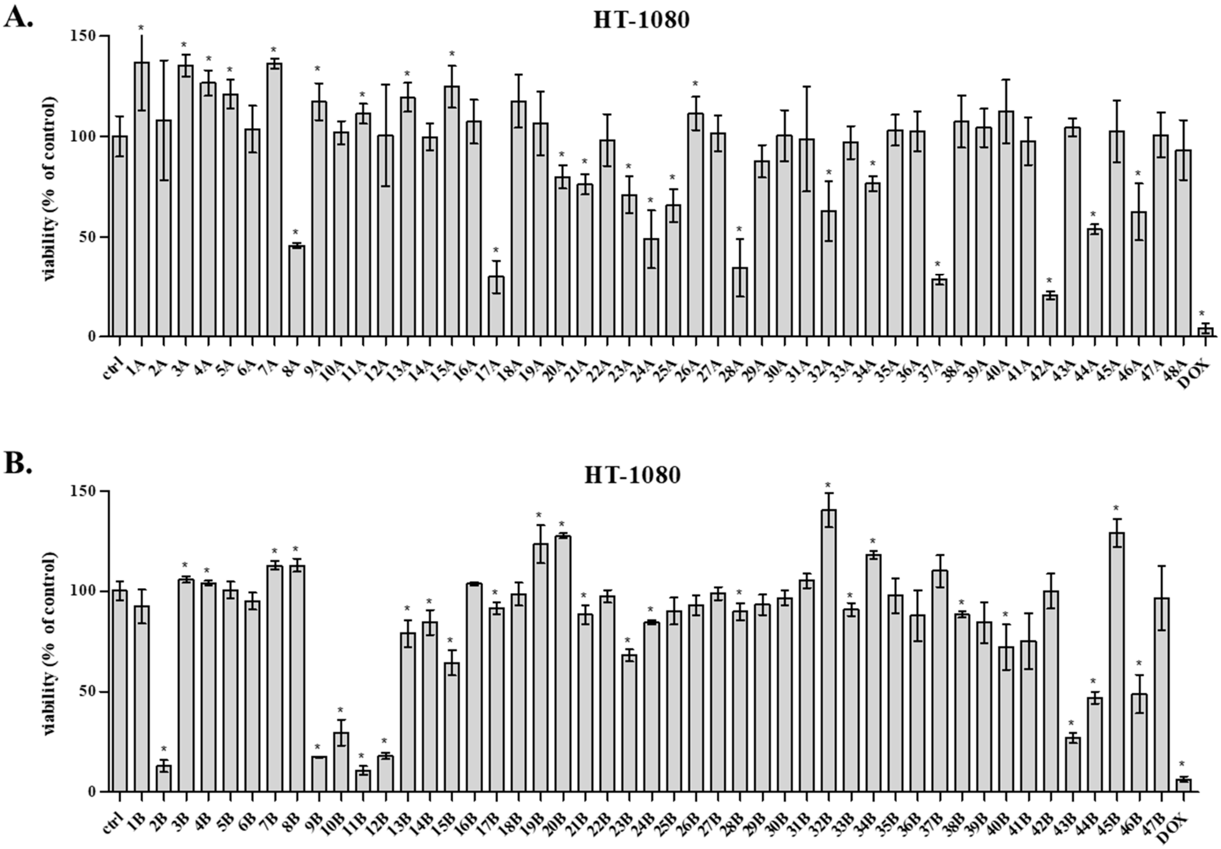

Figure S11. Cytotoxic activity of compounds 1A-48A (A) and 1B-47B (B) on human fibrosarcoma HT-1080 cells. Cells were incubated with 10 μM of the compounds for 72 h before aspiration of the culture medium and incubation with 1 mg/mL MTT solution for another 4 h at 37°C. Optical density of the solubilized formazan crystals (formed only in living cells) was measured at 550 nm and cell viability was calculated as a percent ratio of the untreated control sample. Data presented are mean values ± standard deviations (n=3). The known-anticancer drug doxorubicin (DOX) used at the concentration of 5 μM served as the positive control. Asterisks denote statistically significant differences in comparison to the untreated control (ctrl) for $p<0.05$ (Student′ t-test).

Supplementary Figure S12

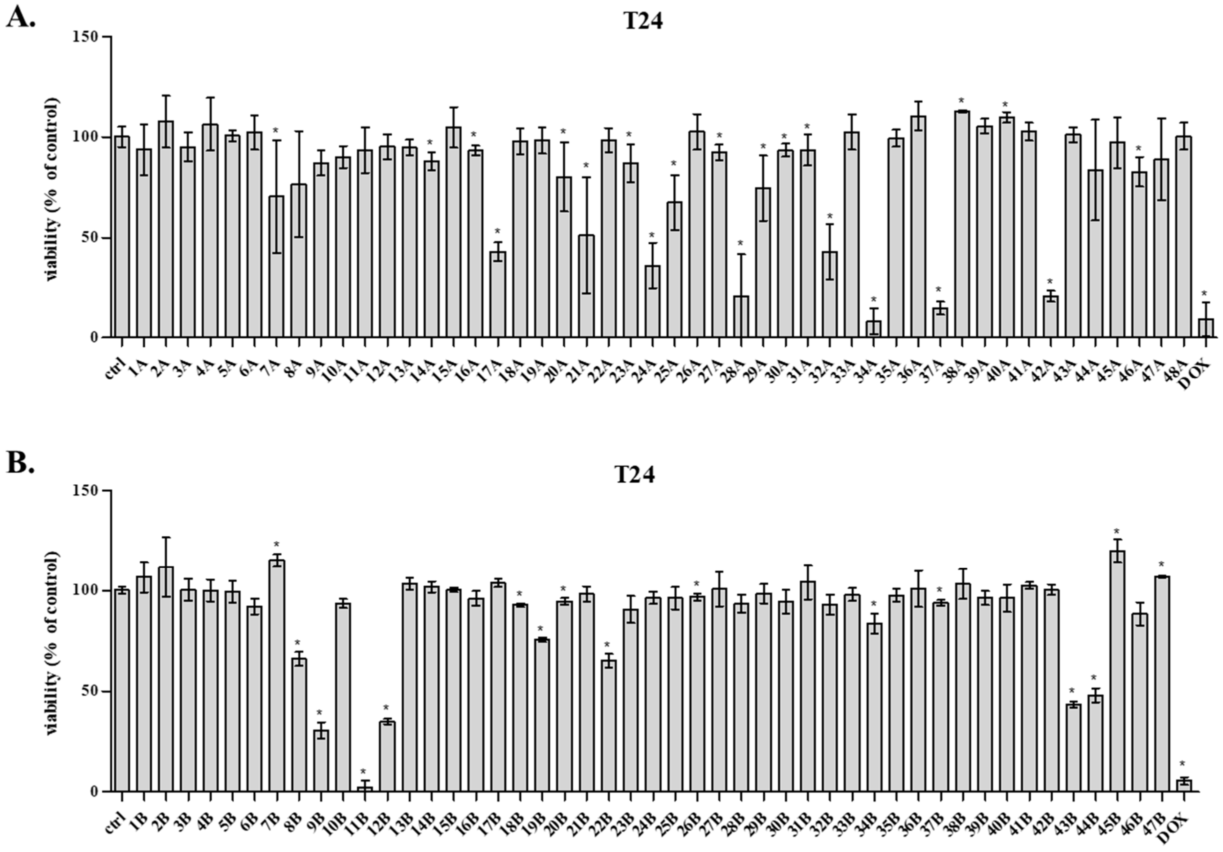

Figure S12. Cytotoxic activity of compounds 1A-48A (A) and 1B-47B (B) on human bladder cancer T24 cells. Cells were incubated with 10 μM of the compounds for 72 h before aspiration of the culture medium and incubation with 1 mg/mL MTT solution for another 4 h at 37°C. Optical density of the solubilized formazan crystals (formed only in living cells) was measured at 550 nm and cell viability was calculated as a percent ratio of the untreated control sample. Data presented are mean values ± standard deviations (n=3). The known-anticancer drug doxorubicin (DOX) used at the concentration of 5 μM served as the positive control. Asterisks denote statistically significant differences in comparison to the untreated control (ctrl) for $p<0.05$ (Student′ t-test).

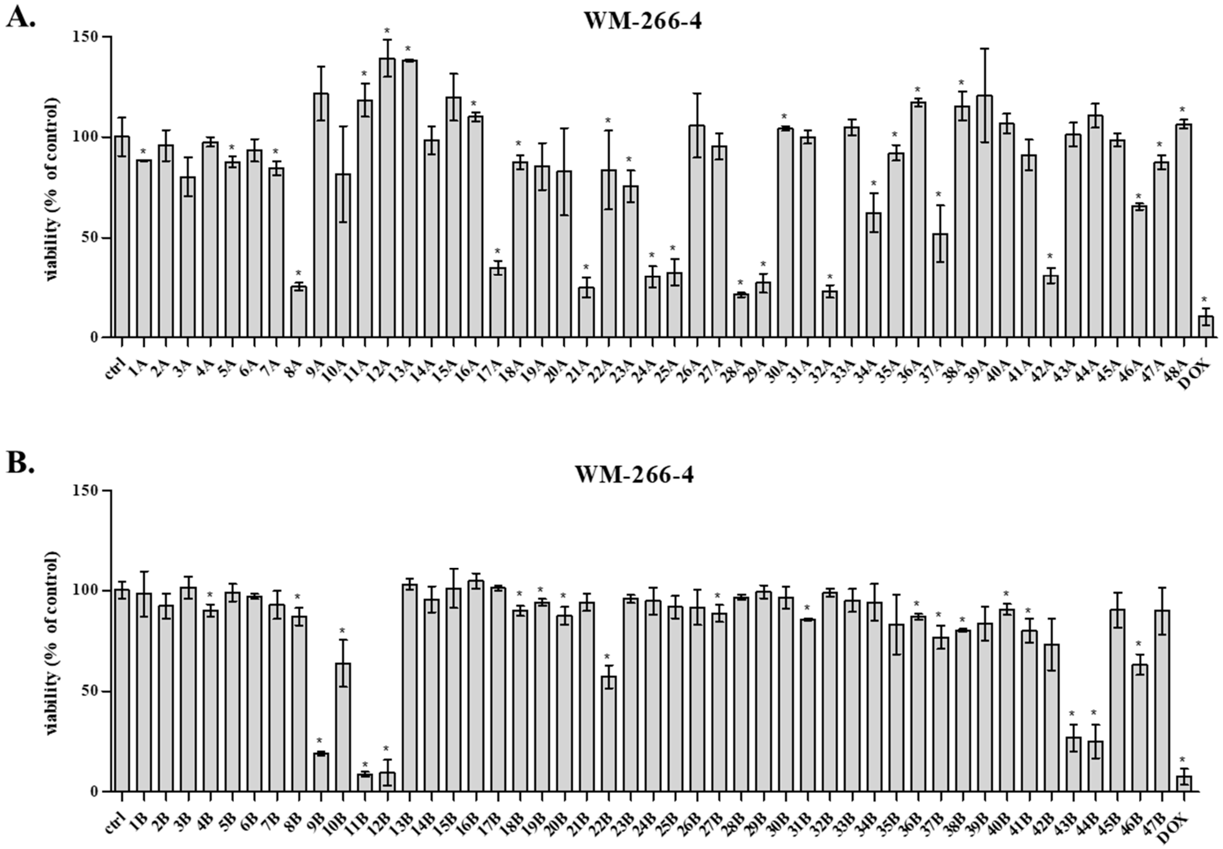

Figure S13. Cytotoxic activity of compounds 1A-48A (A) and 1B-47B (B) on metastatic human melanoma WM-266-4 cells. Cells were incubated with 10 µM of the compounds for 72 h before aspiration of the culture medium and incubation with 1 mg/mL MTT solution for another 4 h at 37°C. Optical density of the solubilized formazan crystals (formed only in living cells) was measured at 550 nm and cell viability was calculated as a percent ratio of the untreated control sample. Data presented are mean values ± standard deviations (n=3). The known-anticancer drug doxorubicin (DOX) used at the concentration of 5 µM served as the positive control. Asterisks denote statistically significant differences in comparison to the untreated control (ctrl) for $p<0.05$ (Student' t-test).

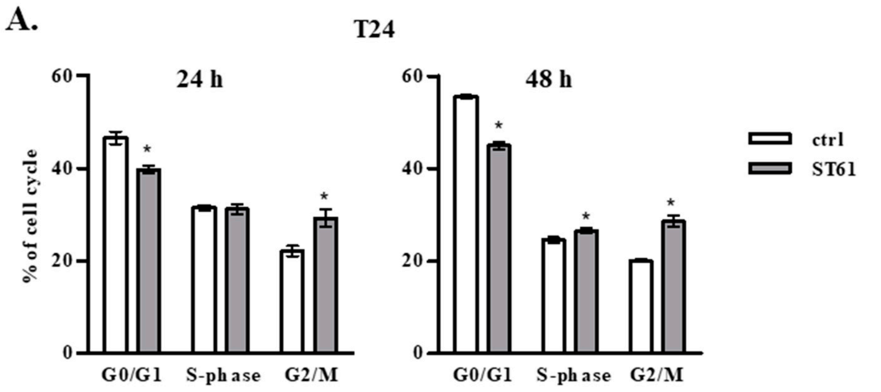

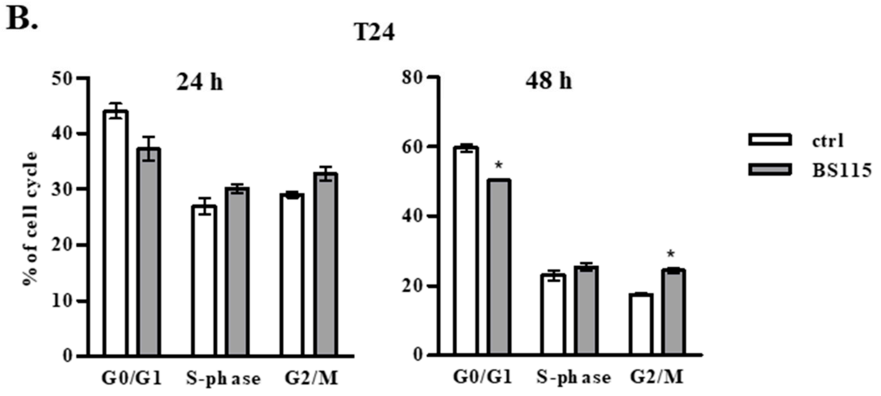

Figure S14. Cell cycle distribution of T24 cancer cells treated with compounds ST61 (A) and BS115 (B). Cells were incubated with the compounds for 24 and 48 h, fixed, stained with propidium iodide and analyzed by flow cytometry. Data presented are mean values ± standard deviations of a representative experiment (n=4, conducted in triplicate). Asterisks represent statistically significant differences in comparison to the respective control (ctrl) for $p<0.05$ (Student' t-test).

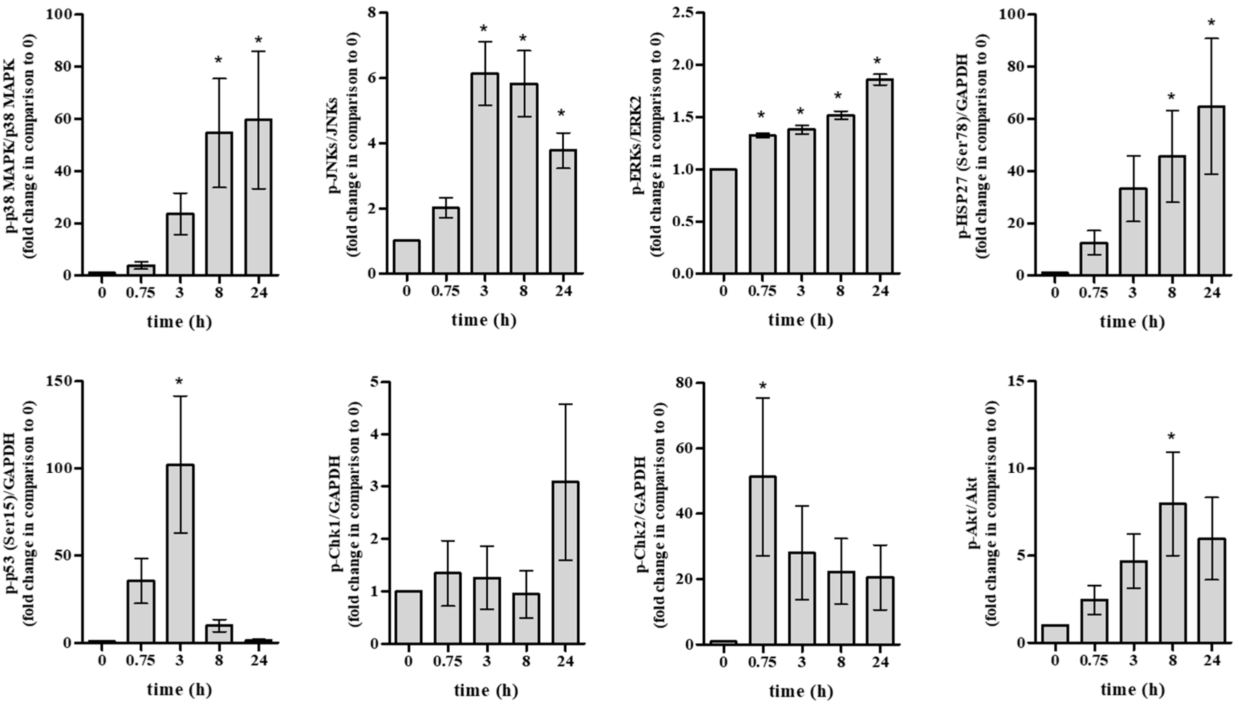

Figure S15. Densitometric analysis from three independent replicates for the quantification of the western blots presented in Figure 5. Asterisks denote statistically significant differences ($p<0.05$) in comparison to t=0. Densitometric analysis was performed using ImageJ.

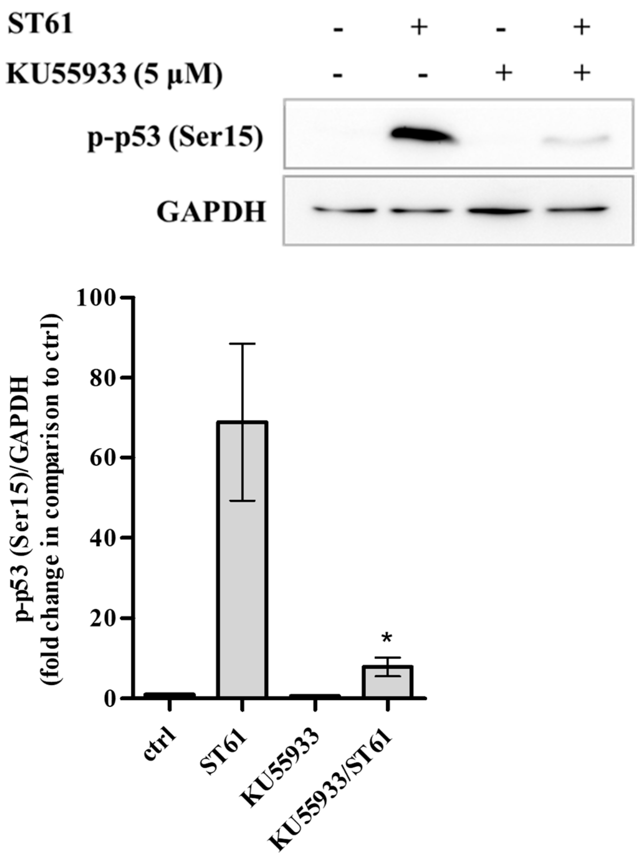

Figure S16. Western blot analysis of protein extracts from ST61-treated MCF7 breast cancer cells for the phosphorylation of p53 after their pre-incubation with the ATM kinase inhibitor, KU55933. MCF7 cells were pre-incubated with 5 μM of KU55933 for 1 h before their exposure to ST61 at its 24-h IC_{50} for 3 h. Western blot analysis was conducted with primary antibodies raised against phospho-p53 (p-p53) on Ser15 and glyceraldehyde 3-phosphate dehydrogenase (GAPDH), the latter used to confirm equal loading among samples. A representative blot from three independent experiments is presented here. Densitometric analysis for the quantification of phosphorylated p53 levels was performed with ImageJ. Asterisk denotes statistical significance ($p<0.05$) in comparison to the respective sample in the absence of KU55933.

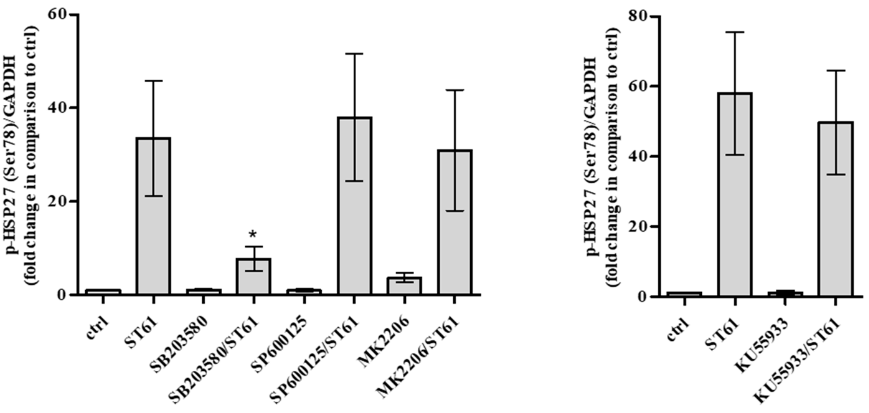

Figure S17. Densitometric analysis from two independent replicates for the quantification of the western blots presented in Figure 7. Asterisk denotes statistical significance in comparison to the respective sample in the absence of any inhibitor. Densitometric analysis was performed using ImageJ.

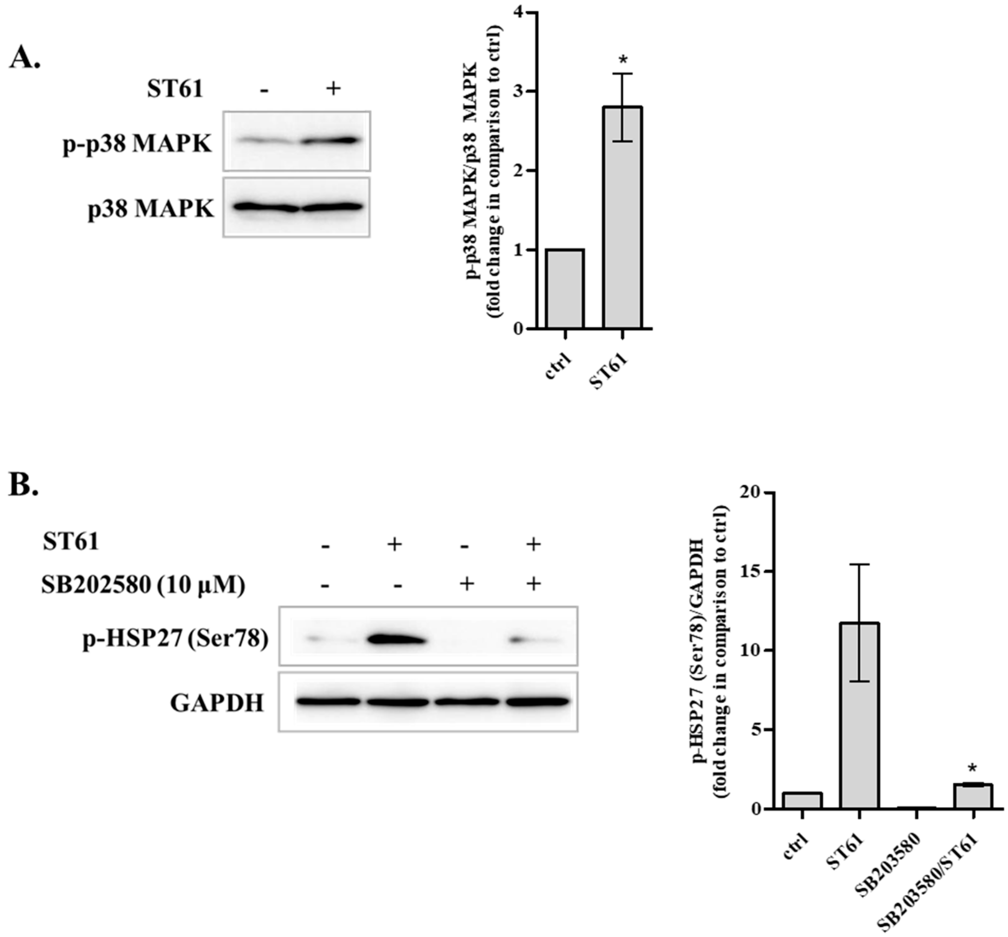

Figure S18. Western blot analysis of protein extracts from ST61-treated A-431 epidermoid cancer cells for the phosphorylation of p38 MAPK, as well as for the phosphorylation of HSP27 in the presence of the p38 MAPK kinase inhibitor, SB203580. A. A-431 cells were incubated with ST61 at its 24-h IC_{50} for 24 h. B. A-431 cells were pre-incubated with 10 μM of SB203580 for 1 h before their exposure to ST61 at its 24-h IC_{50} for 24 h. Western blot analysis was conducted with primary antibodies raised against phospho-p38 MAPK (p-p38 MAPK), p38 MAPK (used to confirm equal loading), phospho-HSP27 on Ser78 (p-HSP27) and glyceraldehyde 3-phosphate dehydrogenase (GAPDH, used to confirm equal loading among samples). Representative blots from two independent experiments are presented here. Densitometric analysis for the quantification of phosphorylated p38 MAPK and HSP27 levels was performed with ImageJ. Asterisk denotes statistical significance ($p<0.05$) in comparison to the untreated ctrl (A) or in comparison to the respective sample in the absence of SB203580 (B).

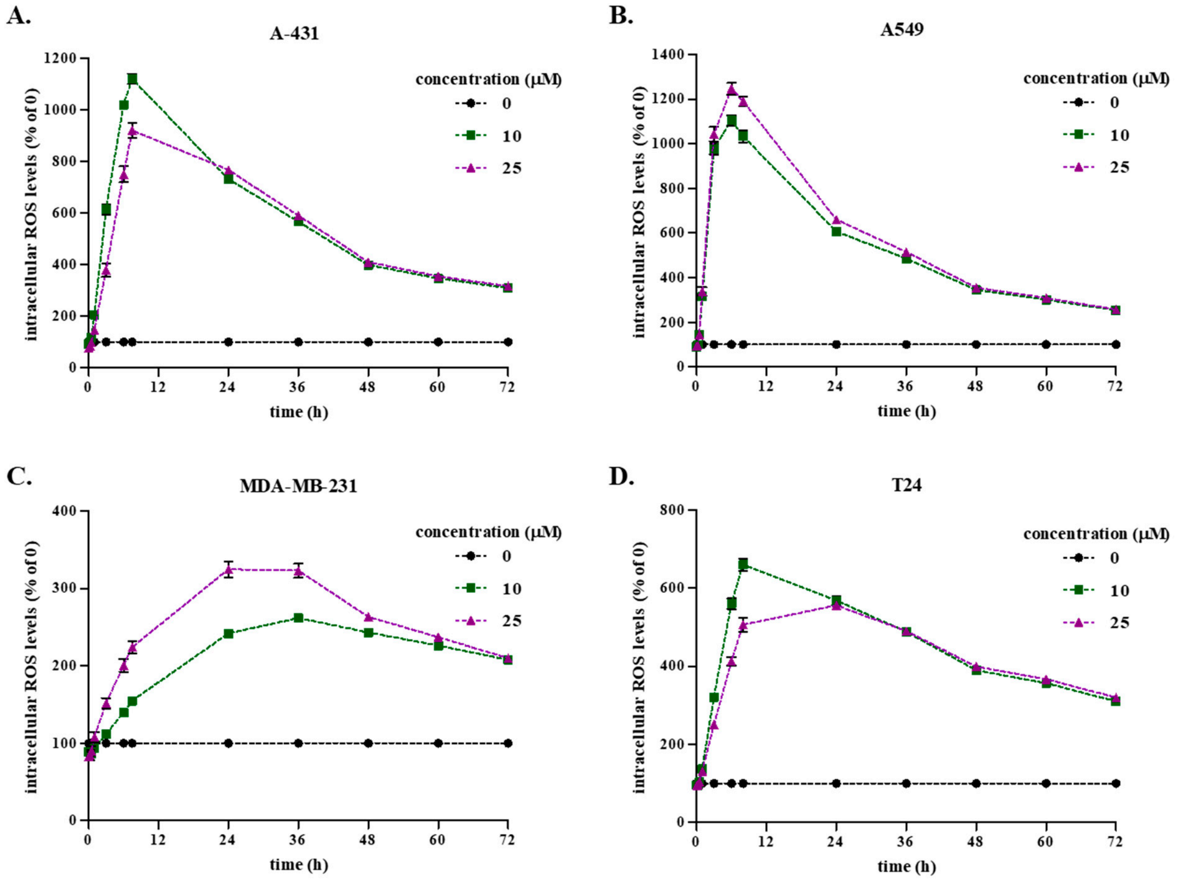

Figure S19. Increase of intracellular ROS levels in ST61-treated A-431 (A), A549 (B), MDA-MB-231 (C) and T24 (D) cancer cells. Cells were incubated with 10 μM of DCFH-DA before the addition of ST61 at concentrations 0, 10 and 25 μM and the estimation of intracellular levels of reactive oxygen species (ROS) by recording fluorescence (excitation wavelength: 485 nm, emission wavelength: 520 nm). ROS production was expressed as a % ratio of the untreated control (0 μM). A representative experiment (n=2, conducted in triplicate) is presented here.

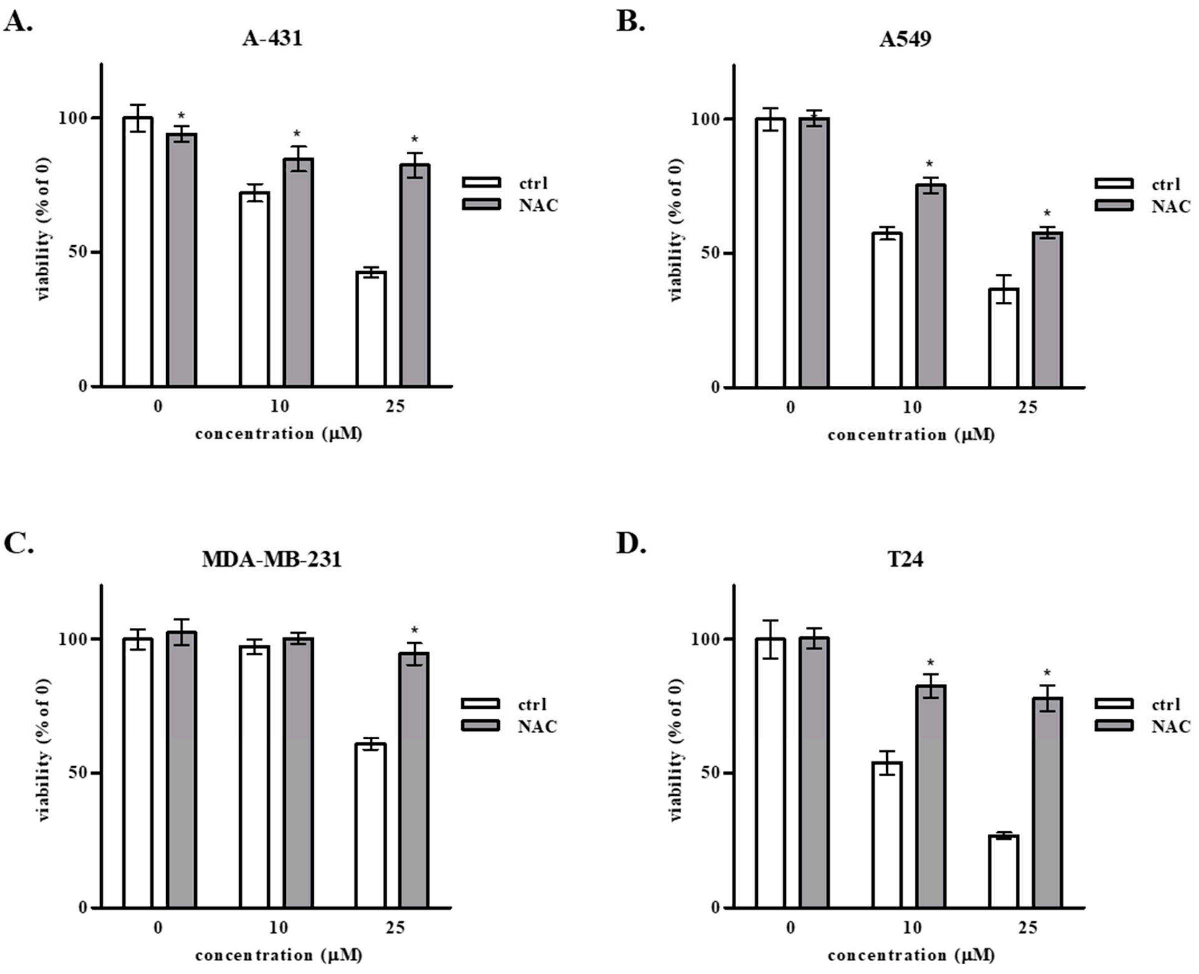

Figure S20. Cytotoxic activity of compound ST61 in the presence of N-acetyl-cysteine (NAC). A-431 (A), A549 (B), MDA-MB-231 (C) and T24 (D) cancer cells were pre-incubated with 2 mM of NAC for 16 h and exposed to 10 and 25 µM of ST61 for 24 h before aspiration of the culture medium and incubation with 1 mg/mL MTT solution for another 4 h at 37°C. Optical density of the solubilized formazan crystals was measured at 550 nm and cell viability was calculated as a percent ratio of the untreated control sample. Data presented are mean values ± standard deviations from two independent experiments. Asterisks denote statistically significant differences in comparison to the respective sample without NAC (Student' t-test, * $p<0.05$).

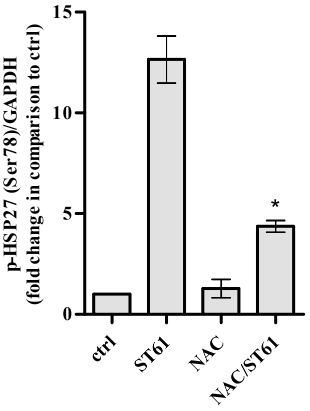

Figure S21. Densitometric analysis from two independent replicates for the quantification of the western blot presented in Figure 9. Asterisk denotes statistical significance ($p<0.05$) in comparison to the respective sample in the absence of NAC. Densitometric analysis was performed using ImageJ.

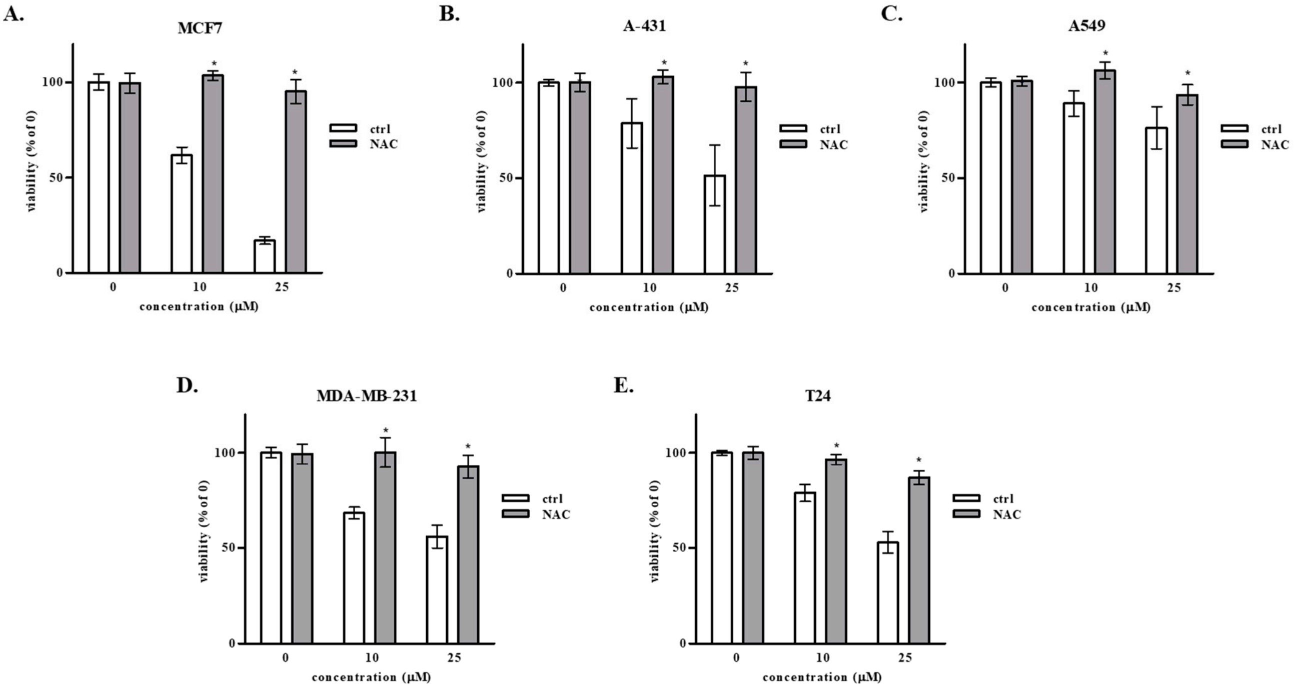

Figure S22. Cytotoxic activity of compound ST61 in the presence of N-acetyl-cysteine (NAC), as estimated by the Neutral Red uptake assay. MCF7 (A), A-431 (B), A549 (C), MDA-MB-231 (D) and T24 (E) cancer cells were pre-incubated with 2 mM of NAC for 16 h and exposed to 10 and 25 μM of ST61 for 24 h before aspiration of the culture medium and incubation with 20 μg/mL neutral red for another 4 h at 37°C. Neutral red was extracted using an acidified ethanol solution. Fluorescence was measured using excitation and emission wavelengths of 530 and 645 nm, respectively and cell viability was expressed as a % ratio of untreated cells. Data presented are mean values ± standard deviations from two independent experiments. Asterisks denote statistically significant differences in comparison to the respective sample without NAC (Student' t-test, * $p<0.05$).

Table S1. Chemical structures of selected compounds displaying anti-cancer activity in ≥ 5 of the tested cancer cell lines.

Compound	Chemical structure
8A	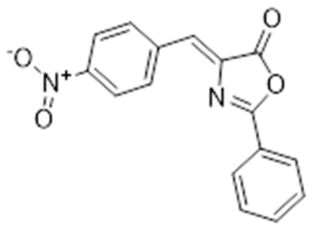 [1]
24A	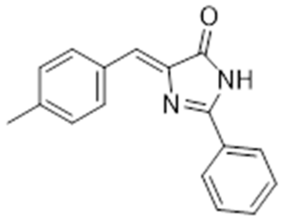 [1]
25A	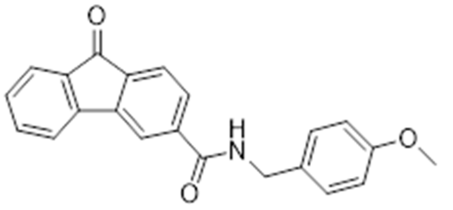 [2]
37A	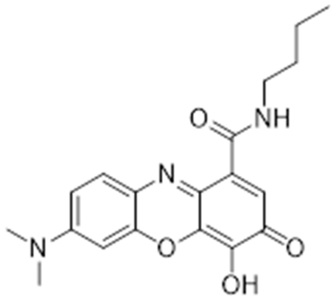 BS125
42A	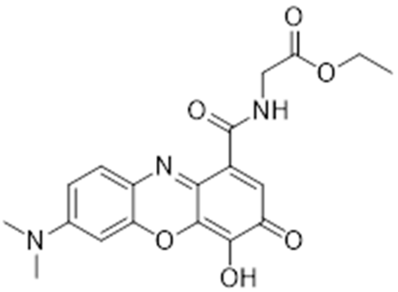 BS115

11B	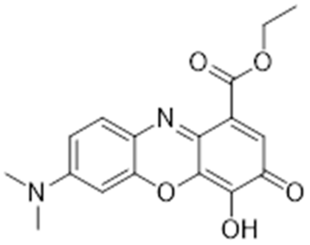 BS74
12B	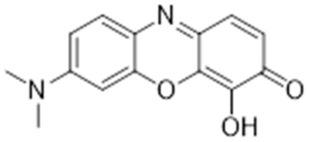 ST61
22B	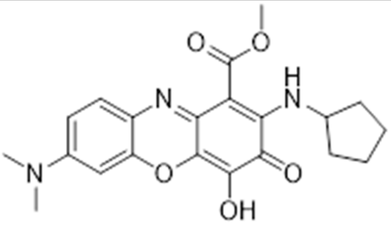 [3]
44B	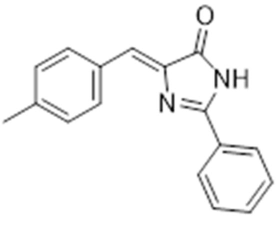 [1]

Table S2. Calculated ST61 72-h IC_{50} values for MCF7, MDA-MB-231, A-431 and A549 cancer cells, as well as for normal human skin and breast fibroblasts.

cell line	72-h IC_{50} in µM (± stdev)
MCF7	2.8 ± 0.4
MDA-MB-231	5.9 ± 0.2
A-431	3.4 ± 0.9
A549	3.1 ± 1.4
normal skin fibroblasts	21.8 ± 0.3
normal breast fibroblasts	24.9 ± 8.1
