# Supplementary figures and images for: Phenoxazine Derivative ST61 Displays an Oxidative-Stress-Mediated Cytotoxic Activity Against Human Cancer Cells
Source: Biomolecules. 2026 May 6;16(5):689. doi: 10.3390/biom16050689 (PMC13204190; doi:10.3390/biom16050689)

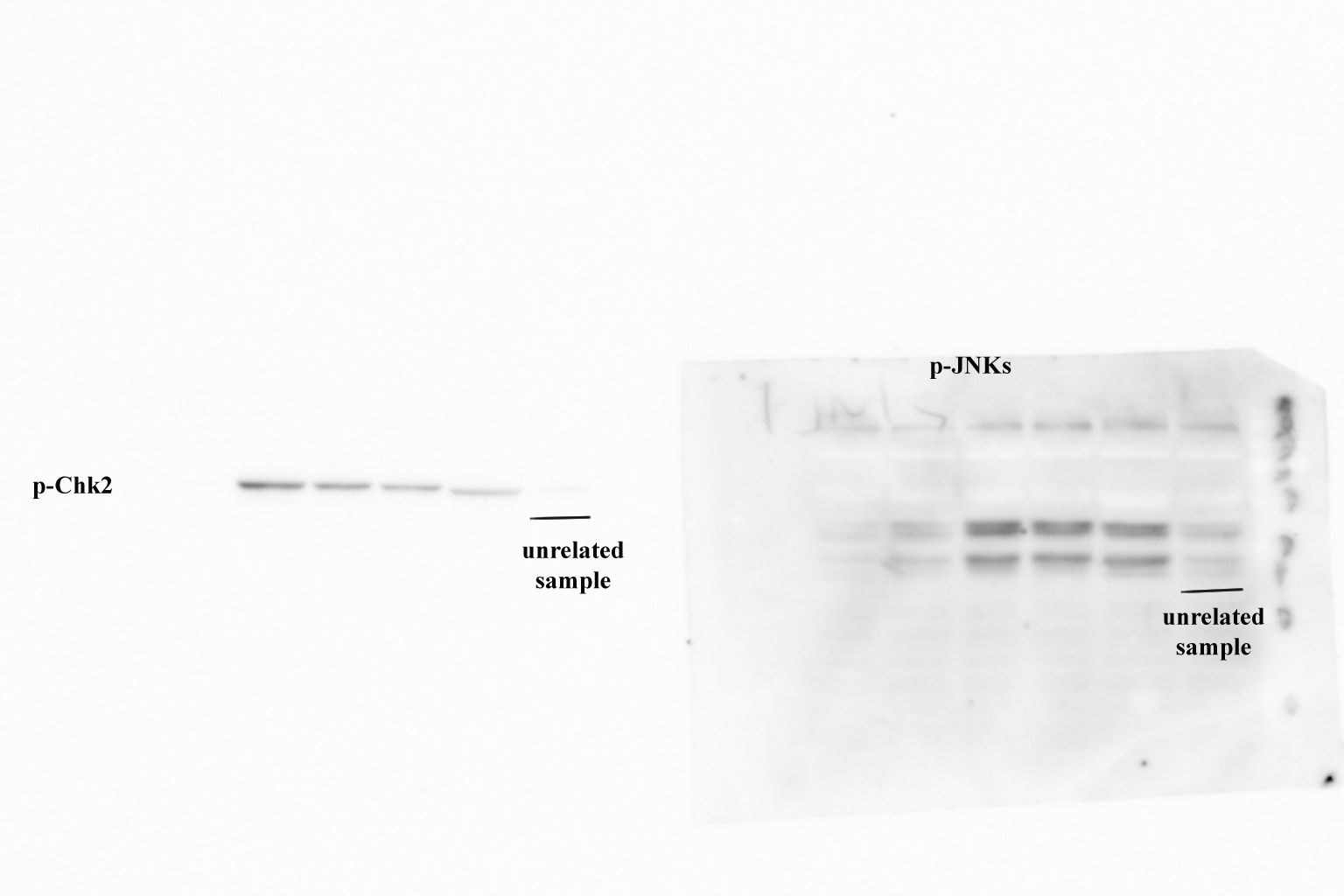

Supplement: Supplementary file 1 [file biomolecules-16-00689-s001.zip › File S1. original WB images/biomolecules-4275260_Original blots/Figure 5B and C, pChk2 and pJNKs/Figure 5B and C_pChk2 and pJNKs.tif]

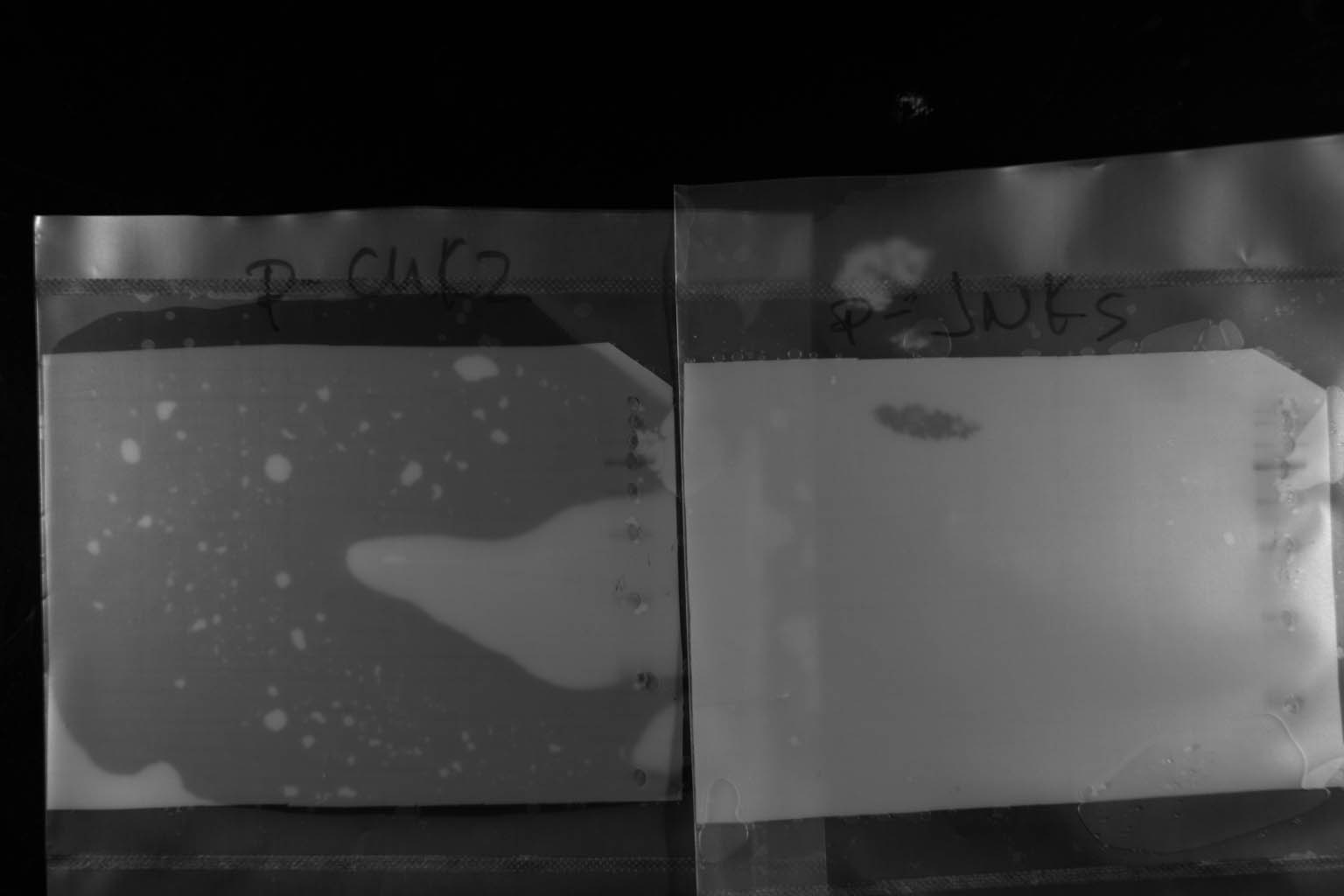

Supplement: Supplementary file 1 [file biomolecules-16-00689-s001.zip › File S1. original WB images/biomolecules-4275260_Original blots/Figure 5B and C, pChk2 and pJNKs/markers.jpg]

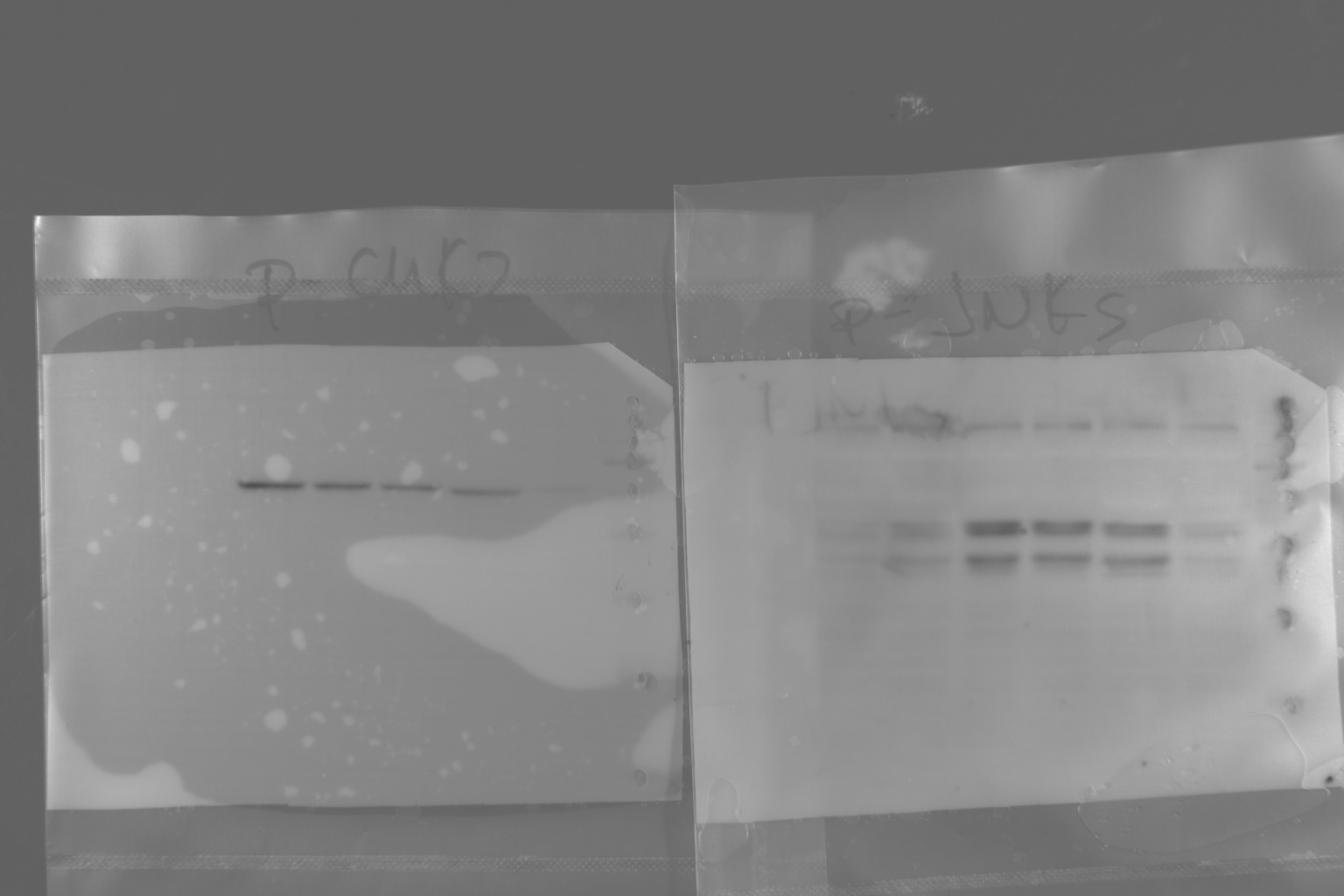

Supplement: Supplementary file 1 [file biomolecules-16-00689-s001.zip › File S1. original WB images/biomolecules-4275260_Original blots/Figure 5B and C, pChk2 and pJNKs/pChk2 and pJNKs overaly with markers.tif]

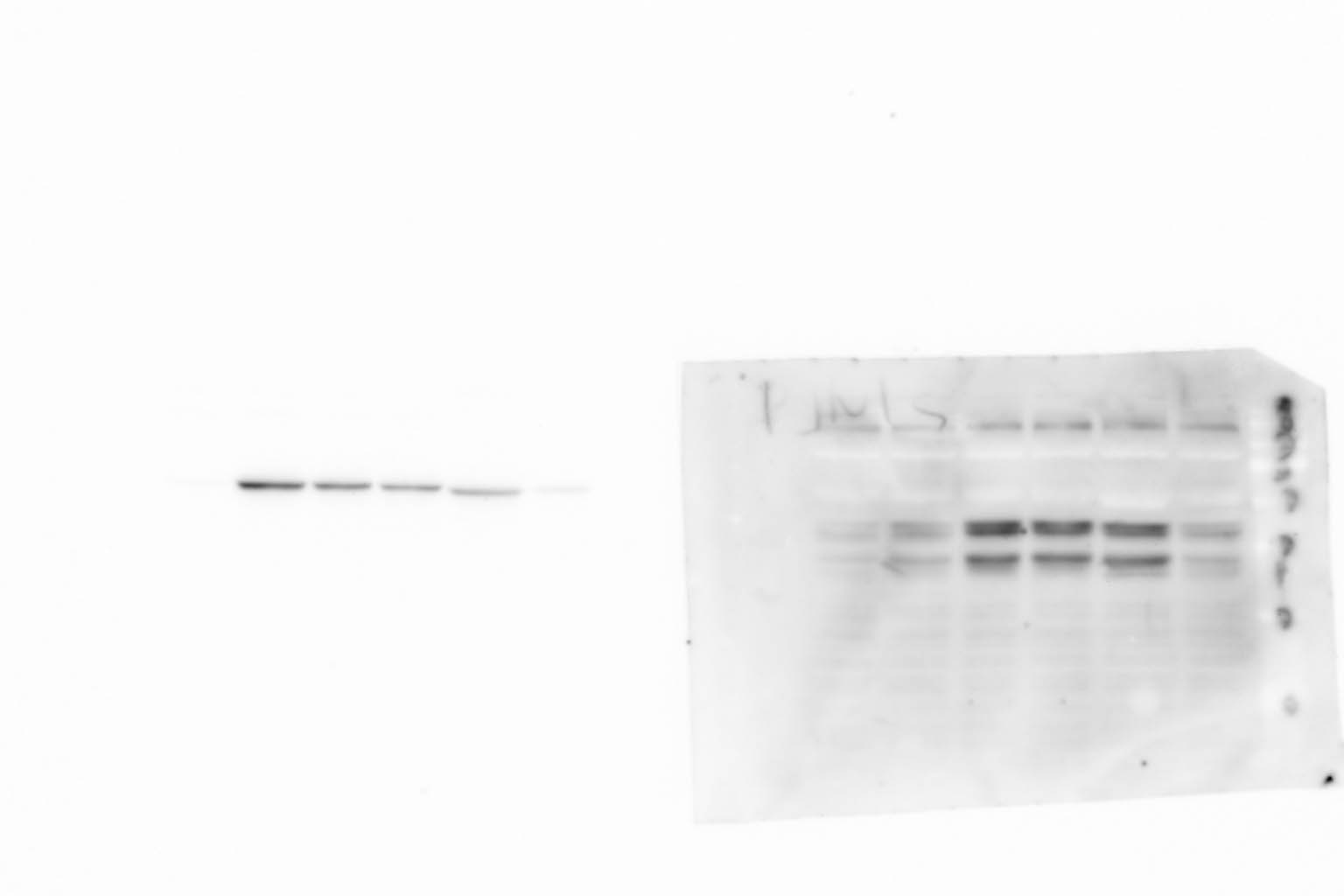

Supplement: Supplementary file 1 [file biomolecules-16-00689-s001.zip › File S1. original WB images/biomolecules-4275260_Original blots/Figure 5B and C, pChk2 and pJNKs/pChk2 and pJNKs.jpg]

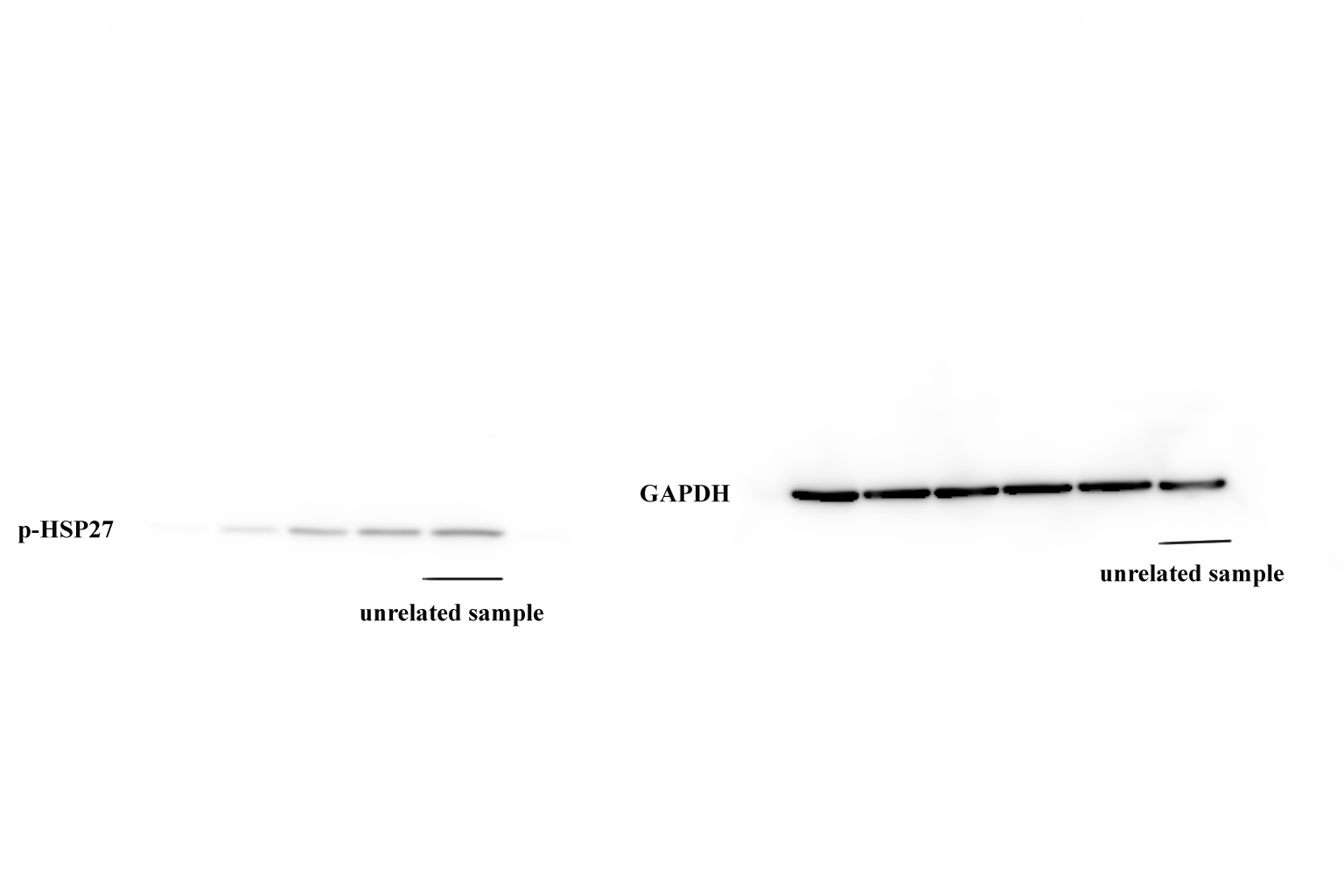

Supplement: Supplementary file 1 [file biomolecules-16-00689-s001.zip › File S1. original WB images/biomolecules-4275260_Original blots/Figure 5B and C, pHSP27 and GAPDH/Figure 5B and C_pHSP27 and GAPDH.tif]

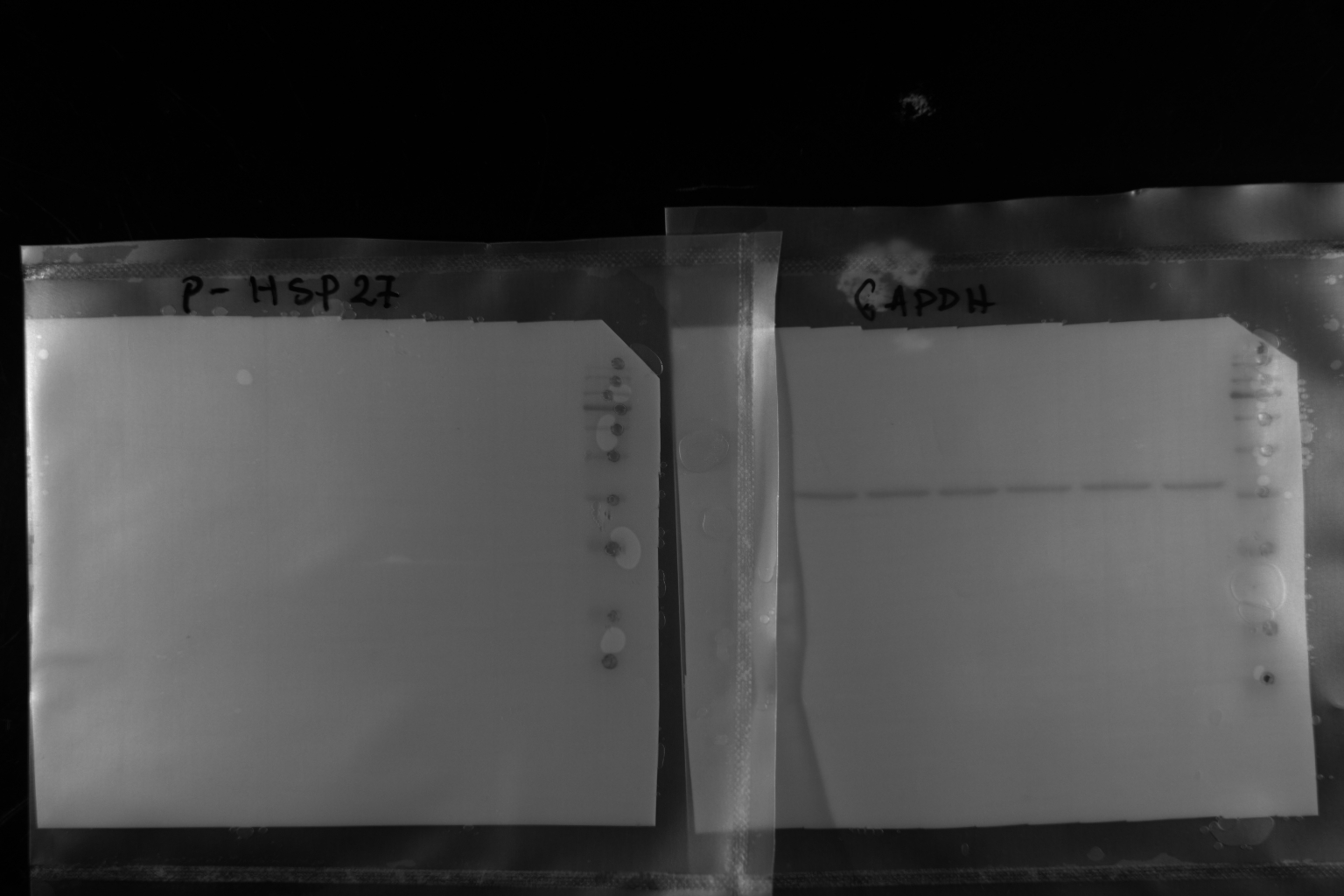

Supplement: Supplementary file 1 [file biomolecules-16-00689-s001.zip › File S1. original WB images/biomolecules-4275260_Original blots/Figure 5B and C, pHSP27 and GAPDH/markers pHSP27 and GAPDH.jpg]

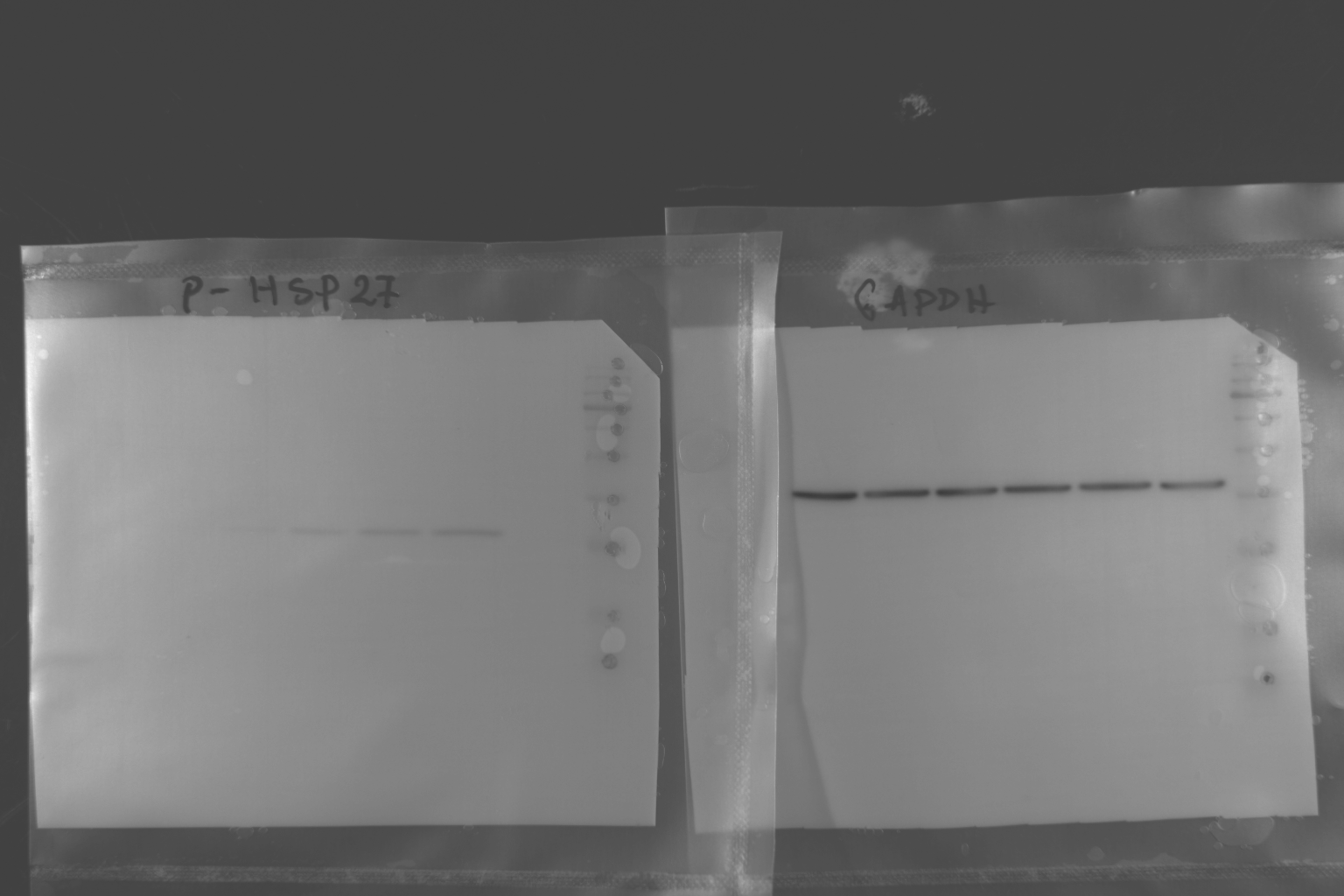

Supplement: Supplementary file 1 [file biomolecules-16-00689-s001.zip › File S1. original WB images/biomolecules-4275260_Original blots/Figure 5B and C, pHSP27 and GAPDH/pHSP27 and GAPDH overlay with markers.tif]

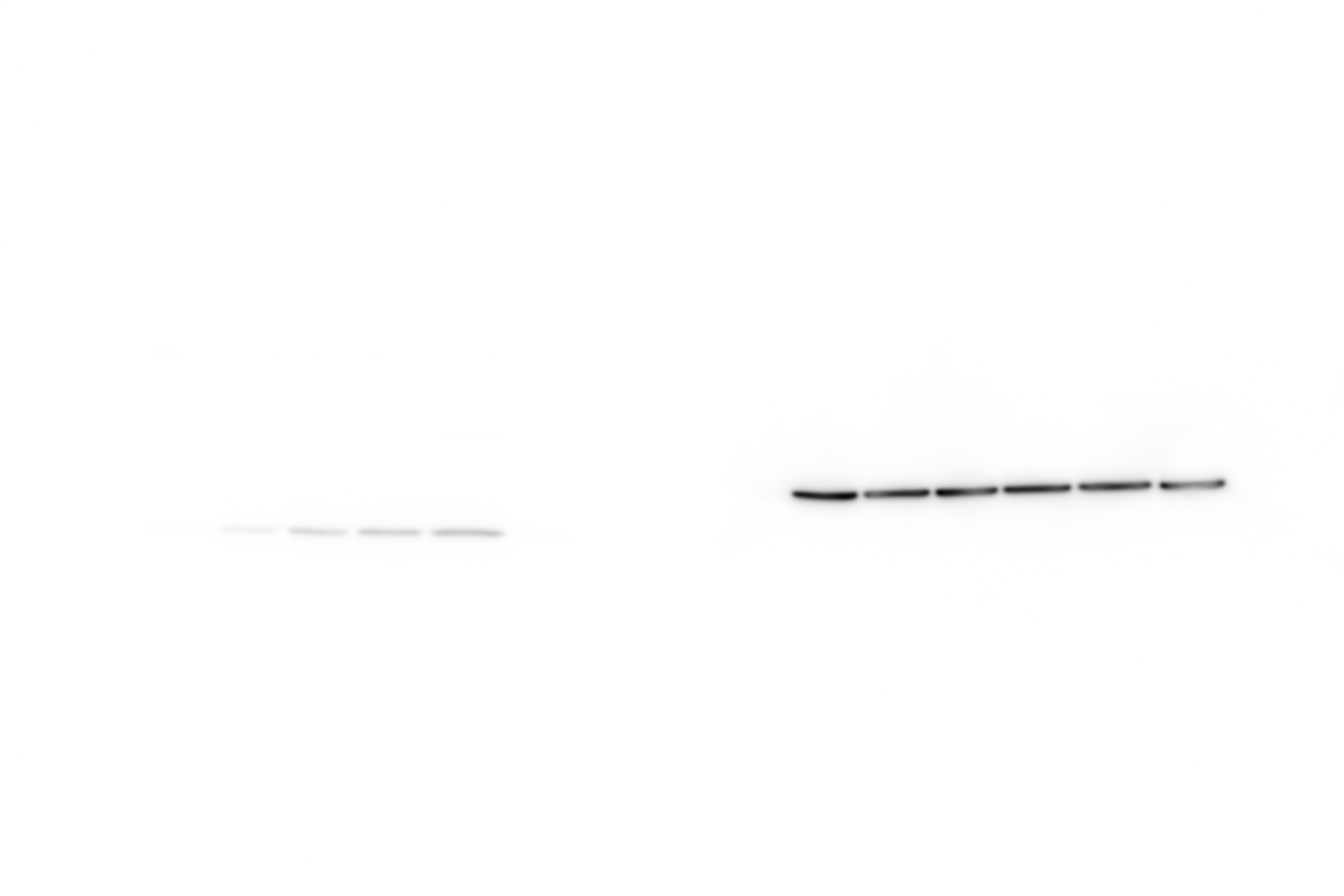

Supplement: Supplementary file 1 [file biomolecules-16-00689-s001.zip › File S1. original WB images/biomolecules-4275260_Original blots/Figure 5B and C, pHSP27 and GAPDH/pHSP27 and GAPDH.jpg]

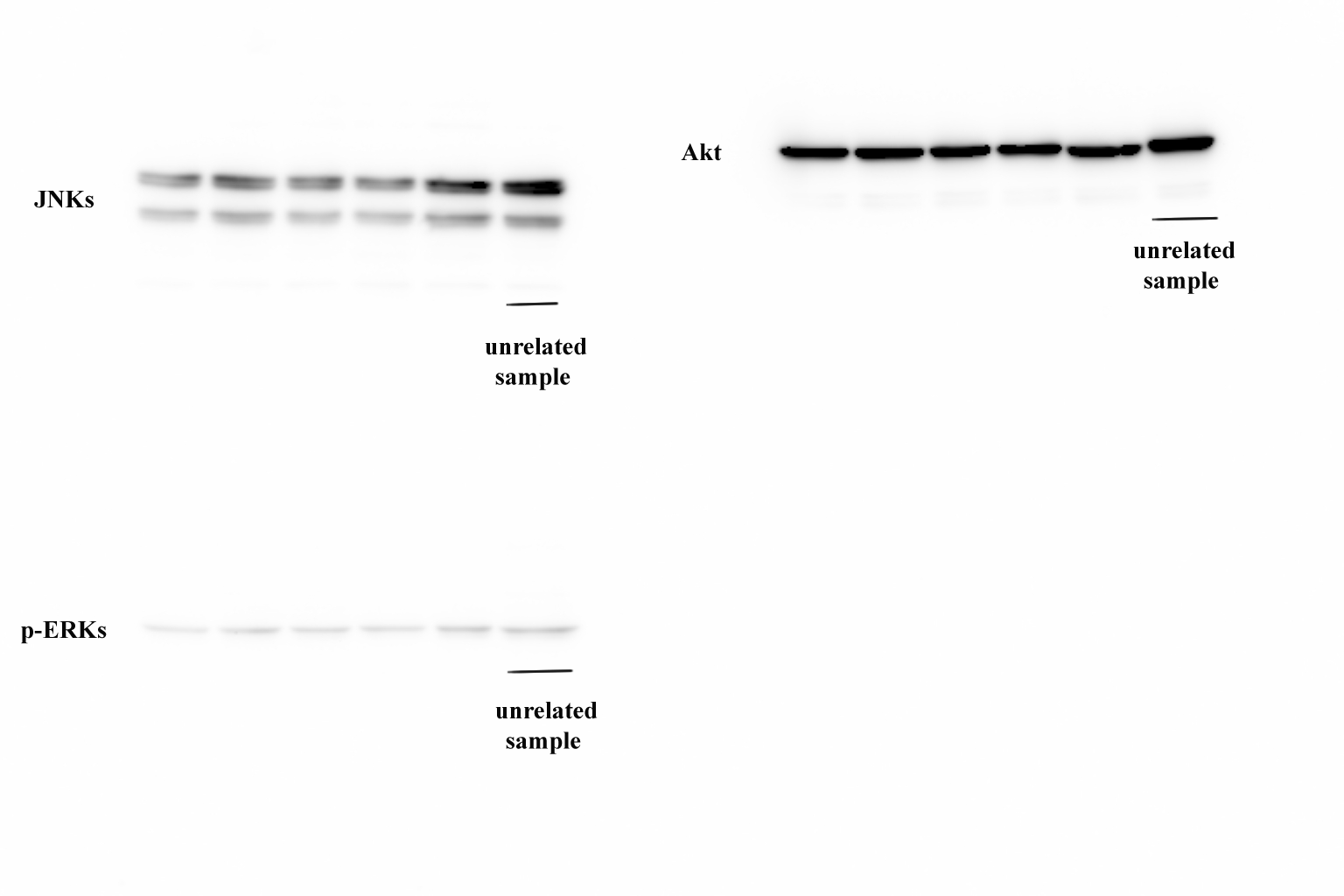

Supplement: Supplementary file 1 [file biomolecules-16-00689-s001.zip › File S1. original WB images/biomolecules-4275260_Original blots/Figure 5B and D, JNKs, pERKs and Akt/Figure 5B and C_JNKs, p-ERKs and Akt.tif]

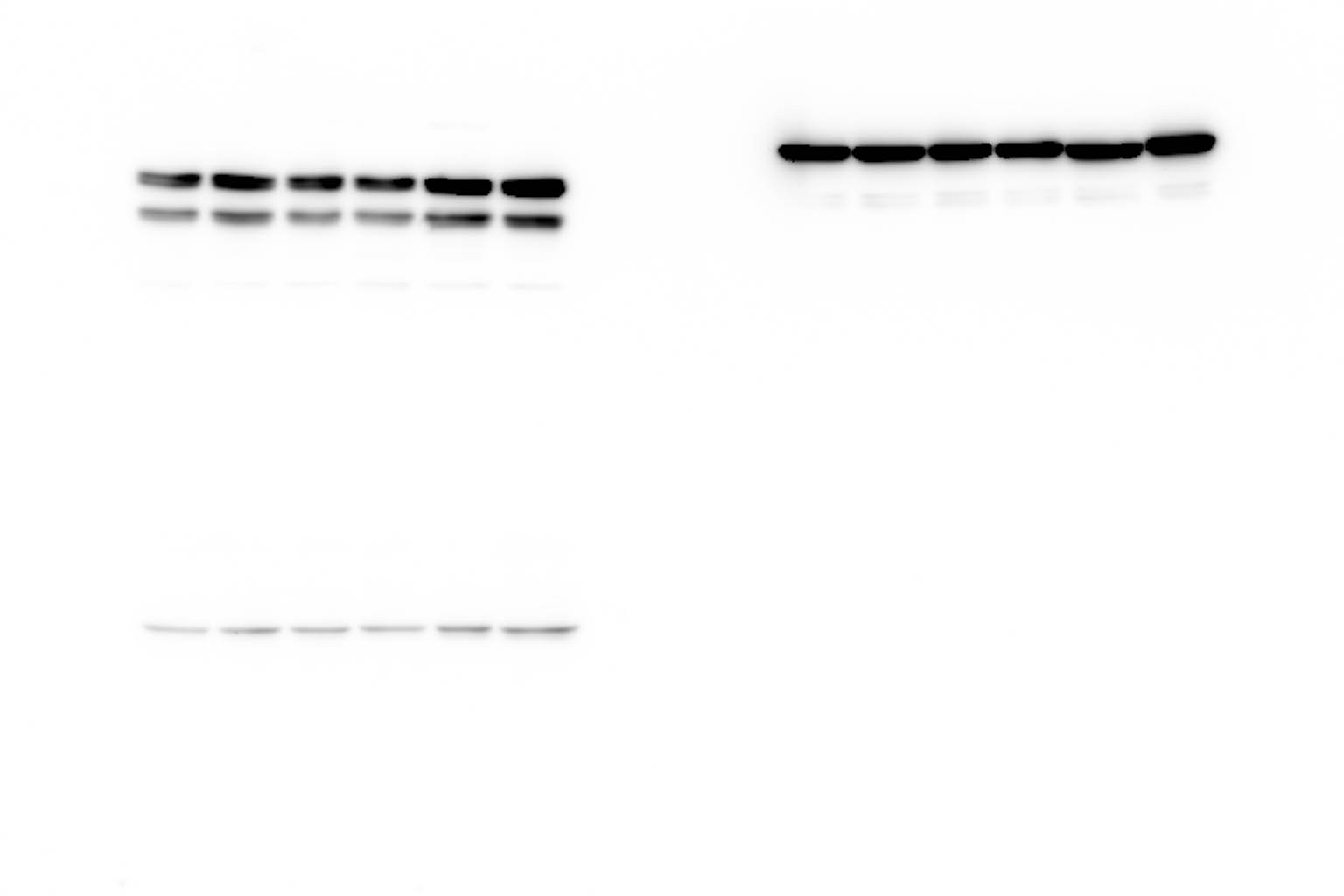

Supplement: Supplementary file 1 [file biomolecules-16-00689-s001.zip › File S1. original WB images/biomolecules-4275260_Original blots/Figure 5B and D, JNKs, pERKs and Akt/JNKs, pERK and Akt.jpg]

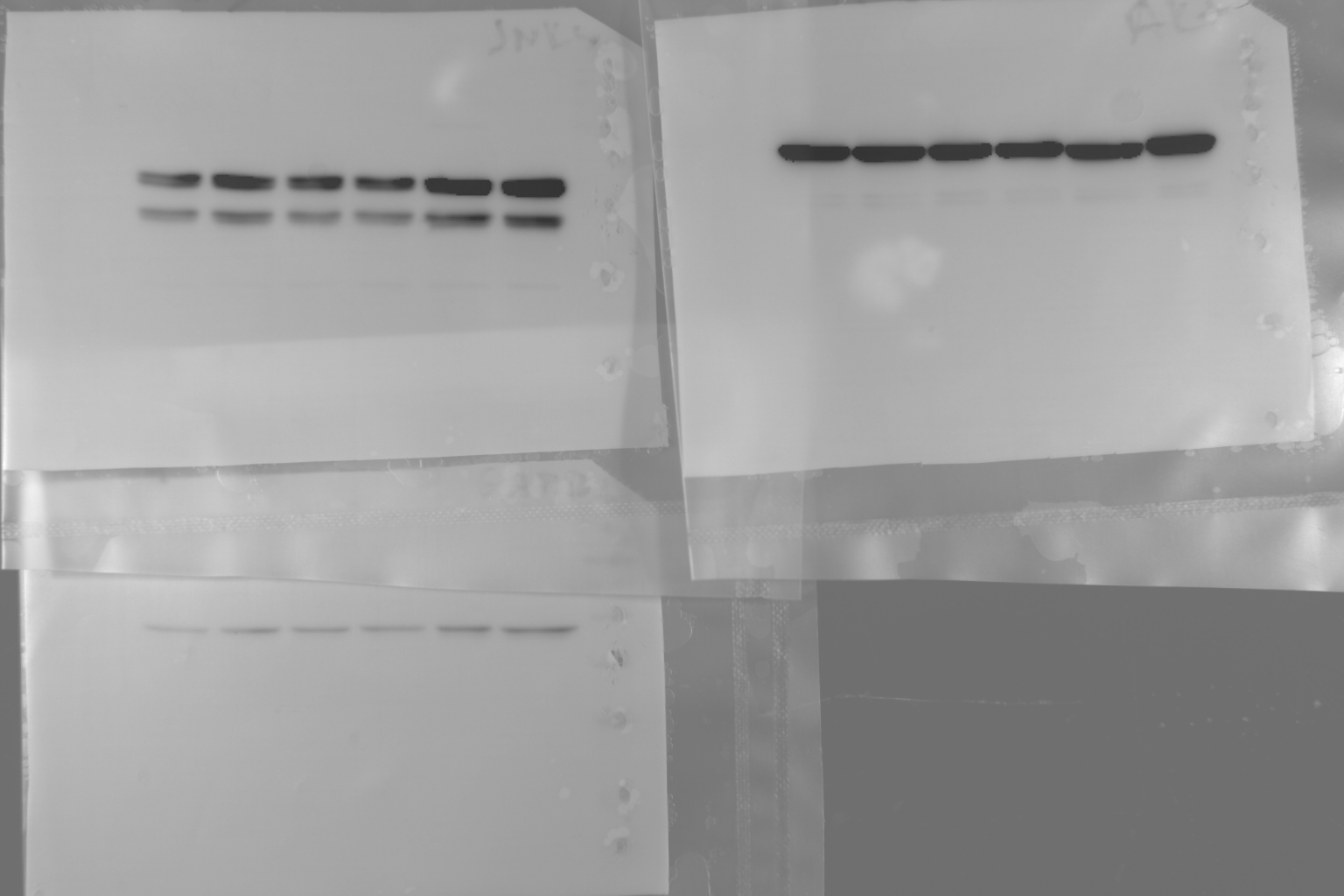

Supplement: Supplementary file 1 [file biomolecules-16-00689-s001.zip › File S1. original WB images/biomolecules-4275260_Original blots/Figure 5B and D, JNKs, pERKs and Akt/JNKs, pERks and Akt overlay with markers.tif]

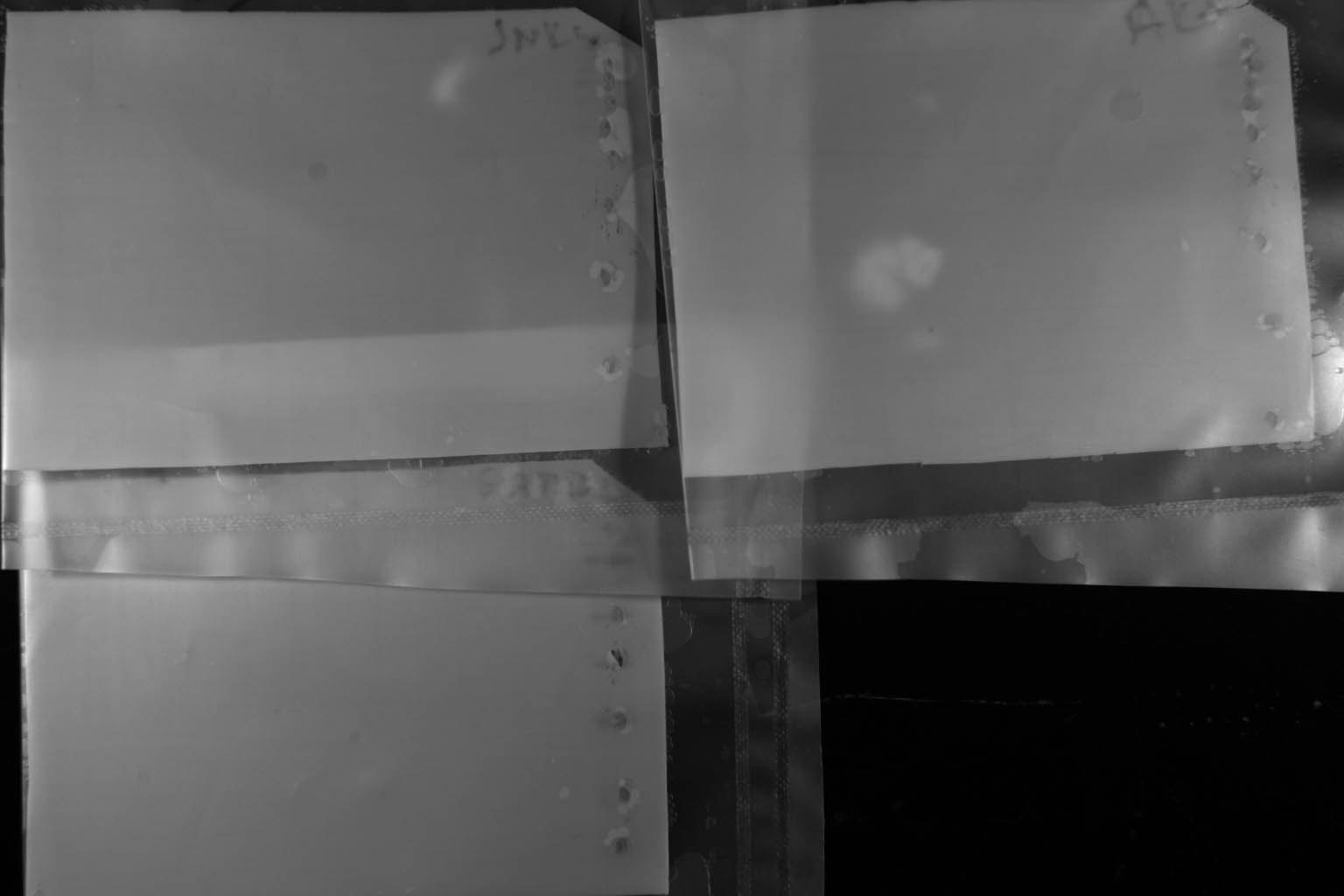

Supplement: Supplementary file 1 [file biomolecules-16-00689-s001.zip › File S1. original WB images/biomolecules-4275260_Original blots/Figure 5B and D, JNKs, pERKs and Akt/markers.jpg]

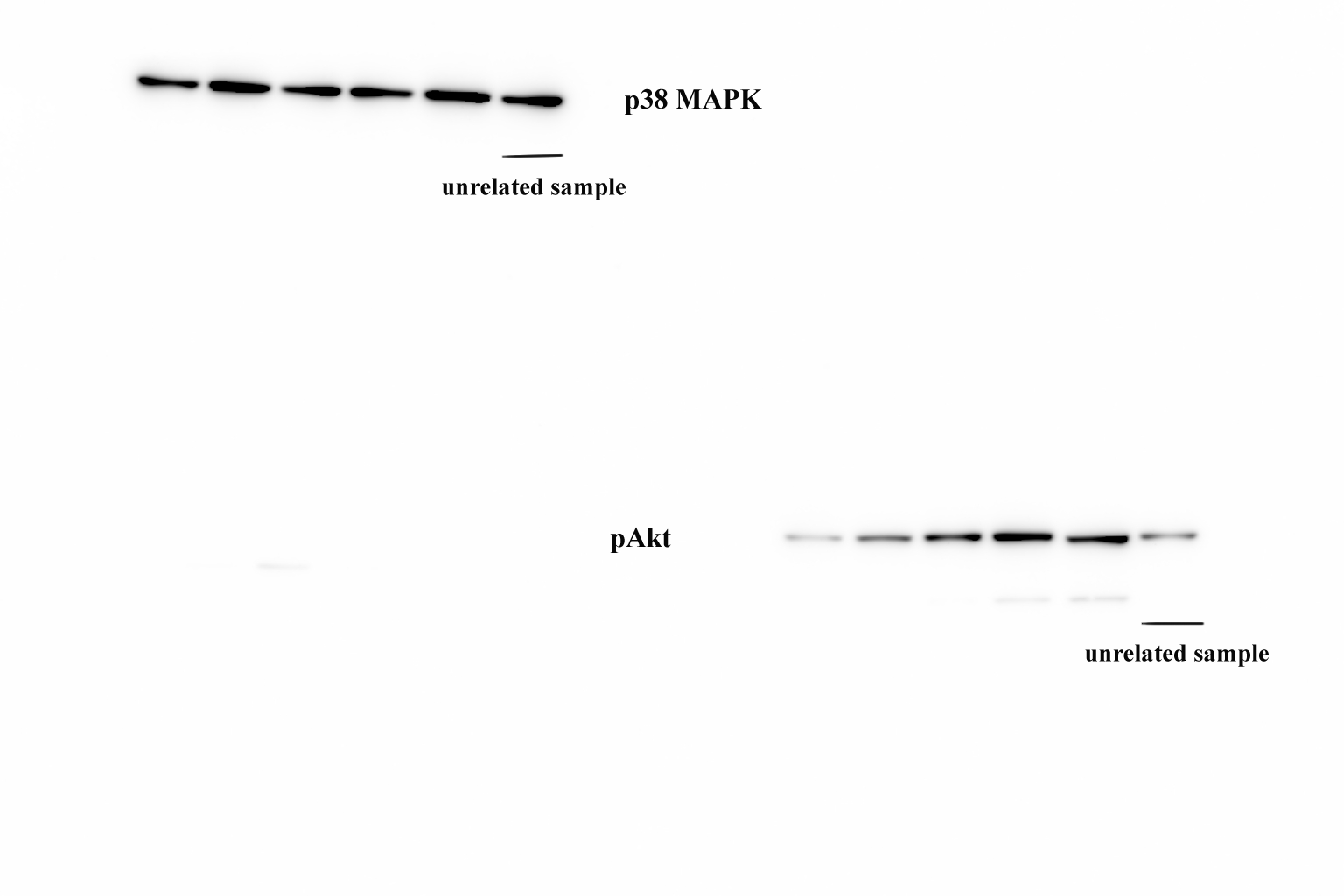

Supplement: Supplementary file 1 [file biomolecules-16-00689-s001.zip › File S1. original WB images/biomolecules-4275260_Original blots/Figure 5B and D, p38 MAPK and pAkt/Figure 5B and D_p38 MAPK and pAkt.tif]

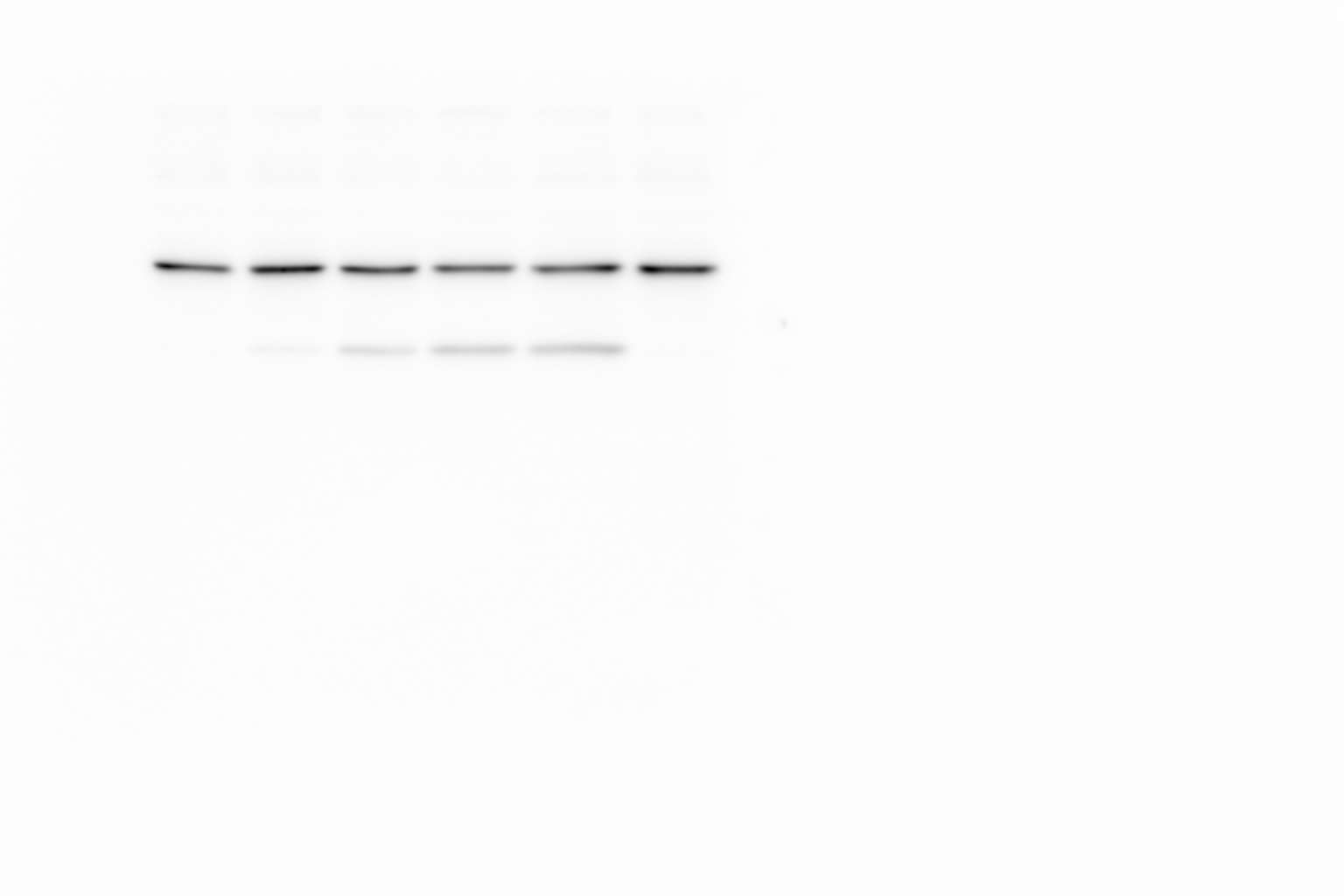

Supplement: Supplementary file 1 [file biomolecules-16-00689-s001.zip › File S1. original WB images/biomolecules-4275260_Original blots/Figure 5B, ERK2/ERK2.tif]

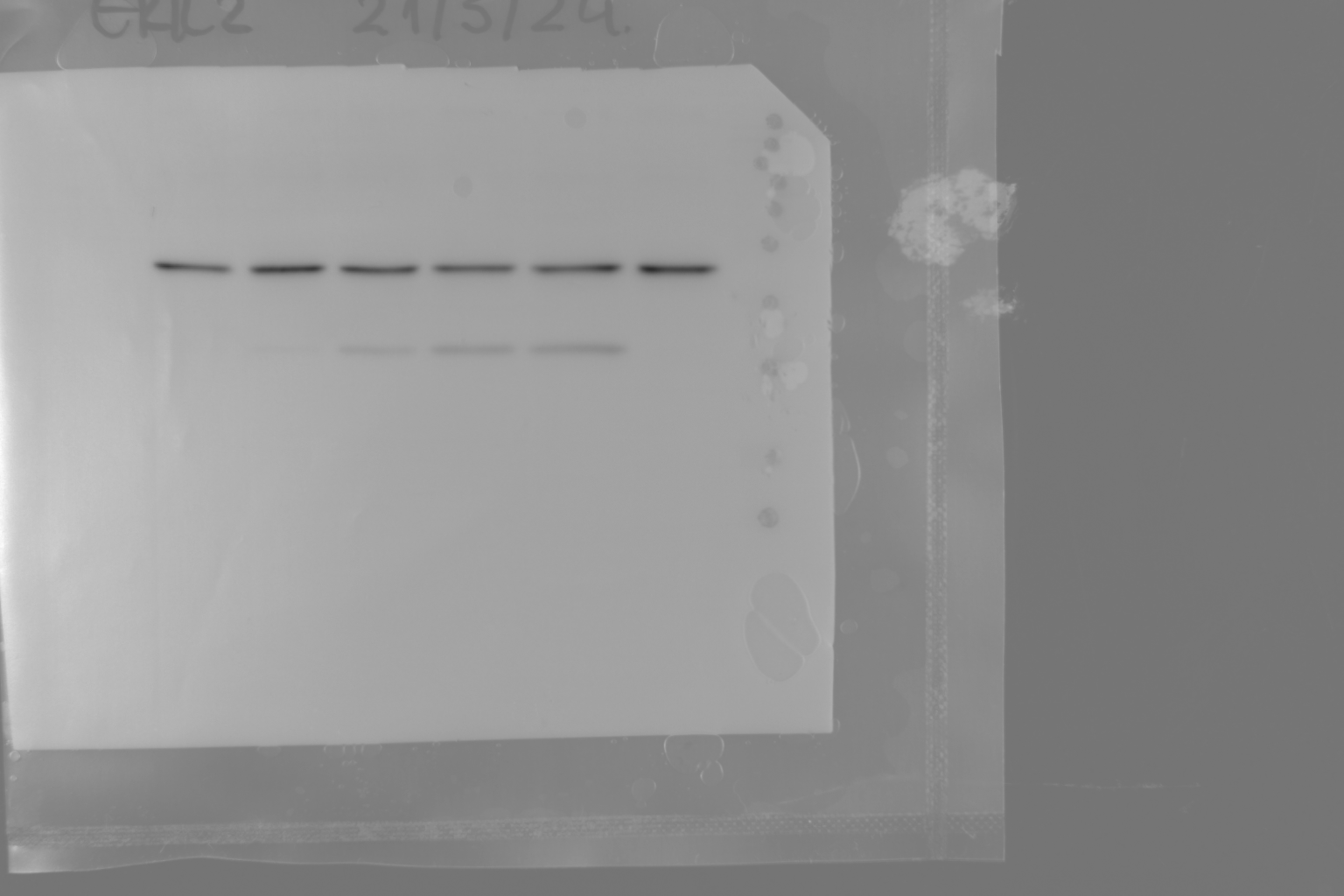

Supplement: Supplementary file 1 [file biomolecules-16-00689-s001.zip › File S1. original WB images/biomolecules-4275260_Original blots/Figure 5B, ERK2/Figure 5B, ERK2 overlay with markers.tif]

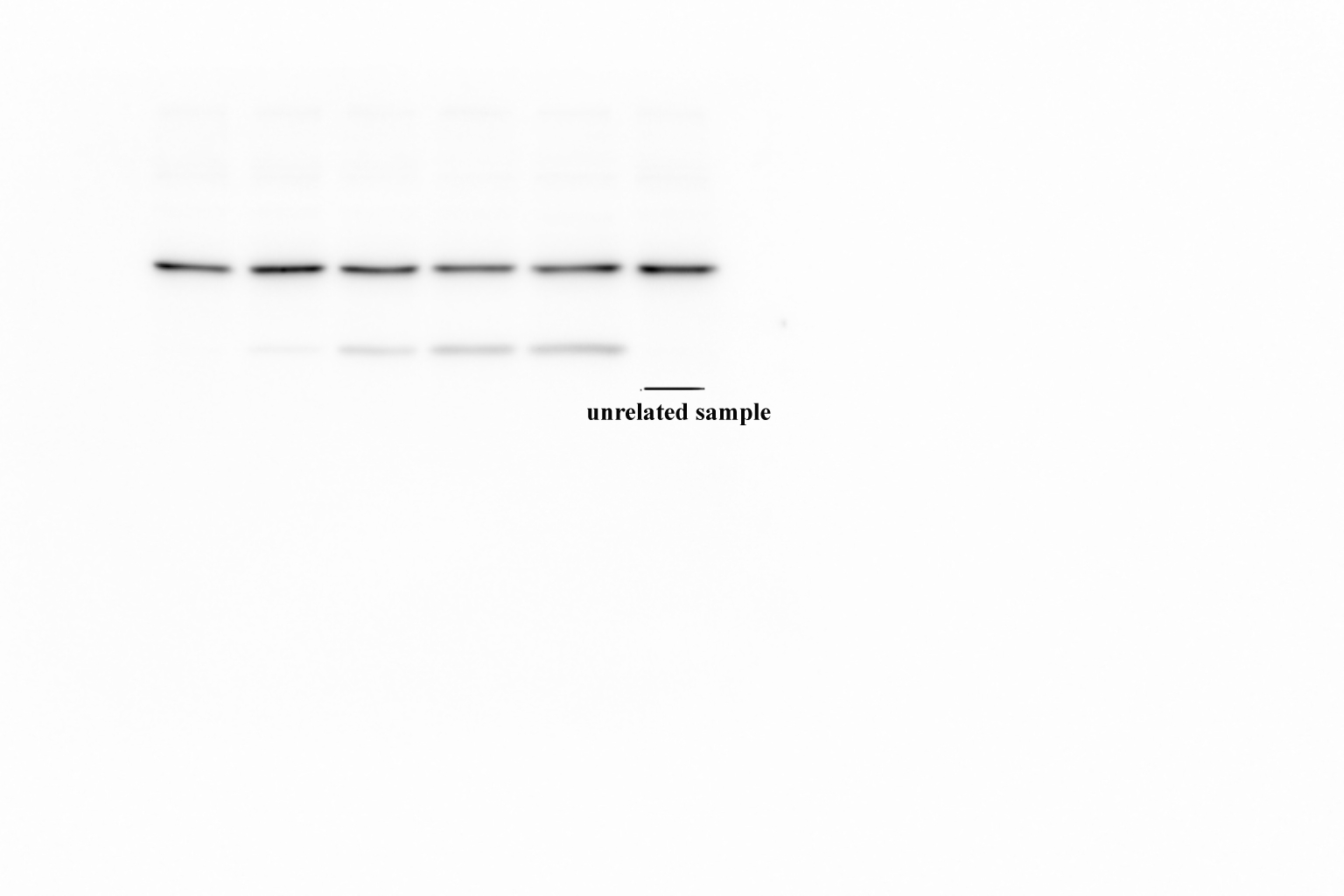

Supplement: Supplementary file 1 [file biomolecules-16-00689-s001.zip › File S1. original WB images/biomolecules-4275260_Original blots/Figure 5B, ERK2/Figure 5B_ERK2.tif]

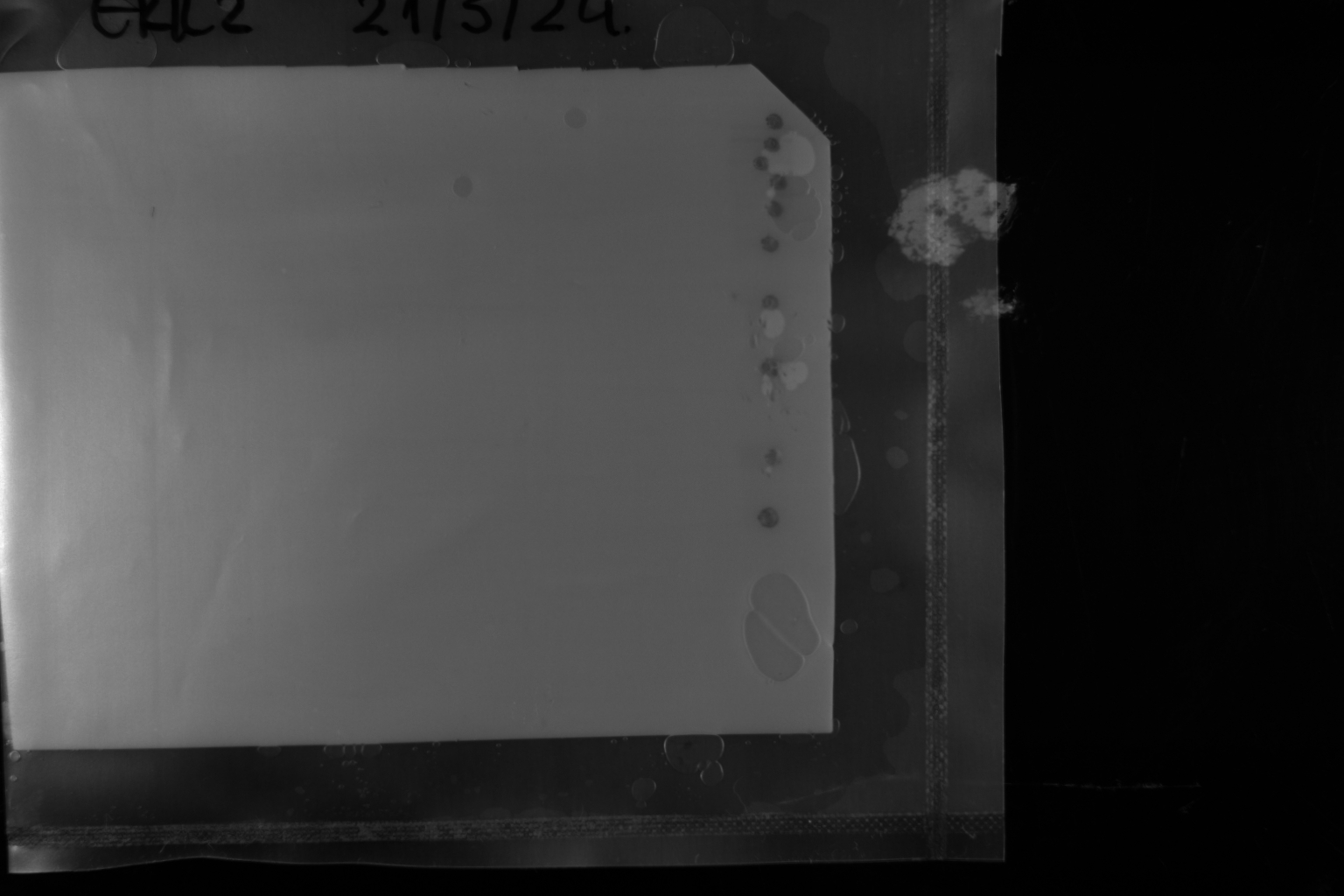

Supplement: Supplementary file 1 [file biomolecules-16-00689-s001.zip › File S1. original WB images/biomolecules-4275260_Original blots/Figure 5B, ERK2/markers.tif]

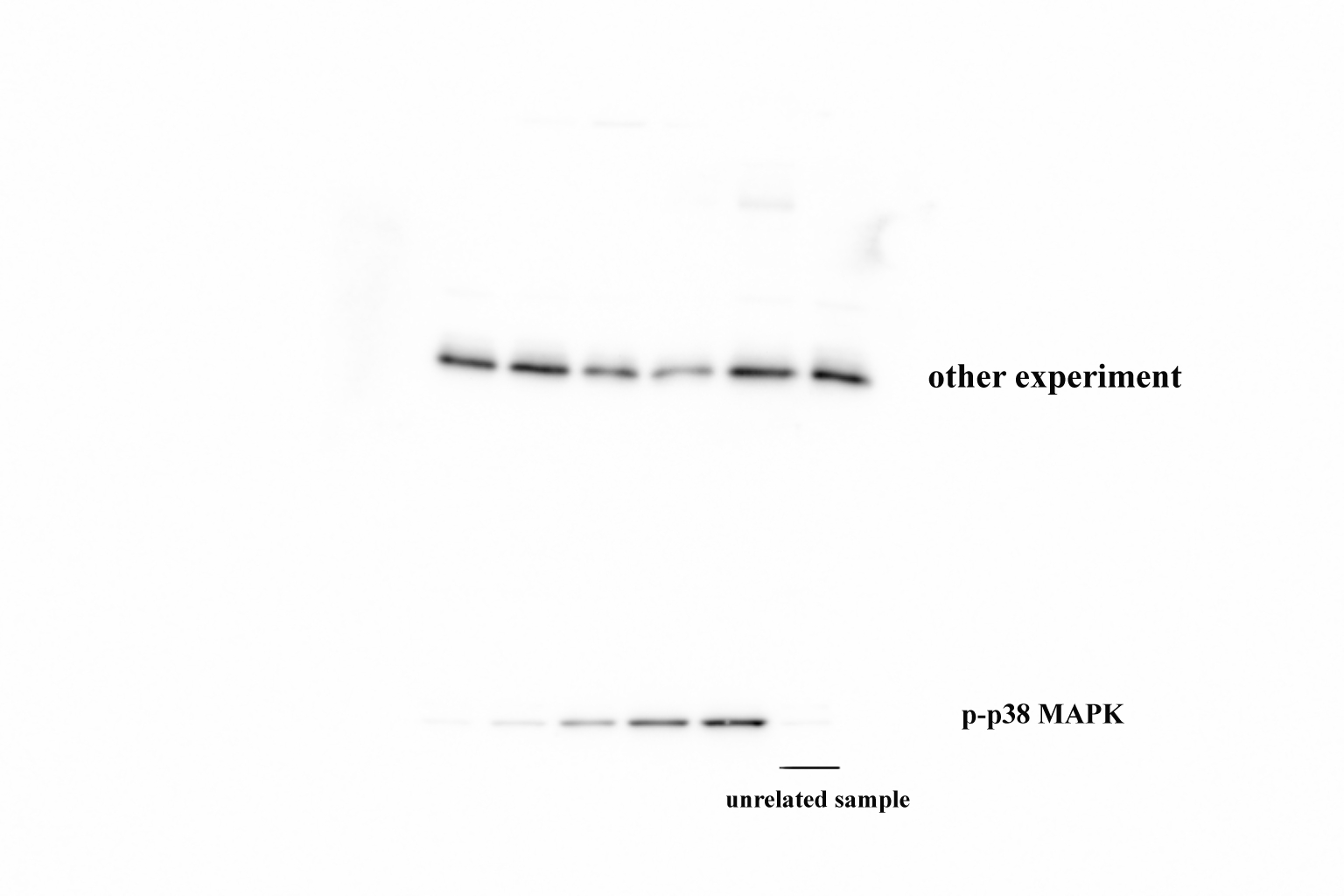

Supplement: Supplementary file 1 [file biomolecules-16-00689-s001.zip › File S1. original WB images/biomolecules-4275260_Original blots/Figure 5B, pp38 MAPK/Figure 5B_pp38 MAPK.tif]

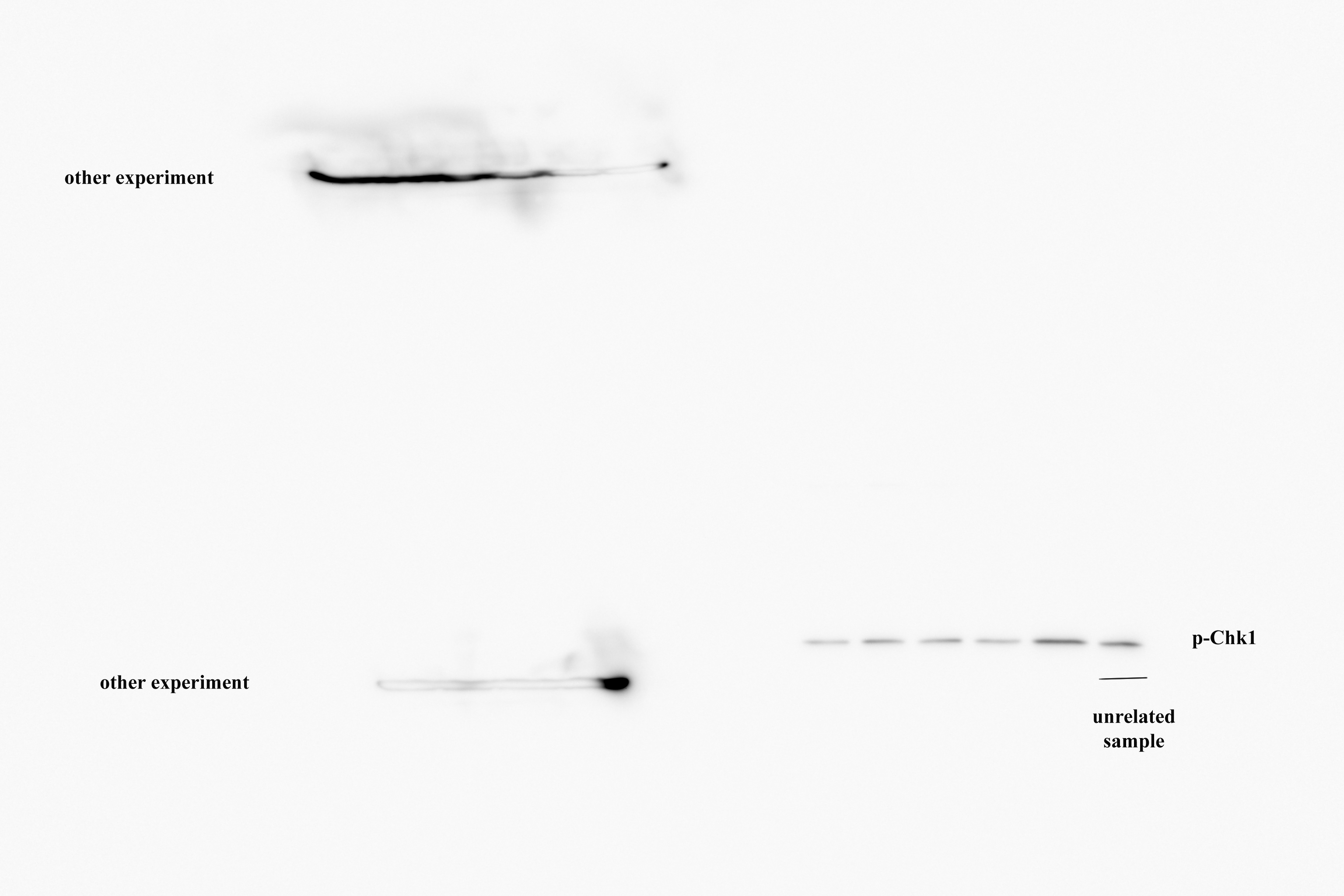

Supplement: Supplementary file 1 [file biomolecules-16-00689-s001.zip › File S1. original WB images/biomolecules-4275260_Original blots/Figure 5C, pChk1/Figure 5C_pChk1.tif]

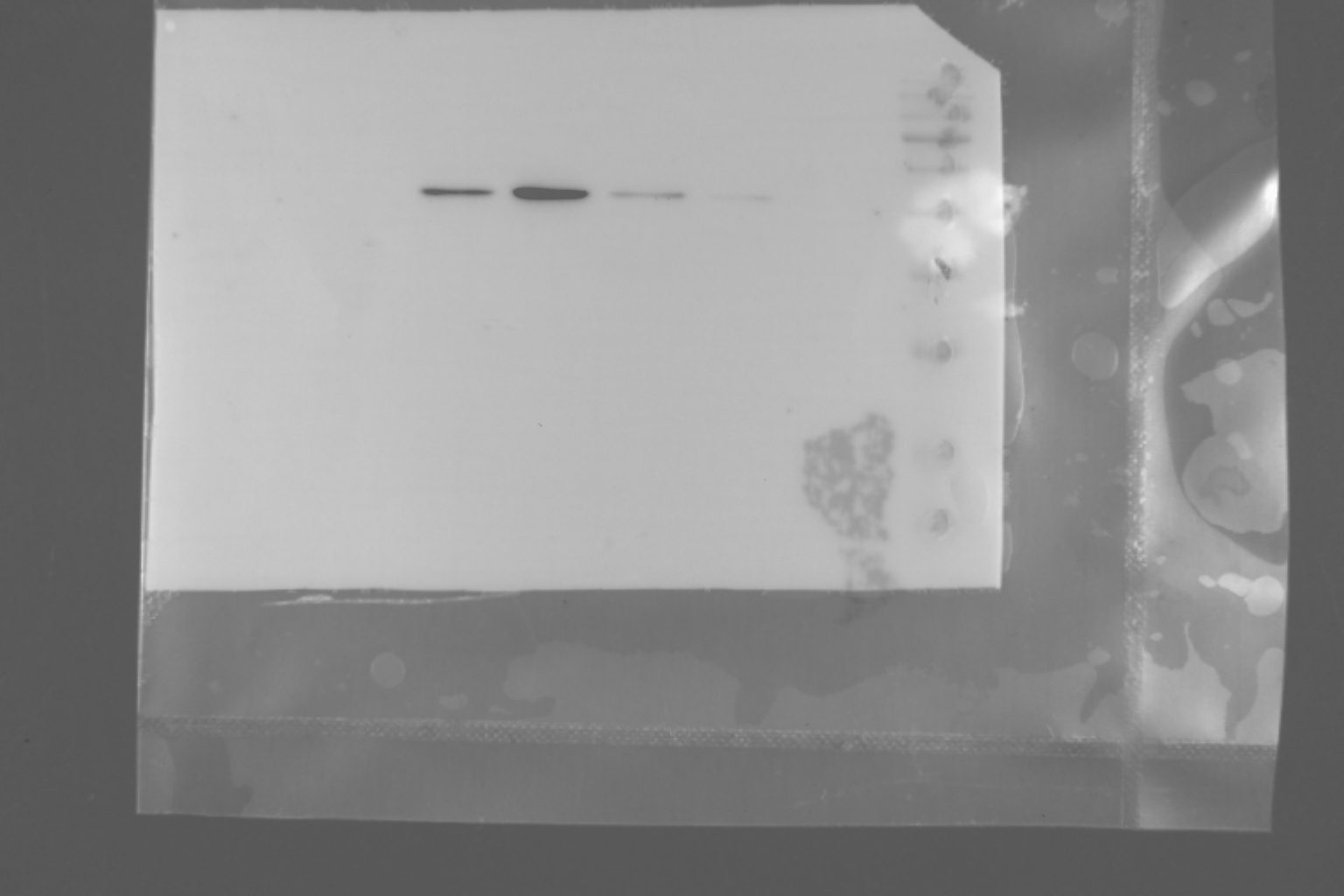

Supplement: Supplementary file 1 [file biomolecules-16-00689-s001.zip › File S1. original WB images/biomolecules-4275260_Original blots/Figure 5C, pp53/Figure 5C, pp53 overlay with markers.tif]

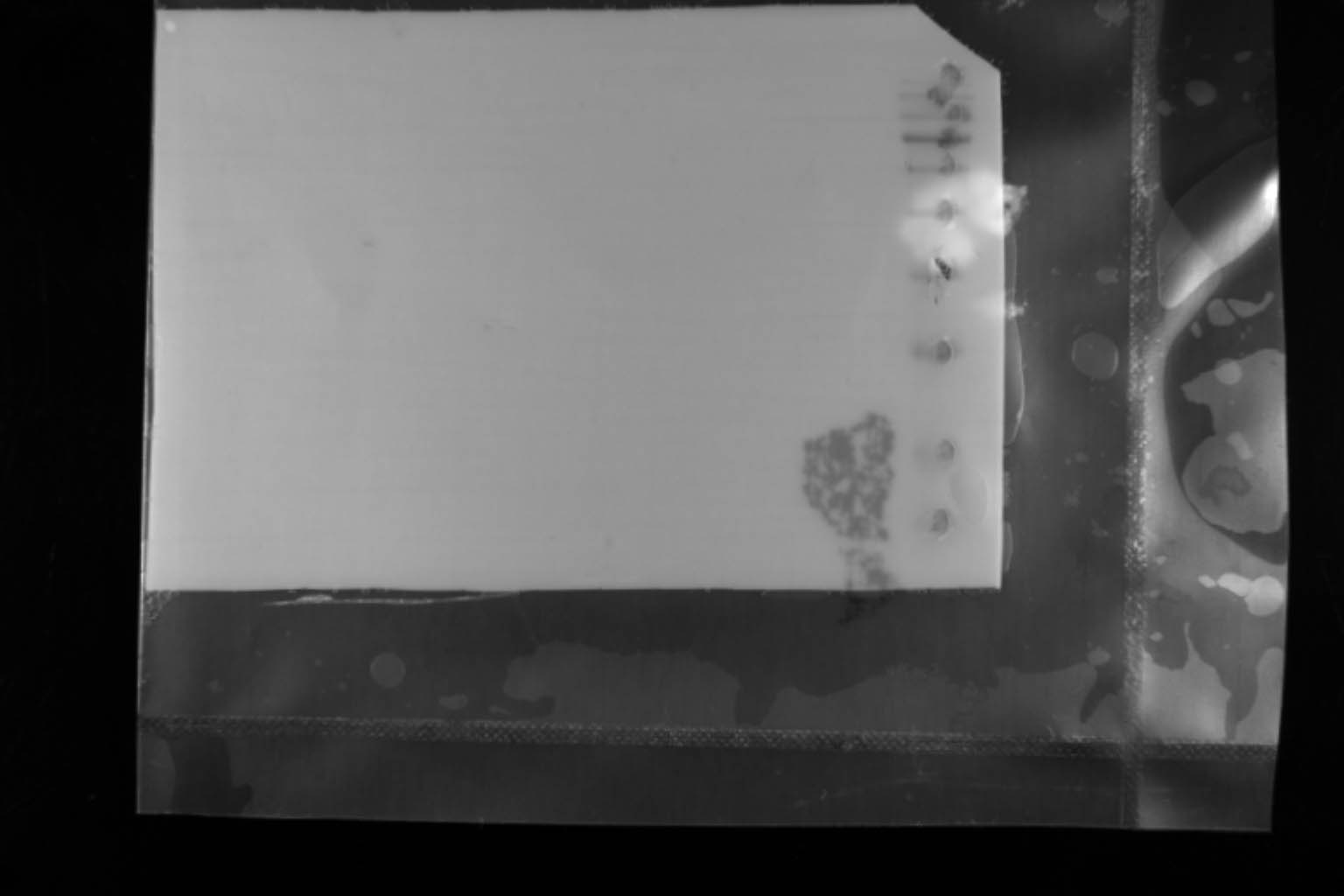

Supplement: Supplementary file 1 [file biomolecules-16-00689-s001.zip › File S1. original WB images/biomolecules-4275260_Original blots/Figure 5C, pp53/markers.jpg]

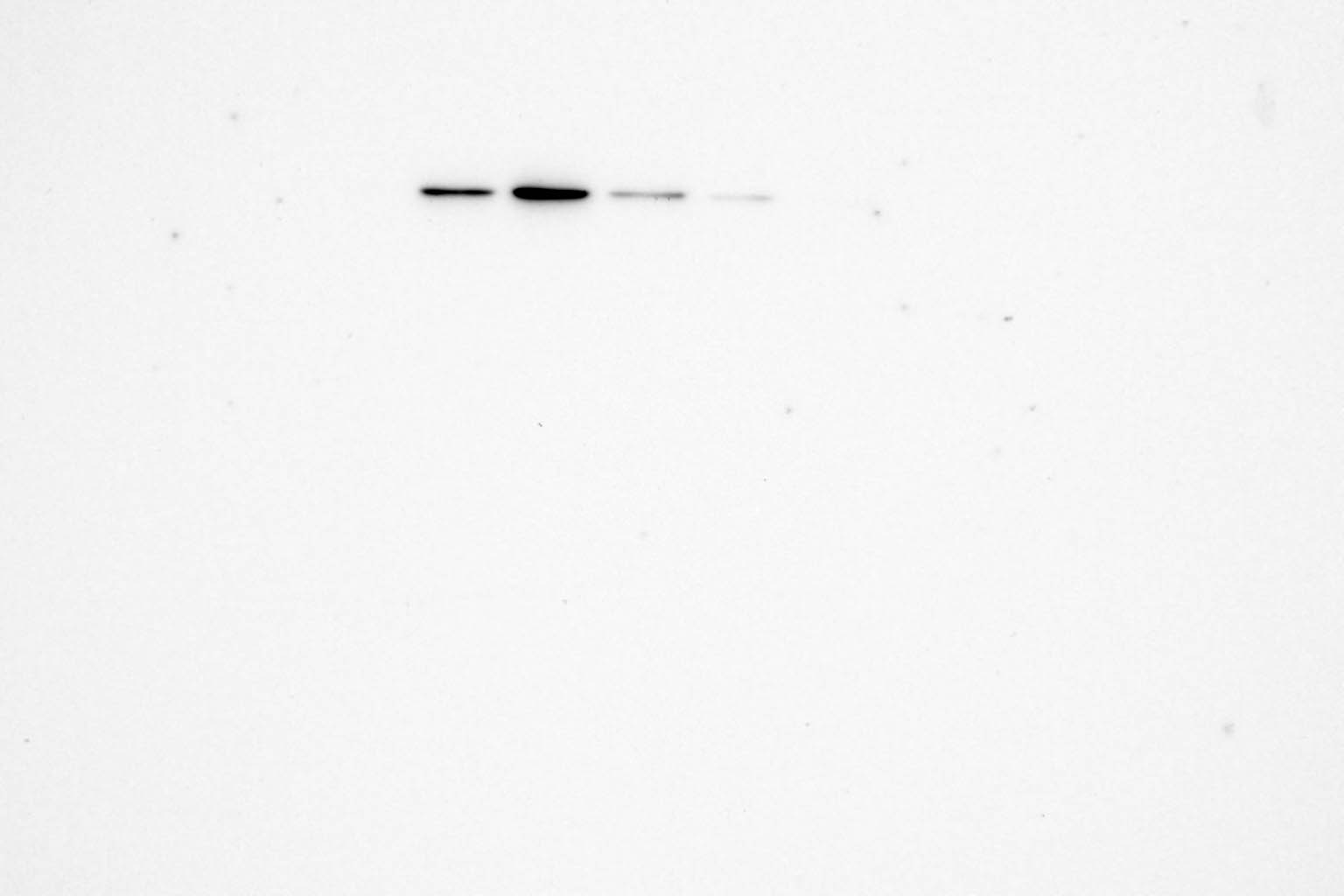

Supplement: Supplementary file 1 [file biomolecules-16-00689-s001.zip › File S1. original WB images/biomolecules-4275260_Original blots/Figure 5C, pp53/pp53.jpg]

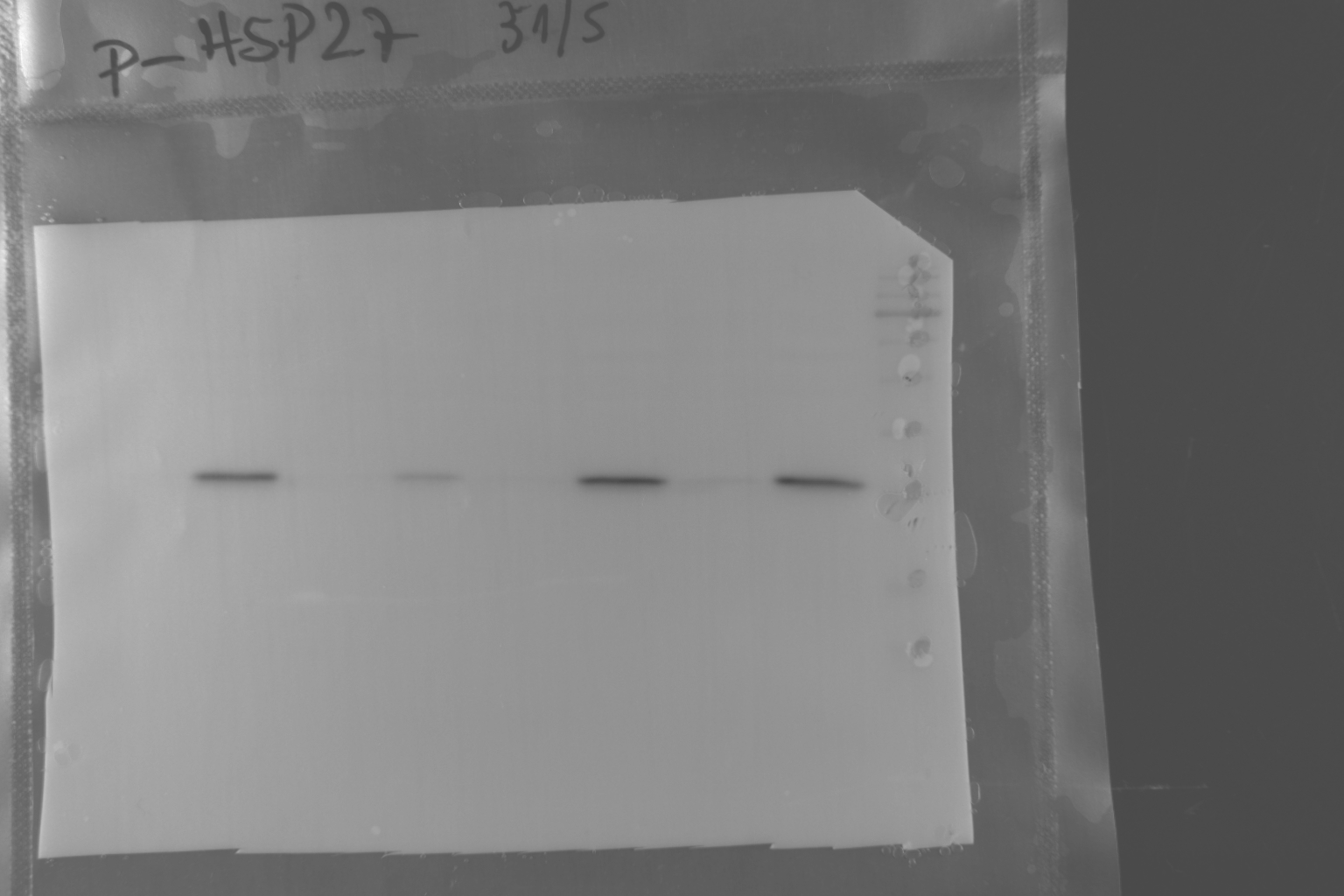

Supplement: Supplementary file 1 [file biomolecules-16-00689-s001.zip › File S1. original WB images/biomolecules-4275260_Original blots/Figure 7A, pHSP27 and GAPDH/Figure 7A, pHSP27 overlay with markers.tif]

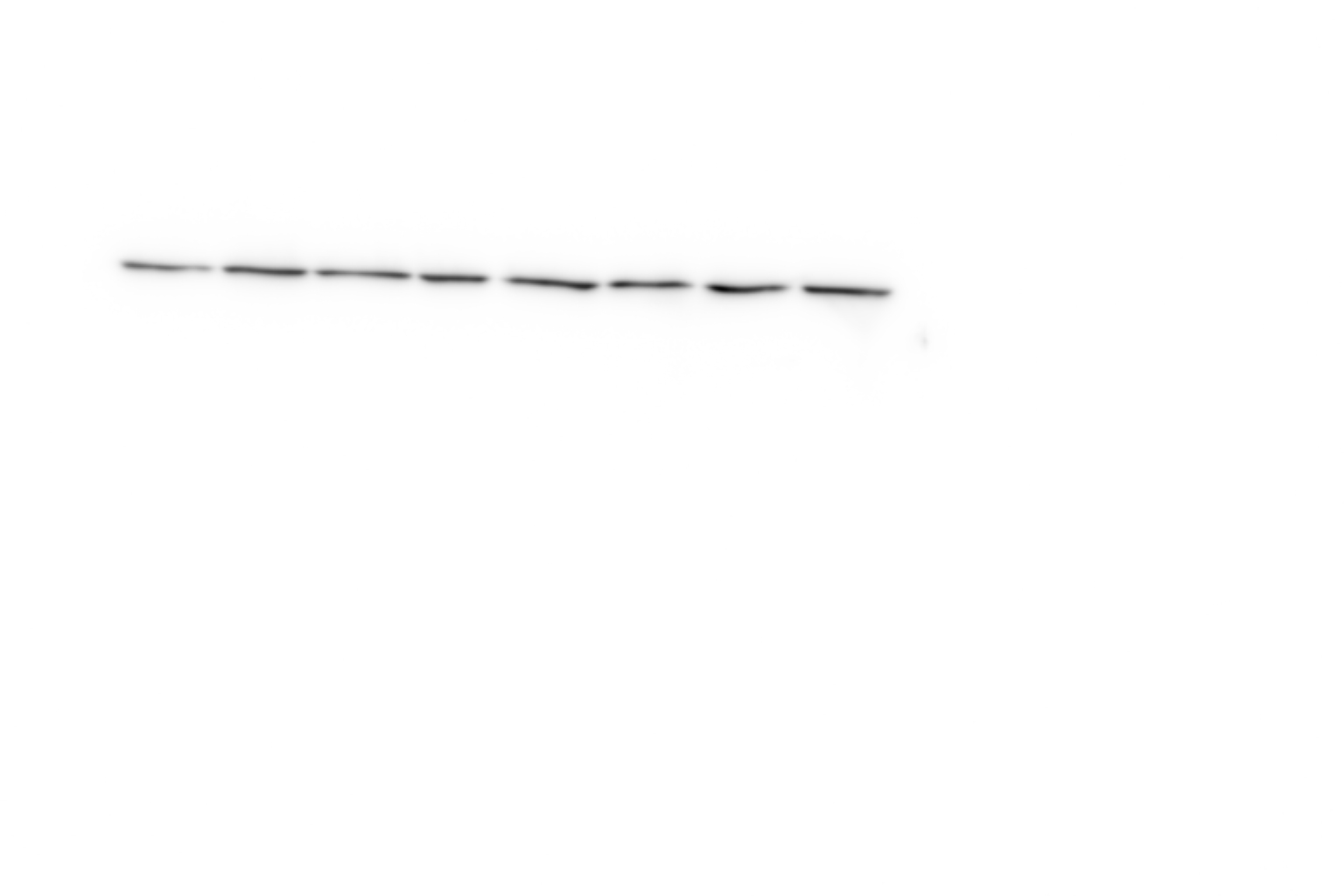

Supplement: Supplementary file 1 [file biomolecules-16-00689-s001.zip › File S1. original WB images/biomolecules-4275260_Original blots/Figure 7A, pHSP27 and GAPDH/Figure 7A_GAPDH.tif]

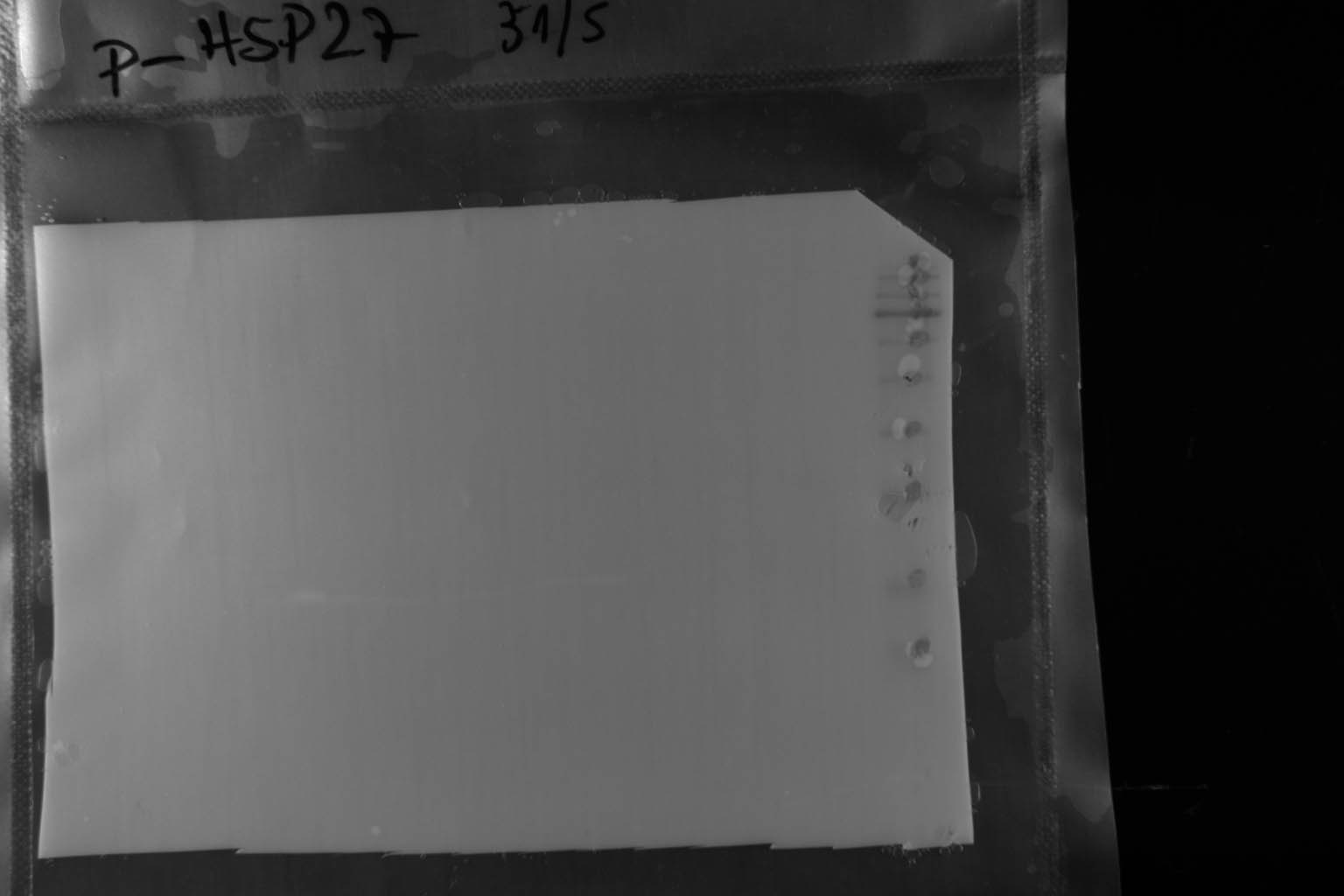

Supplement: Supplementary file 1 [file biomolecules-16-00689-s001.zip › File S1. original WB images/biomolecules-4275260_Original blots/Figure 7A, pHSP27 and GAPDH/markers pHSP27.jpg]

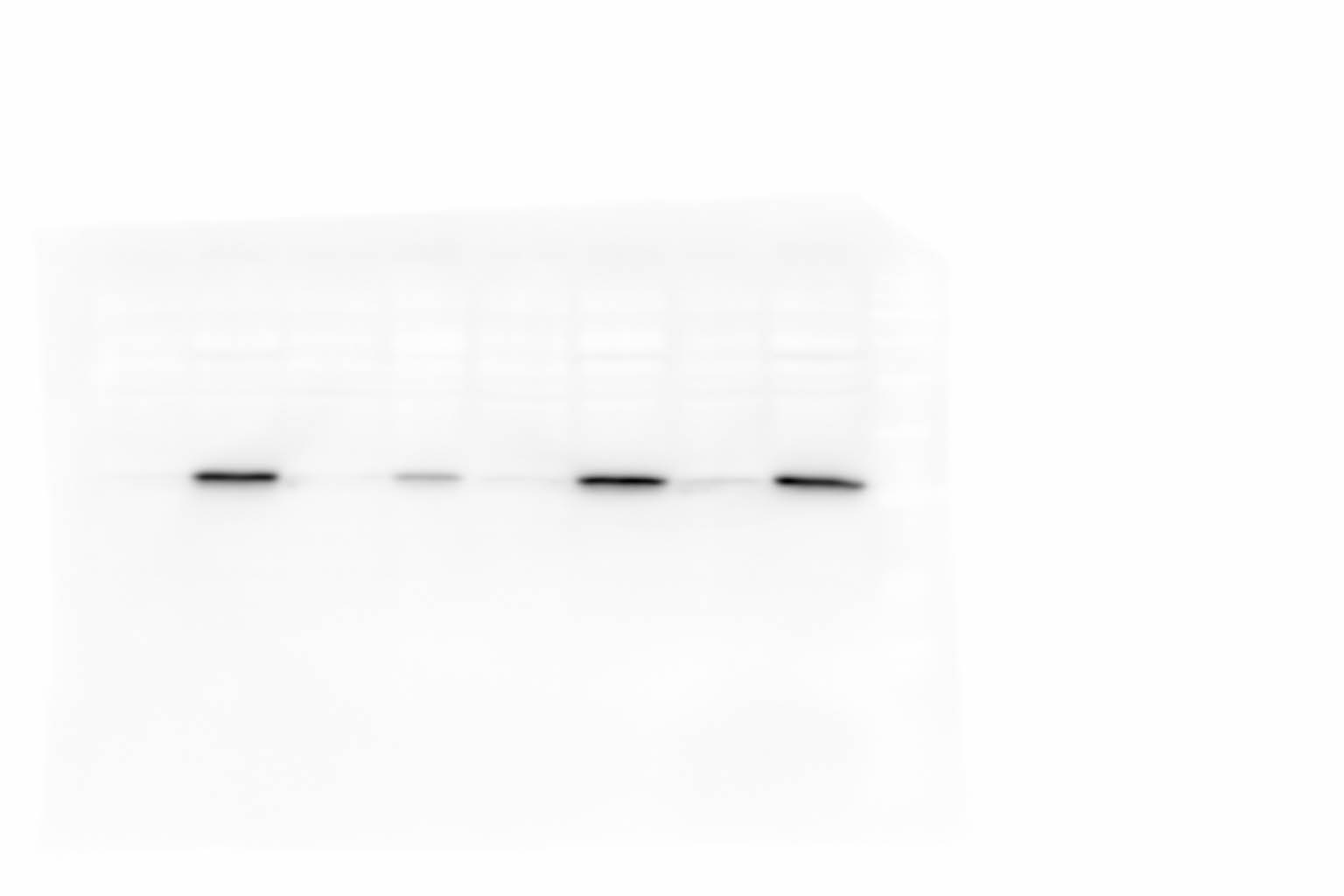

Supplement: Supplementary file 1 [file biomolecules-16-00689-s001.zip › File S1. original WB images/biomolecules-4275260_Original blots/Figure 7A, pHSP27 and GAPDH/pHSP27.jpg]

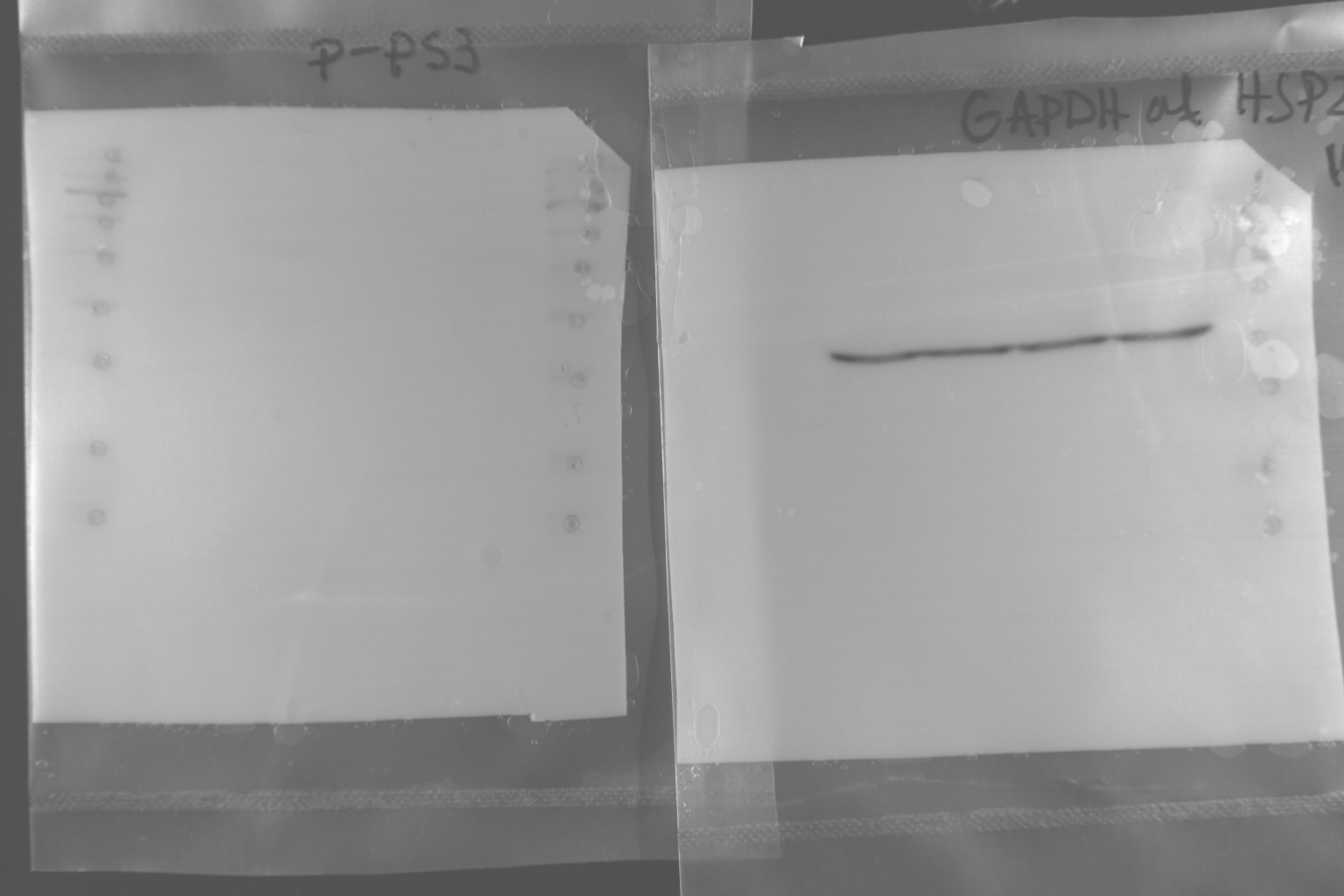

Supplement: Supplementary file 1 [file biomolecules-16-00689-s001.zip › File S1. original WB images/biomolecules-4275260_Original blots/Figure 7B, pHSP27 and GAPDH/Figure 7B, GAPDH overlay.tif]

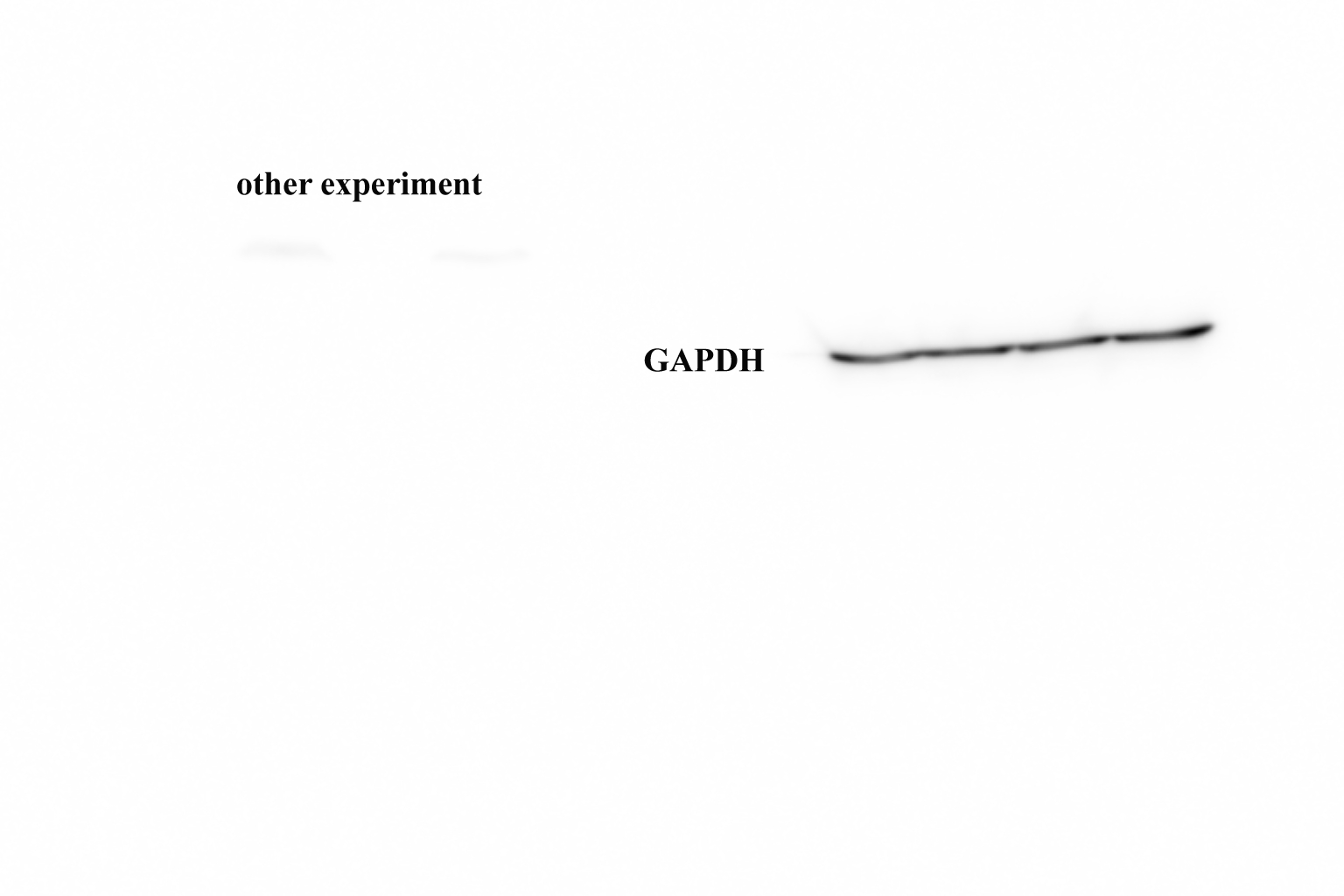

Supplement: Supplementary file 1 [file biomolecules-16-00689-s001.zip › File S1. original WB images/biomolecules-4275260_Original blots/Figure 7B, pHSP27 and GAPDH/Figure 7B_GAPDH.tif]

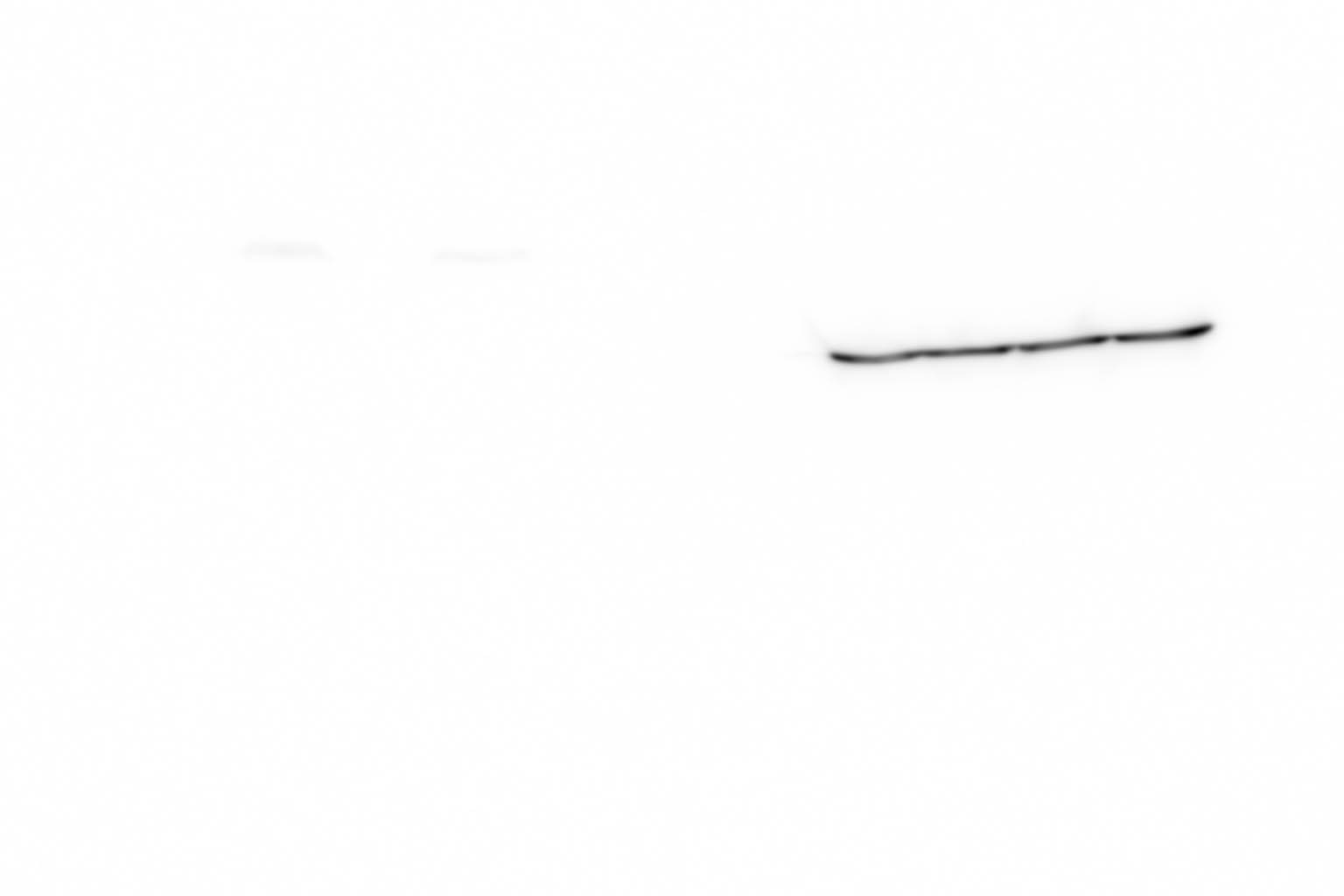

Supplement: Supplementary file 1 [file biomolecules-16-00689-s001.zip › File S1. original WB images/biomolecules-4275260_Original blots/Figure 7B, pHSP27 and GAPDH/GAPDH.jpg]

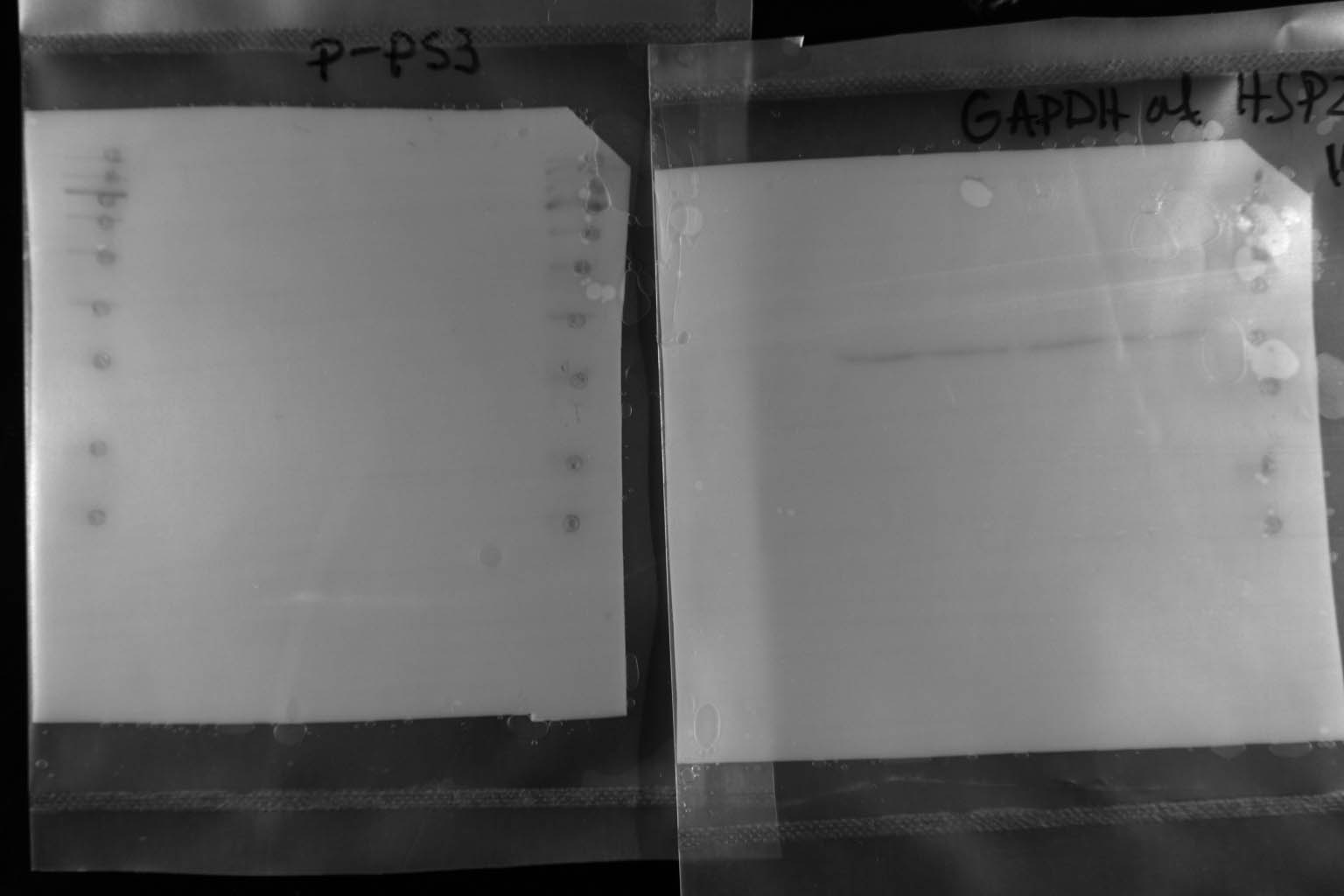

Supplement: Supplementary file 1 [file biomolecules-16-00689-s001.zip › File S1. original WB images/biomolecules-4275260_Original blots/Figure 7B, pHSP27 and GAPDH/markers GAPDH.jpg]

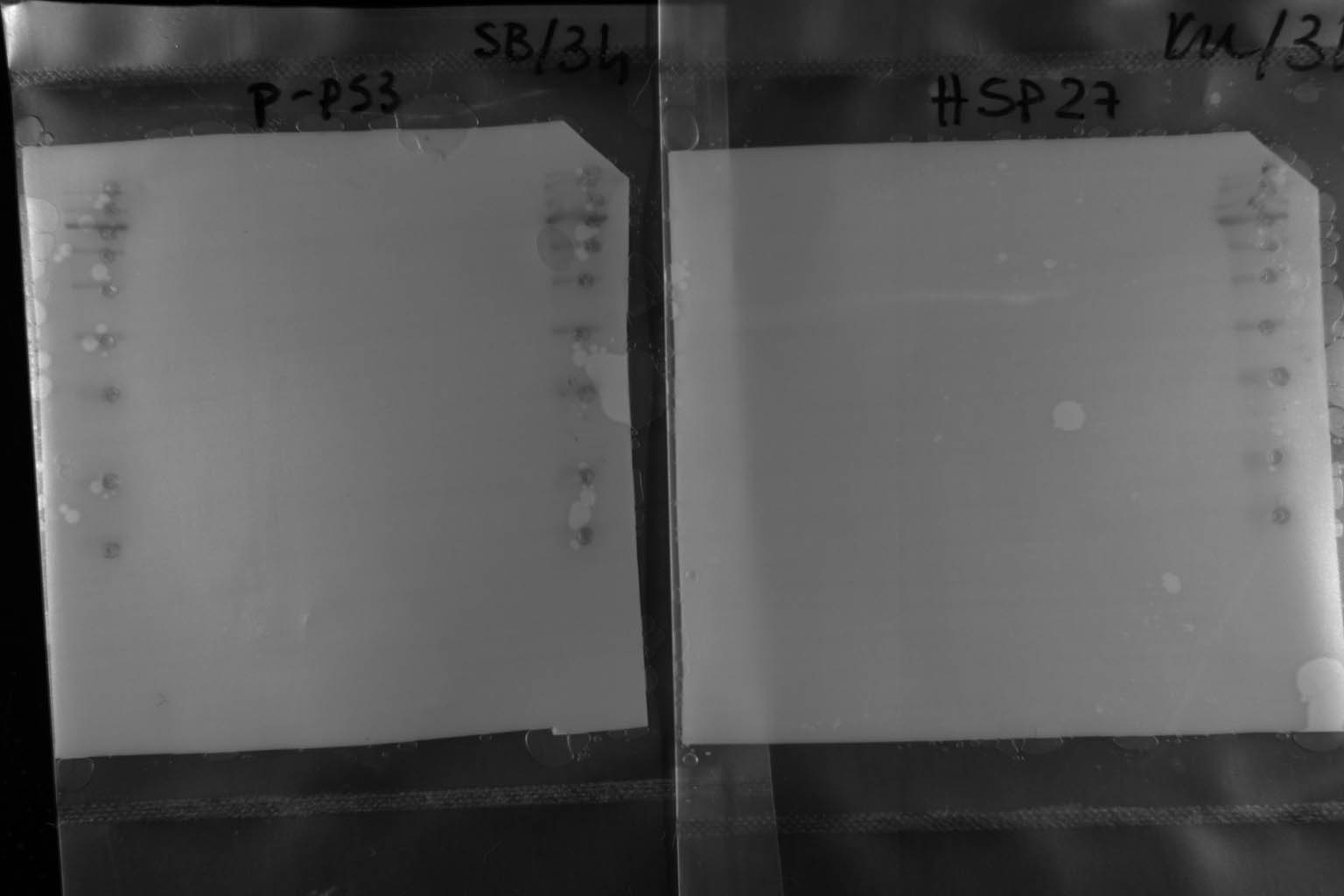

Supplement: Supplementary file 1 [file biomolecules-16-00689-s001.zip › File S1. original WB images/biomolecules-4275260_Original blots/Figure 7B, pHSP27 and GAPDH/markers pHSP27.jpg]

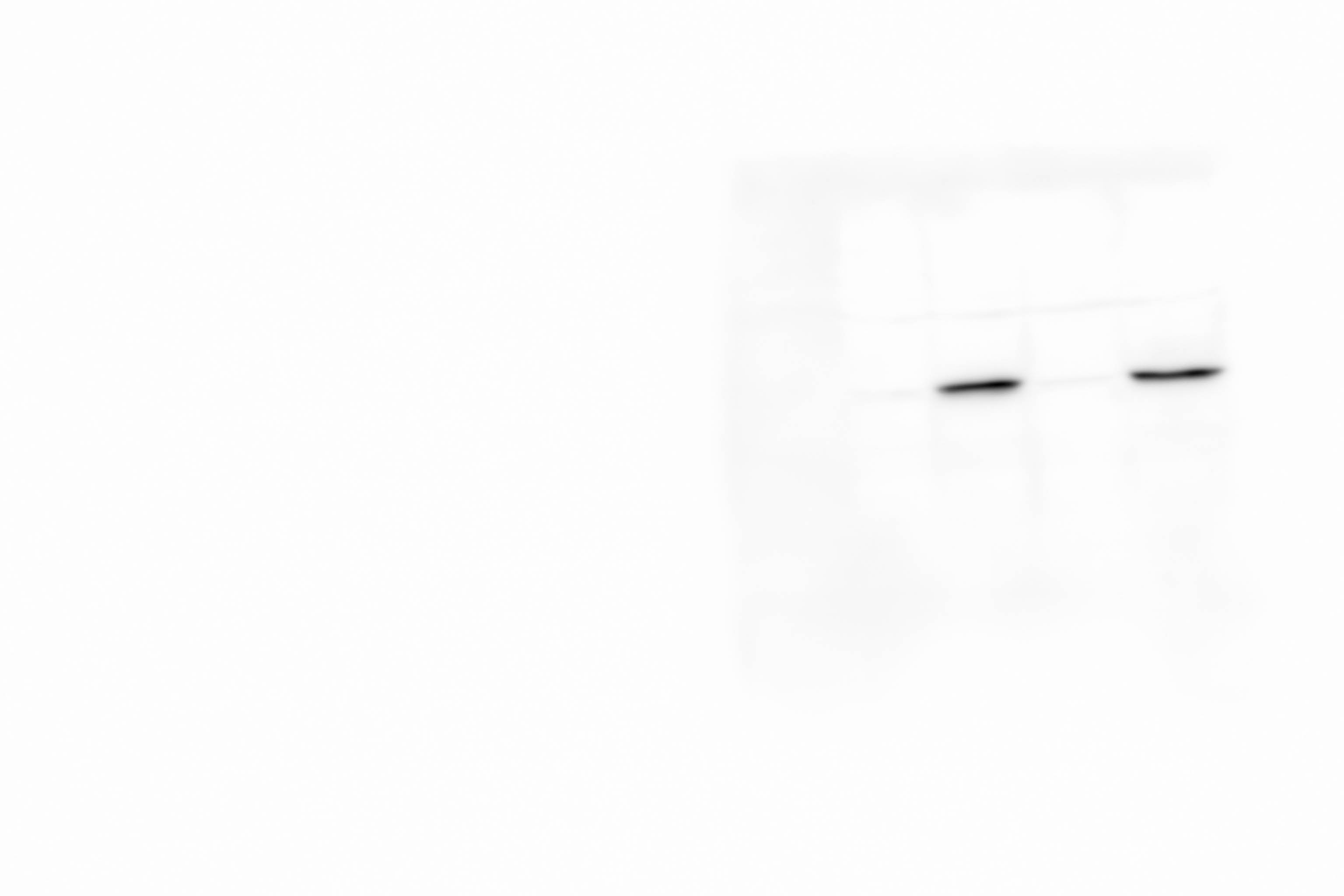

Supplement: Supplementary file 1 [file biomolecules-16-00689-s001.zip › File S1. original WB images/biomolecules-4275260_Original blots/Figure 7B, pHSP27 and GAPDH/pHSP27.jpg]

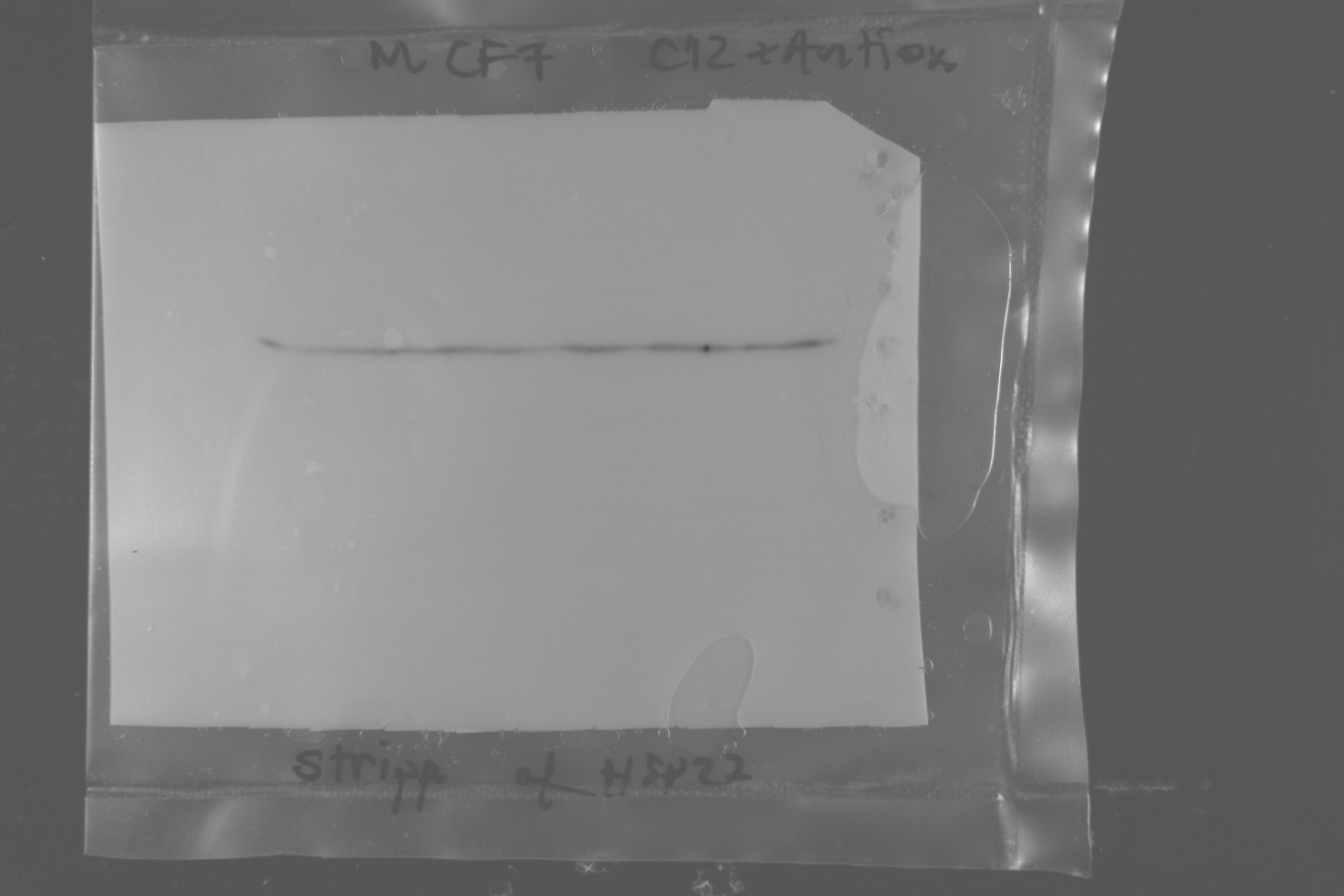

Supplement: Supplementary file 1 [file biomolecules-16-00689-s001.zip › File S1. original WB images/biomolecules-4275260_Original blots/Figure 9B, pHSP27 and GAPDH/Figure 9B, GAPDH overlay with markers.tif]

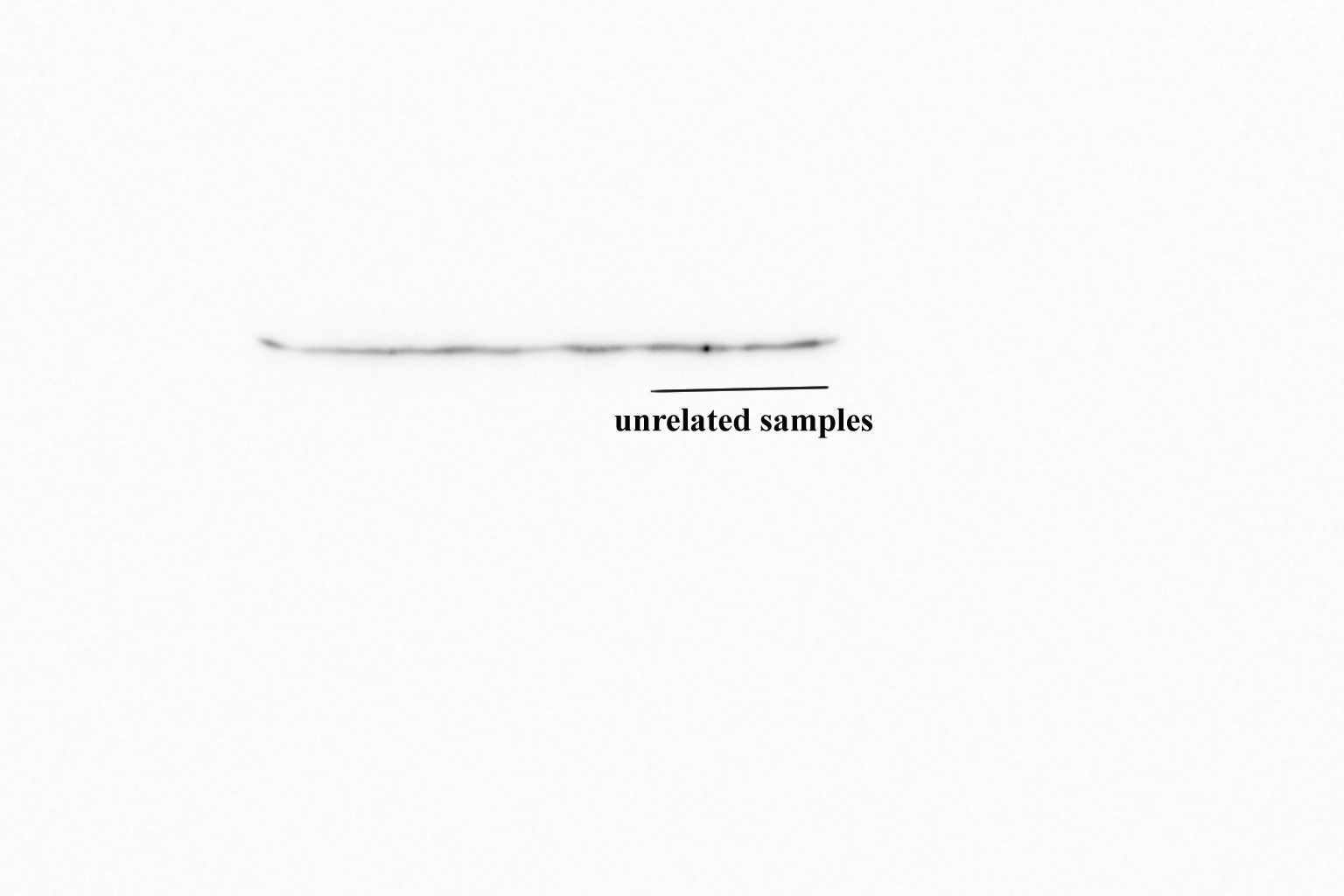

Supplement: Supplementary file 1 [file biomolecules-16-00689-s001.zip › File S1. original WB images/biomolecules-4275260_Original blots/Figure 9B, pHSP27 and GAPDH/Figure 9B_GAPDH.tif]

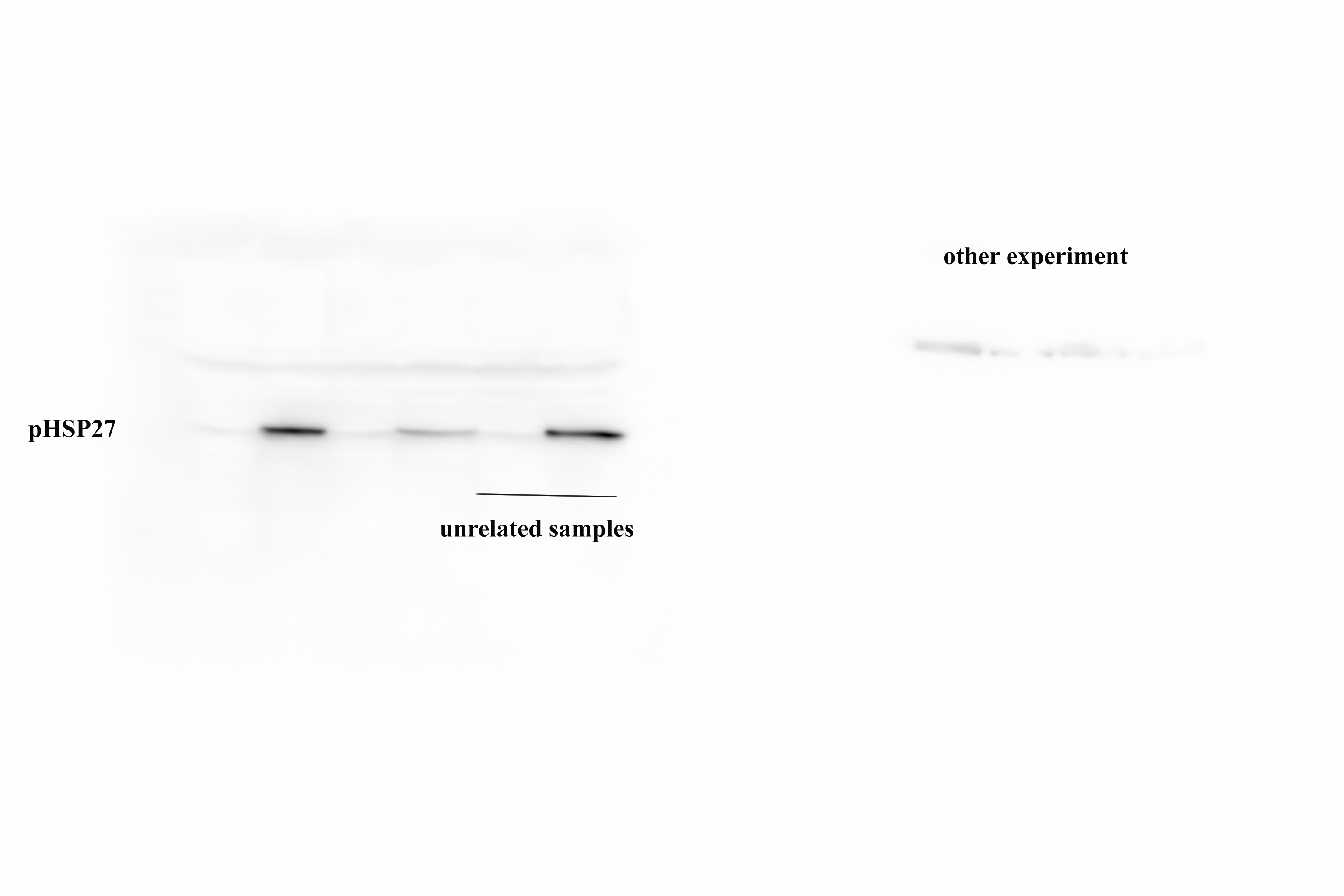

Supplement: Supplementary file 1 [file biomolecules-16-00689-s001.zip › File S1. original WB images/biomolecules-4275260_Original blots/Figure 9B, pHSP27 and GAPDH/Figure 9B_pHSP27.tif]

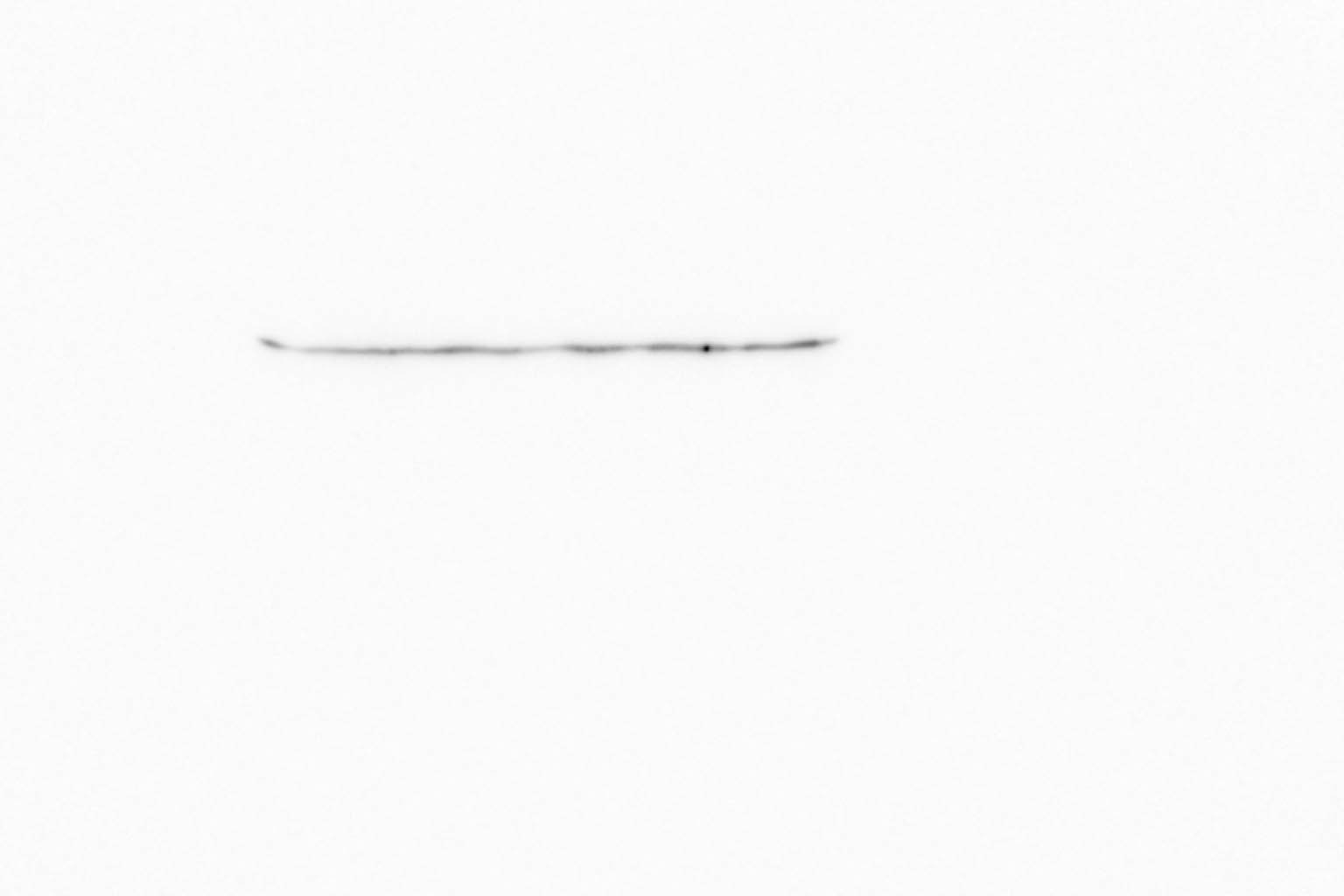

Supplement: Supplementary file 1 [file biomolecules-16-00689-s001.zip › File S1. original WB images/biomolecules-4275260_Original blots/Figure 9B, pHSP27 and GAPDH/GAPDH.jpg]

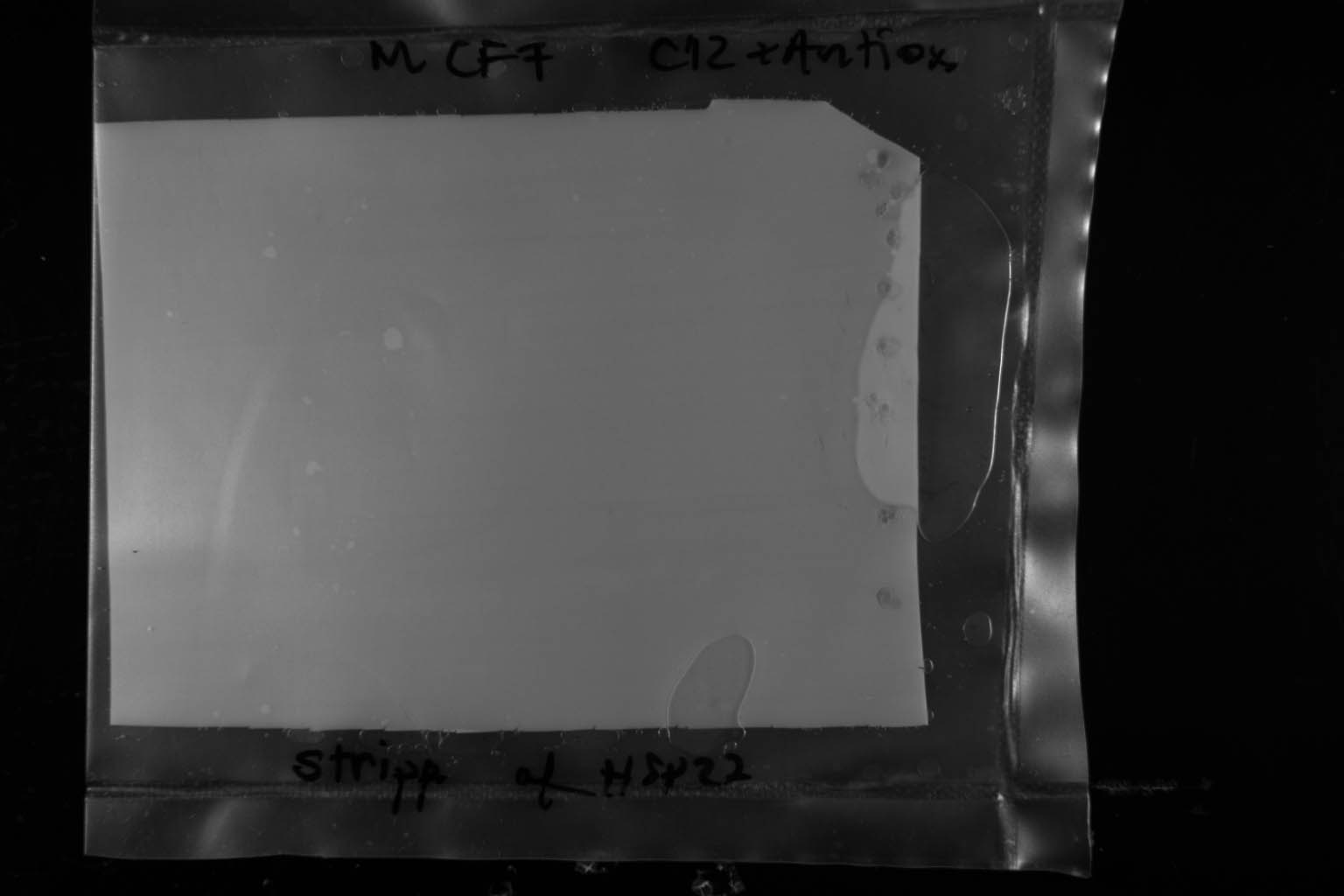

Supplement: Supplementary file 1 [file biomolecules-16-00689-s001.zip › File S1. original WB images/biomolecules-4275260_Original blots/Figure 9B, pHSP27 and GAPDH/markers GAPDH.jpg]

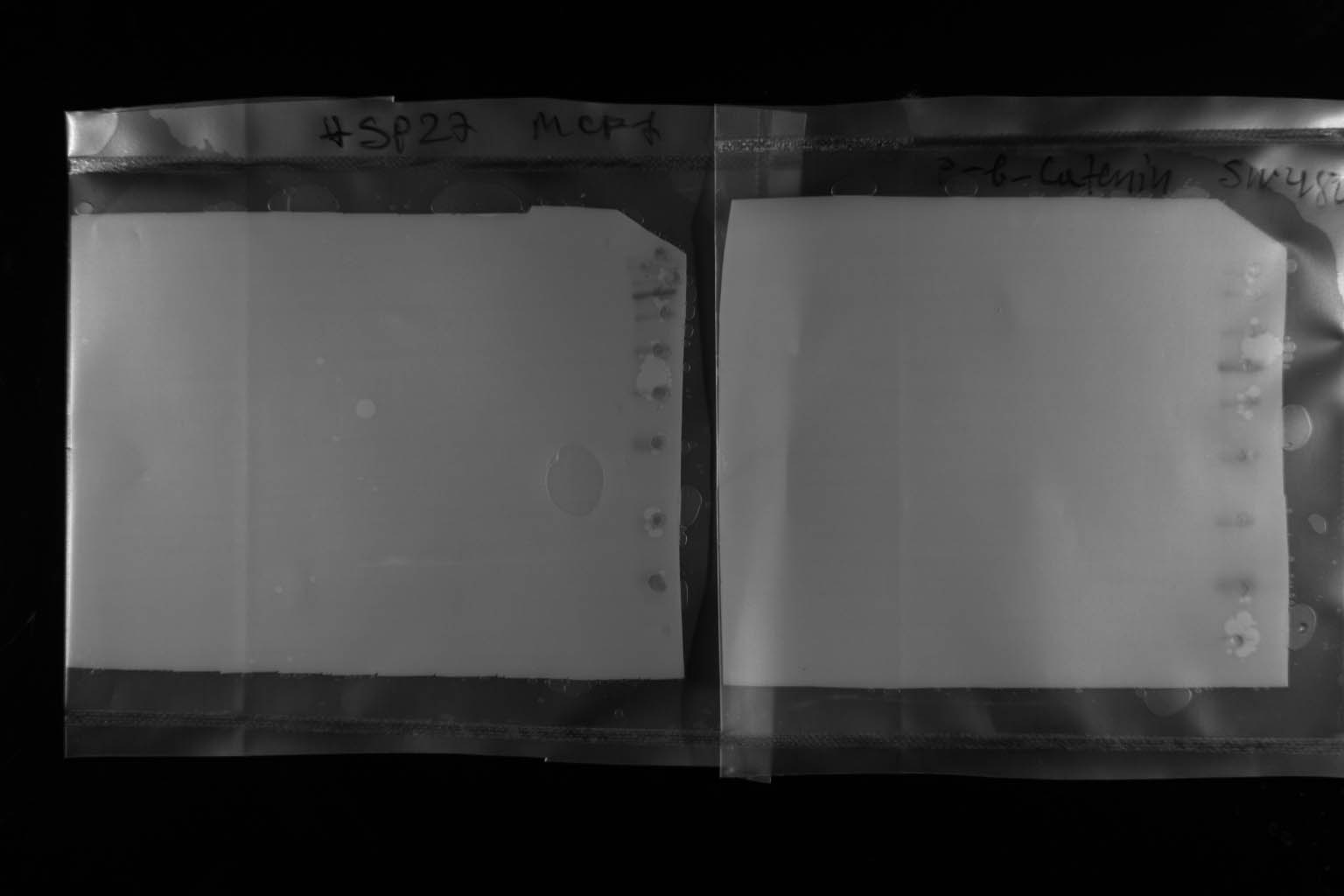

Supplement: Supplementary file 1 [file biomolecules-16-00689-s001.zip › File S1. original WB images/biomolecules-4275260_Original blots/Figure 9B, pHSP27 and GAPDH/markers pHSP27.jpg]

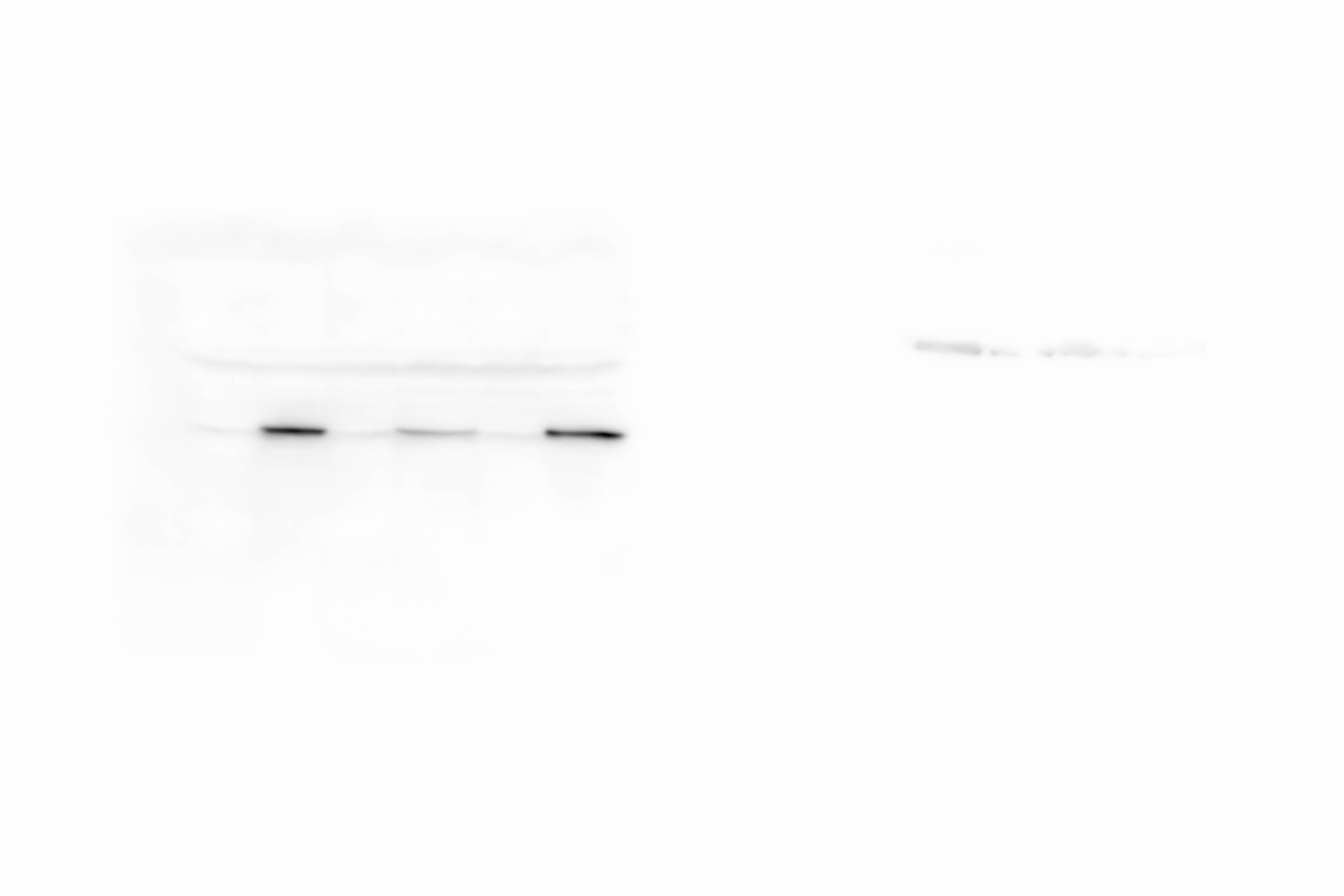

Supplement: Supplementary file 1 [file biomolecules-16-00689-s001.zip › File S1. original WB images/biomolecules-4275260_Original blots/Figure 9B, pHSP27 and GAPDH/pHSP27.jpg]

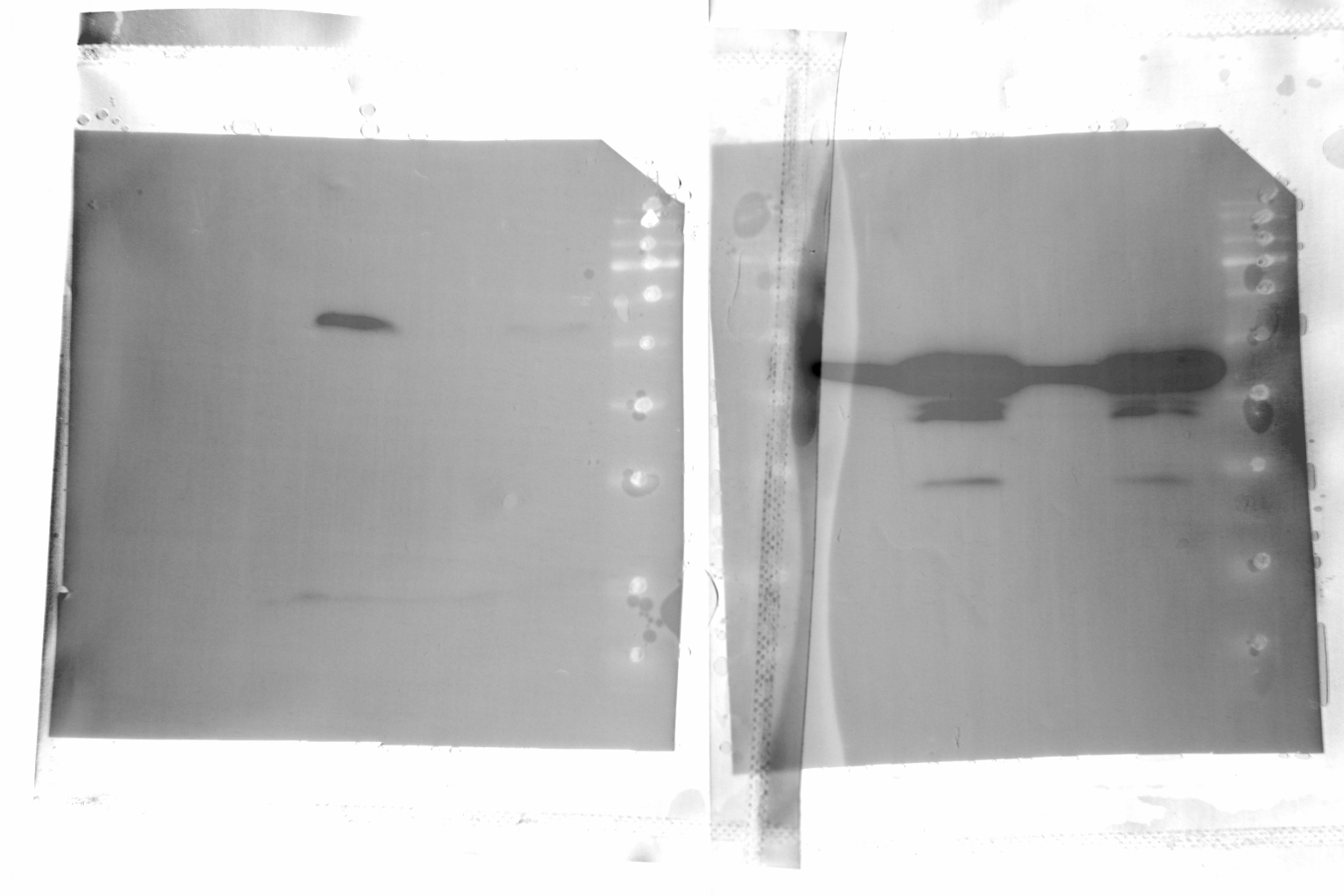

Supplement: Supplementary file 1 [file biomolecules-16-00689-s001.zip › File S1. original WB images/biomolecules-4275260_Original blots/Figure S16, pp53 and GAPDH/Figure S16, pp53.tif]

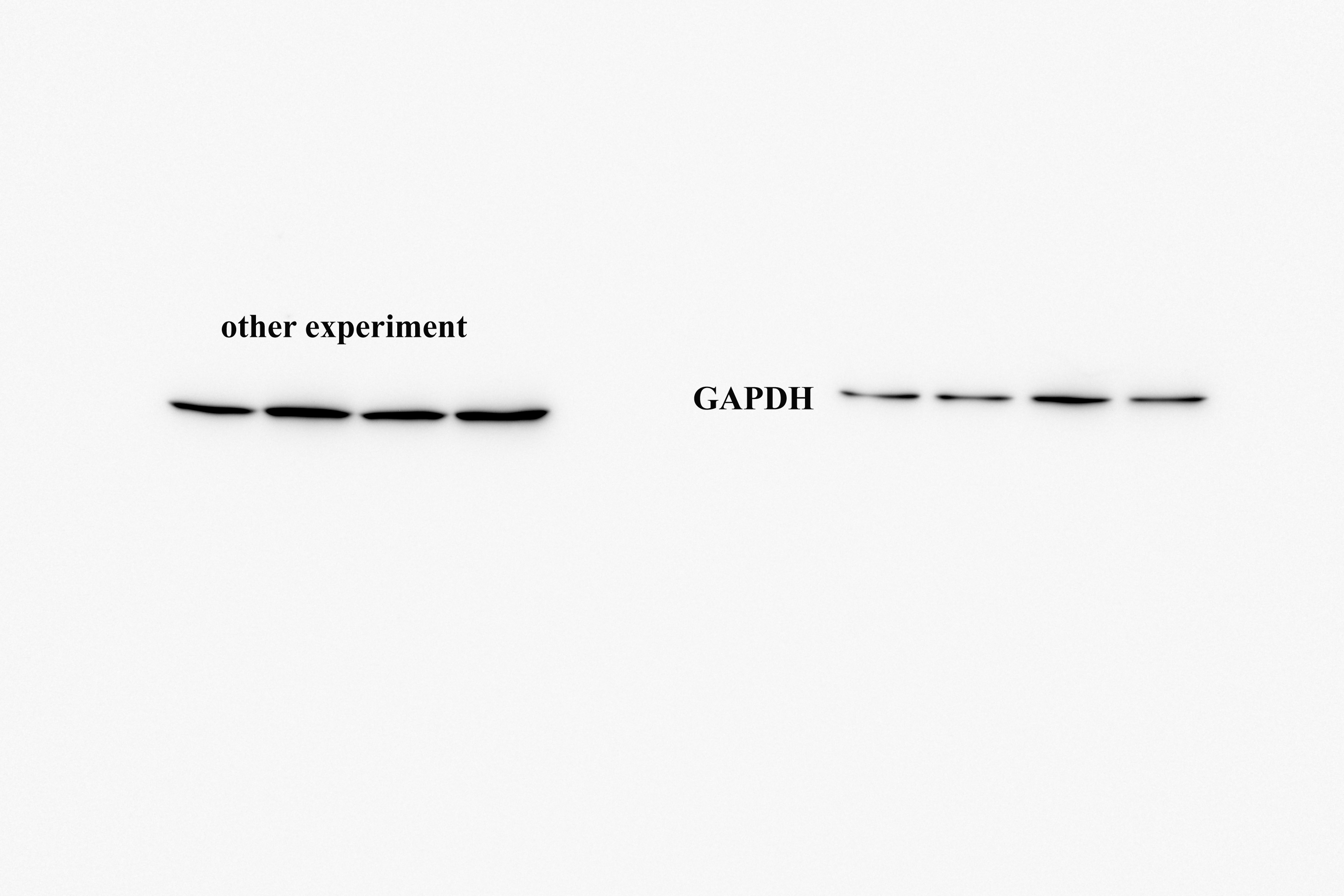

Supplement: Supplementary file 1 [file biomolecules-16-00689-s001.zip › File S1. original WB images/biomolecules-4275260_Original blots/Figure S16, pp53 and GAPDH/Figure S16_GAPDH.tif]

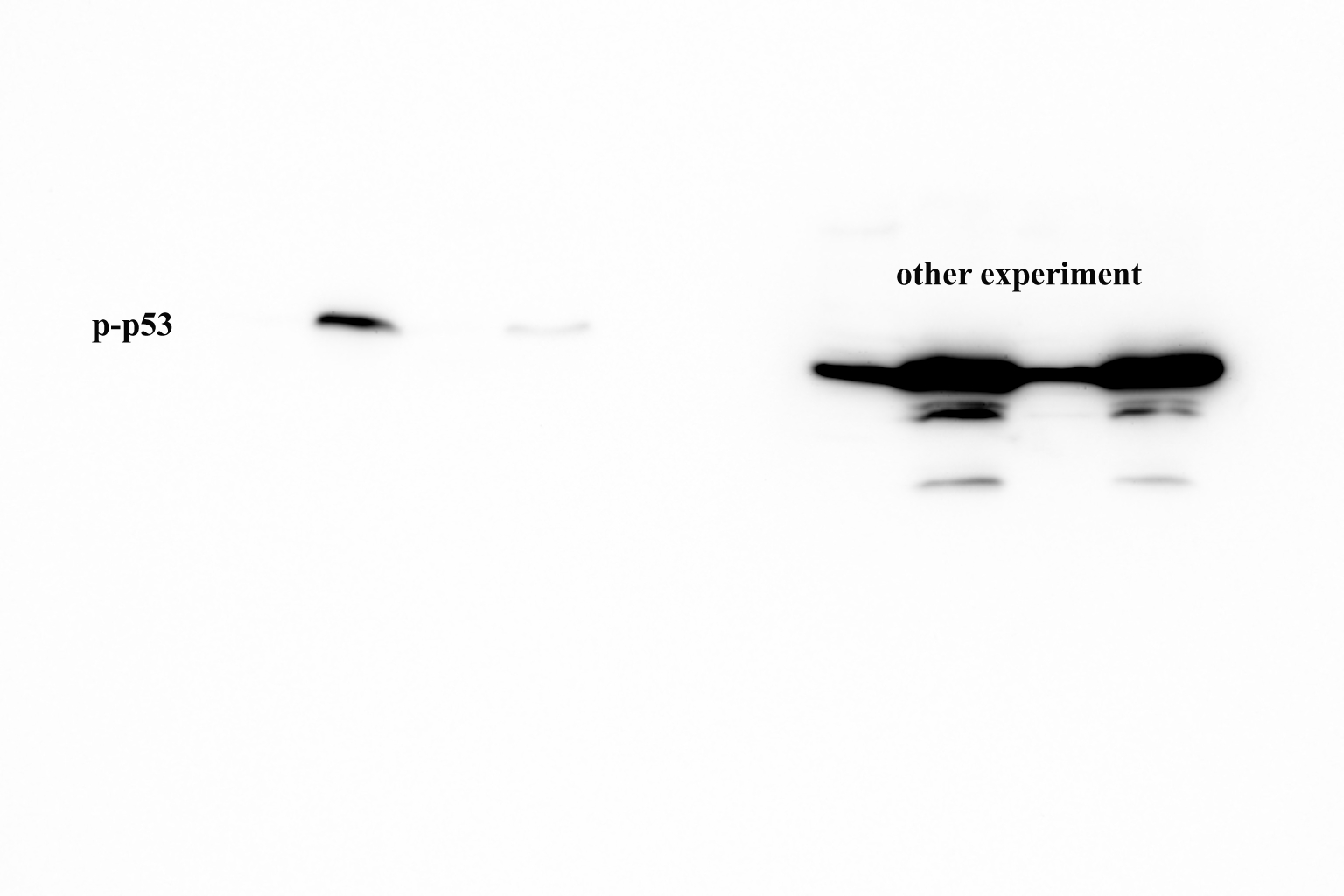

Supplement: Supplementary file 1 [file biomolecules-16-00689-s001.zip › File S1. original WB images/biomolecules-4275260_Original blots/Figure S16, pp53 and GAPDH/Figure S16_pp53.tif]

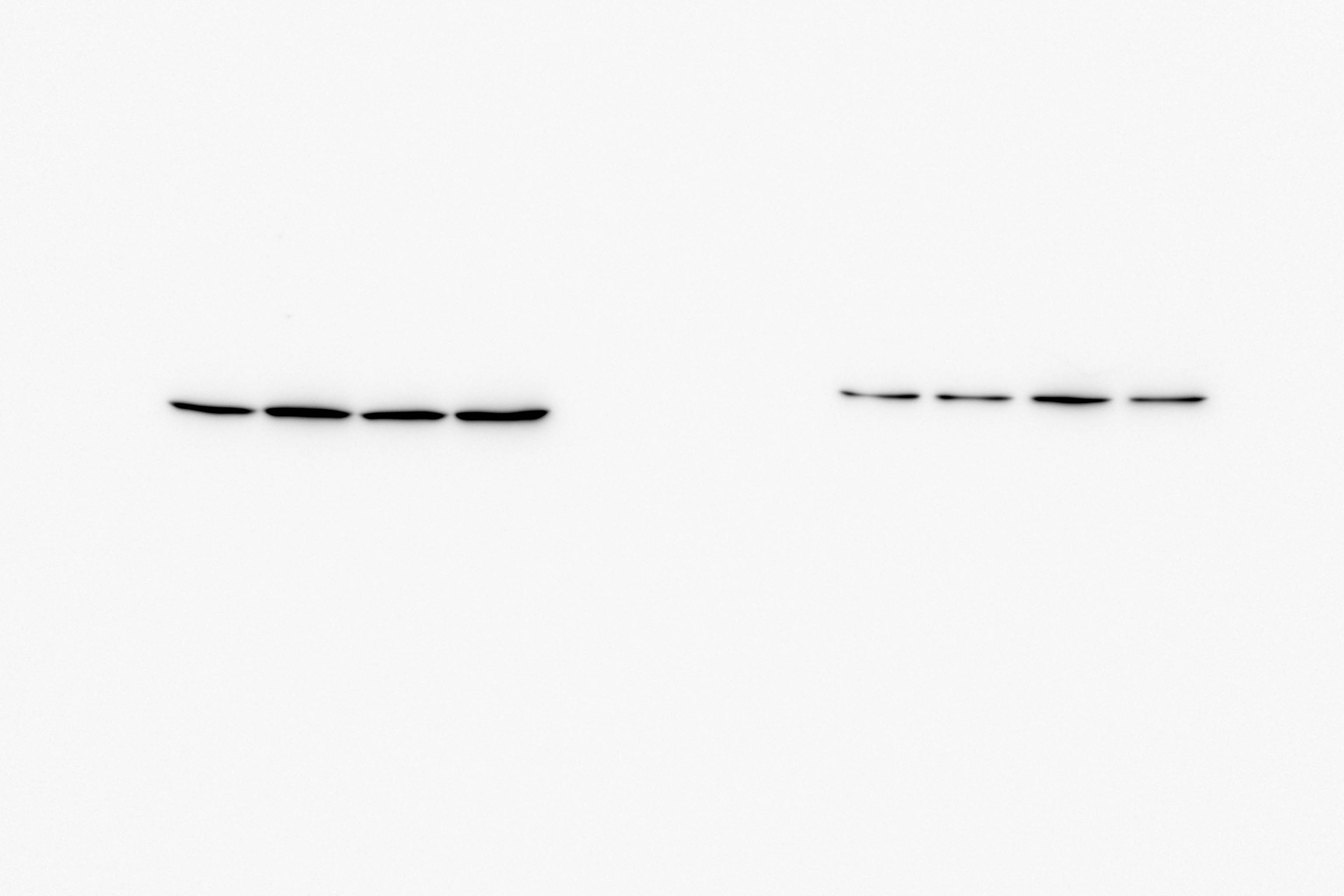

Supplement: Supplementary file 1 [file biomolecules-16-00689-s001.zip › File S1. original WB images/biomolecules-4275260_Original blots/Figure S16, pp53 and GAPDH/GAPDH.jpg]

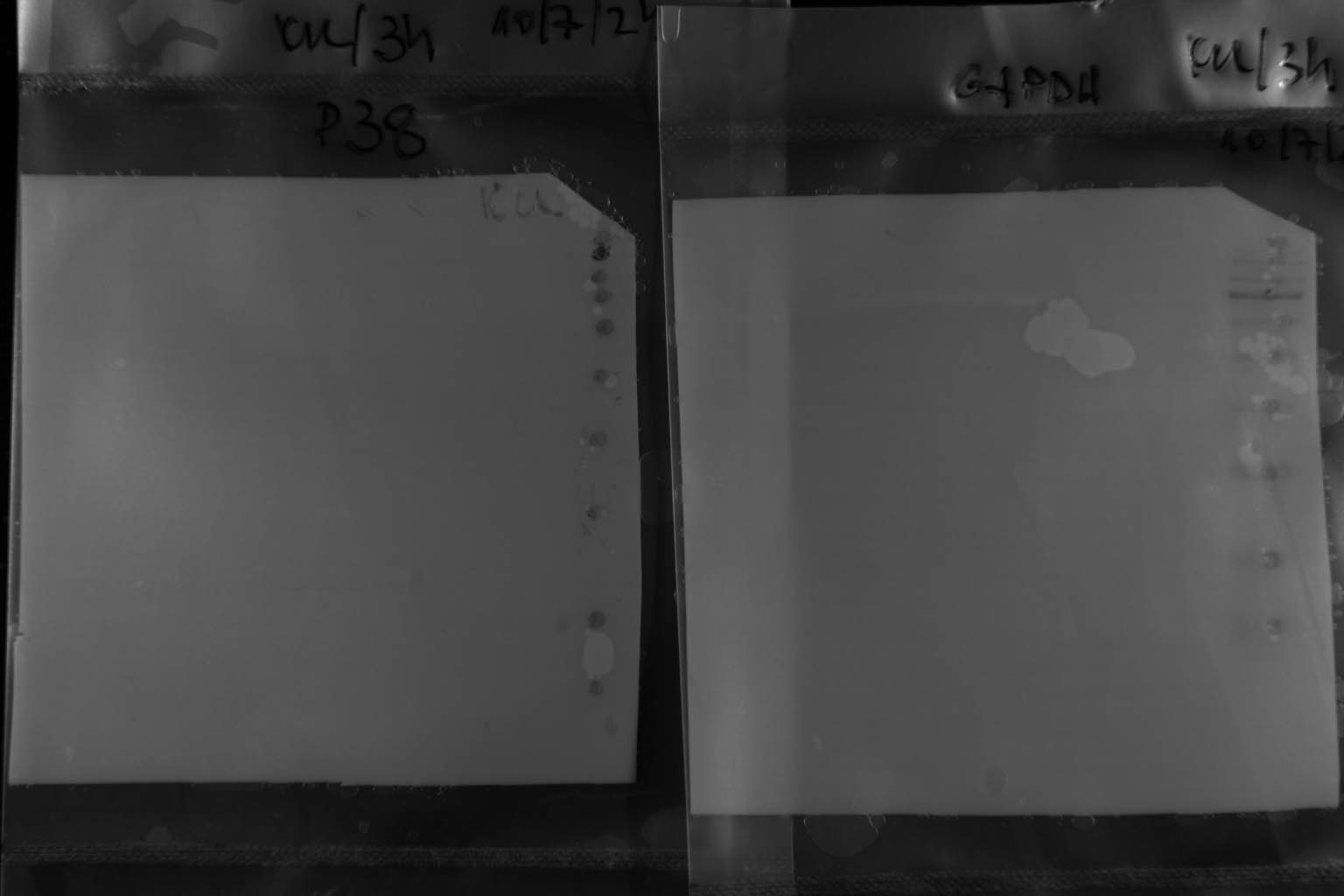

Supplement: Supplementary file 1 [file biomolecules-16-00689-s001.zip › File S1. original WB images/biomolecules-4275260_Original blots/Figure S16, pp53 and GAPDH/markers GAPDH.jpg]

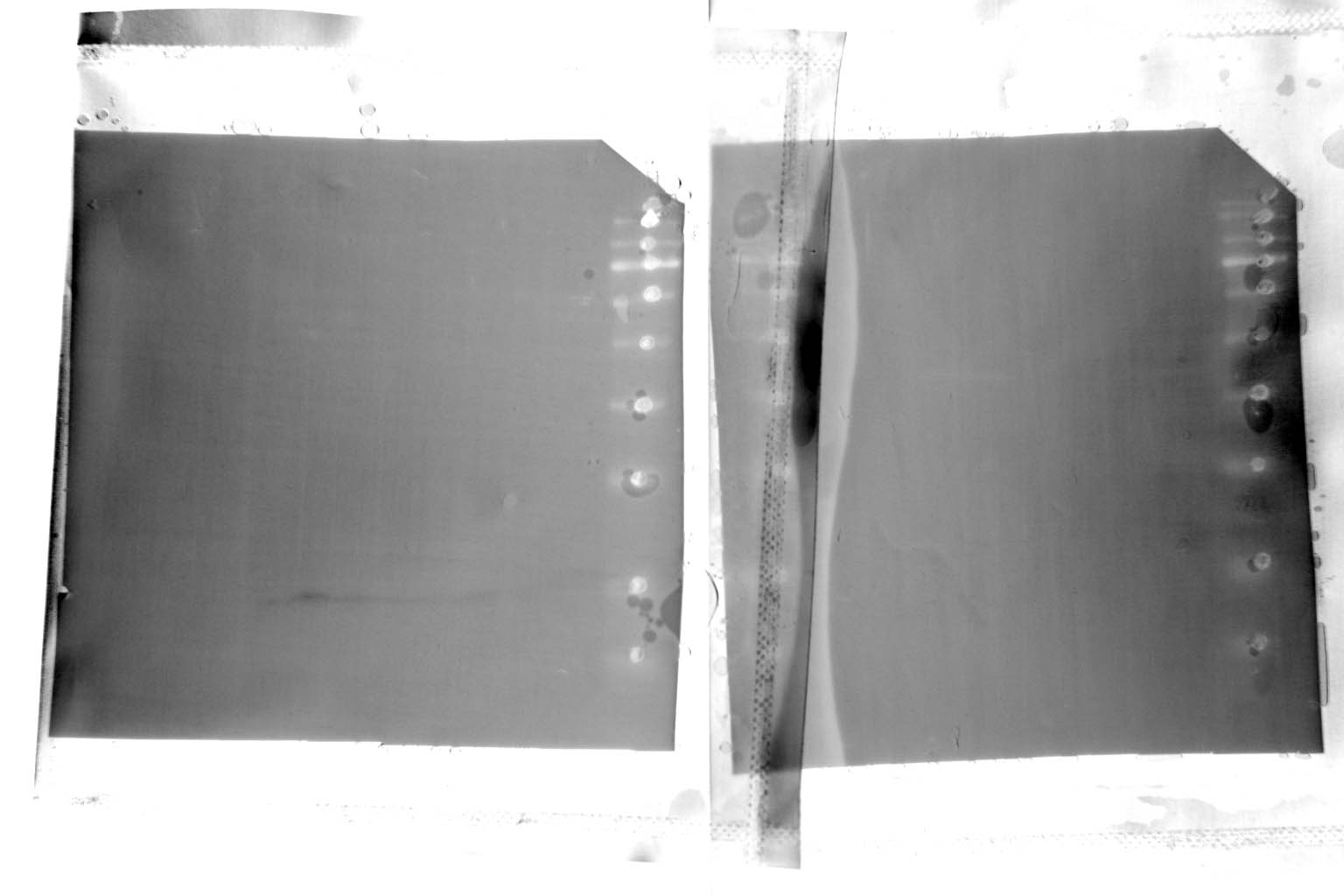

Supplement: Supplementary file 1 [file biomolecules-16-00689-s001.zip › File S1. original WB images/biomolecules-4275260_Original blots/Figure S16, pp53 and GAPDH/markers p53.jpg]

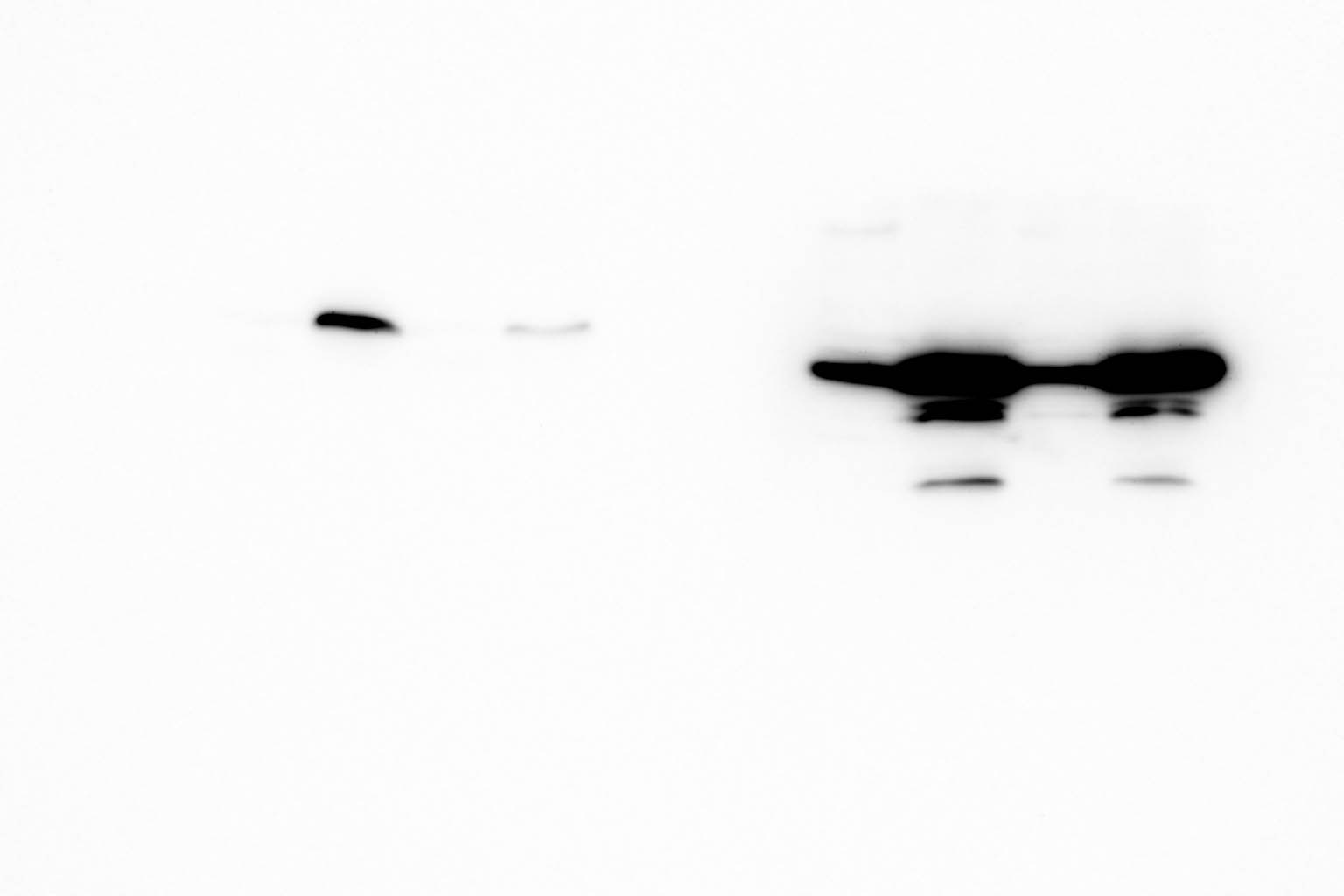

Supplement: Supplementary file 1 [file biomolecules-16-00689-s001.zip › File S1. original WB images/biomolecules-4275260_Original blots/Figure S16, pp53 and GAPDH/pp53.jpg]

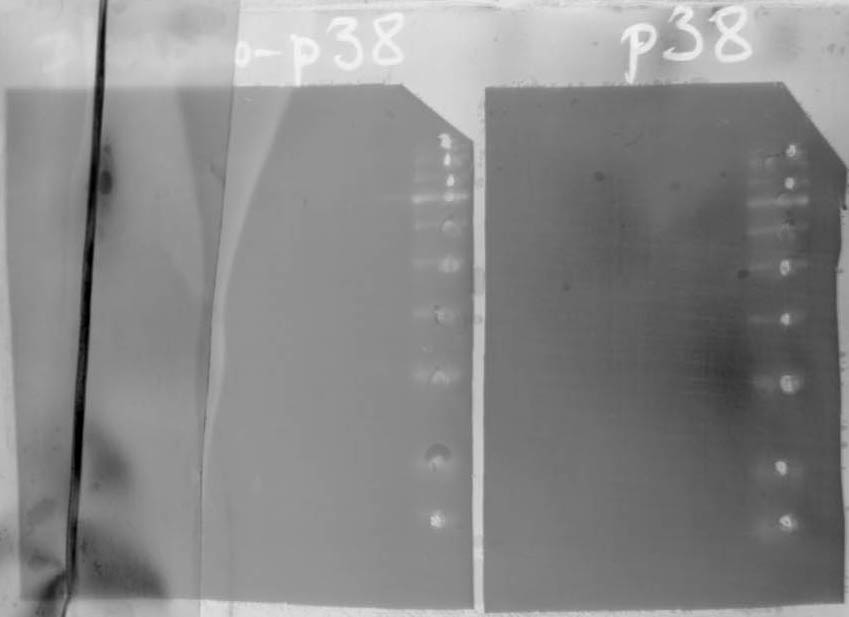

Supplement: Supplementary file 1 [file biomolecules-16-00689-s001.zip › File S1. original WB images/biomolecules-4275260_Original blots/Figure S18_pp38, p38, pHSP27, GAPDH/Figure S18A/markers.jpg]

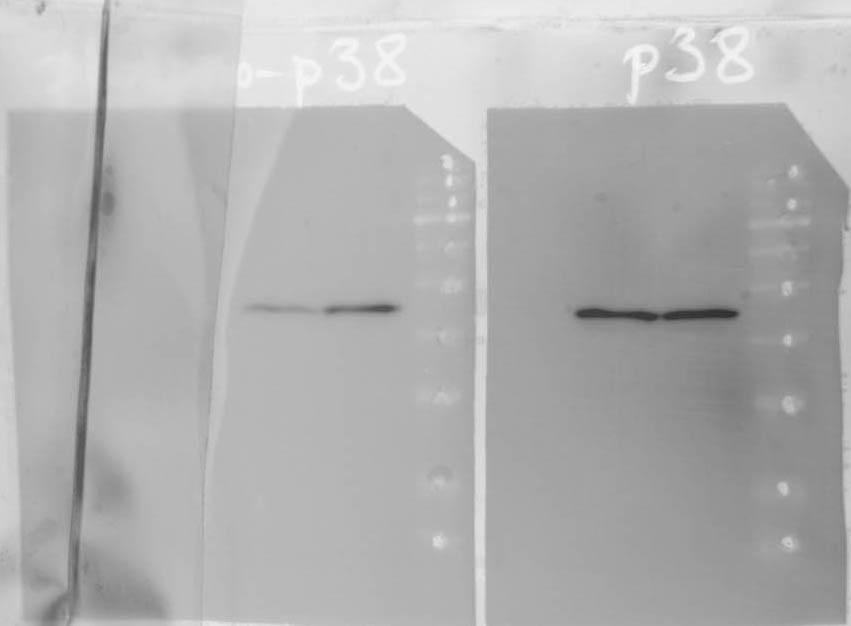

Supplement: Supplementary file 1 [file biomolecules-16-00689-s001.zip › File S1. original WB images/biomolecules-4275260_Original blots/Figure S18_pp38, p38, pHSP27, GAPDH/Figure S18A/p-p38 and p38 overlay with markers.jpg]

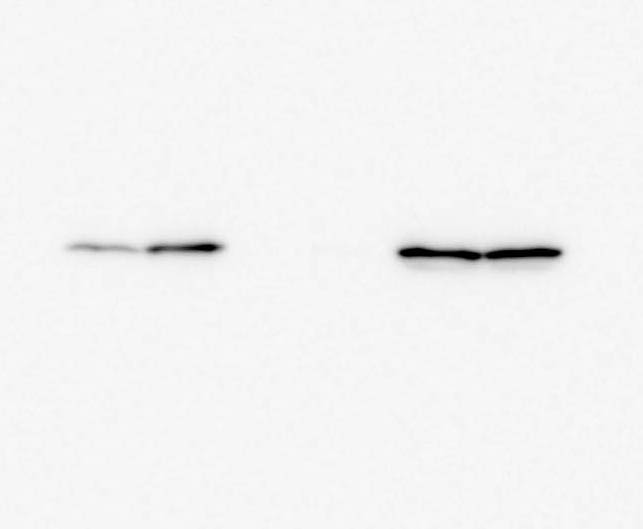

Supplement: Supplementary file 1 [file biomolecules-16-00689-s001.zip › File S1. original WB images/biomolecules-4275260_Original blots/Figure S18_pp38, p38, pHSP27, GAPDH/Figure S18A/p-p38 and p38.jpg]

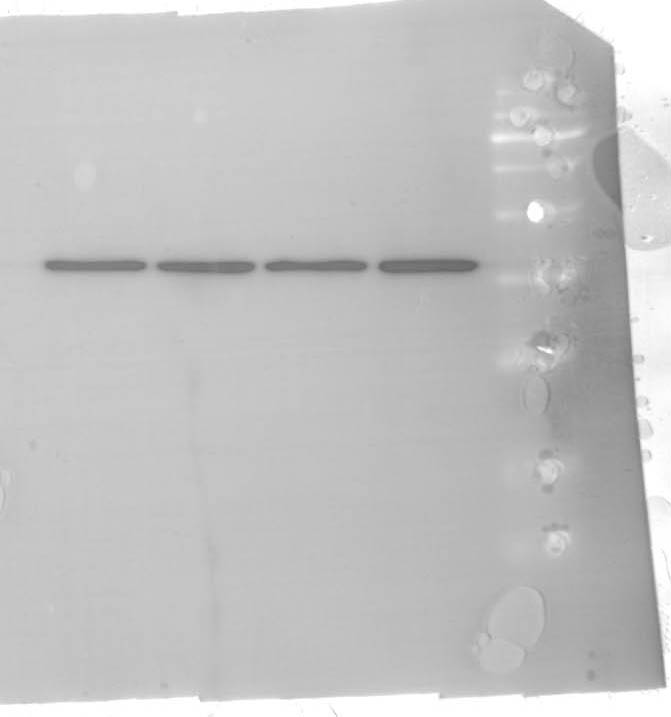

Supplement: Supplementary file 1 [file biomolecules-16-00689-s001.zip › File S1. original WB images/biomolecules-4275260_Original blots/Figure S18_pp38, p38, pHSP27, GAPDH/Figure S18B/GAPDH and markers overlay.jpg]

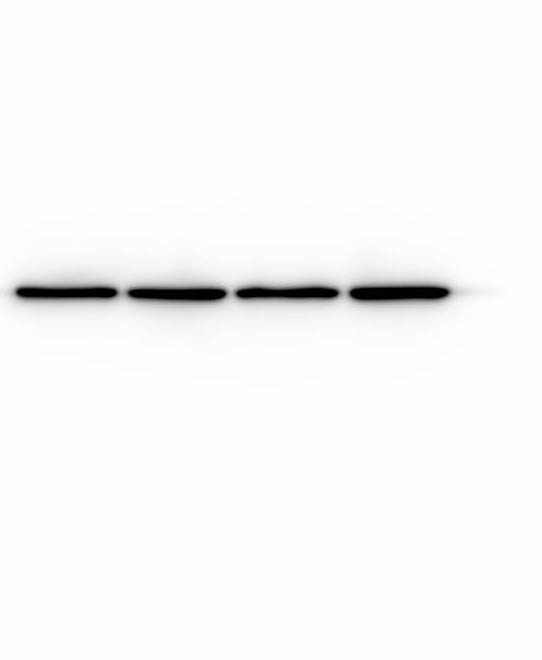

Supplement: Supplementary file 1 [file biomolecules-16-00689-s001.zip › File S1. original WB images/biomolecules-4275260_Original blots/Figure S18_pp38, p38, pHSP27, GAPDH/Figure S18B/GAPDH.jpg]

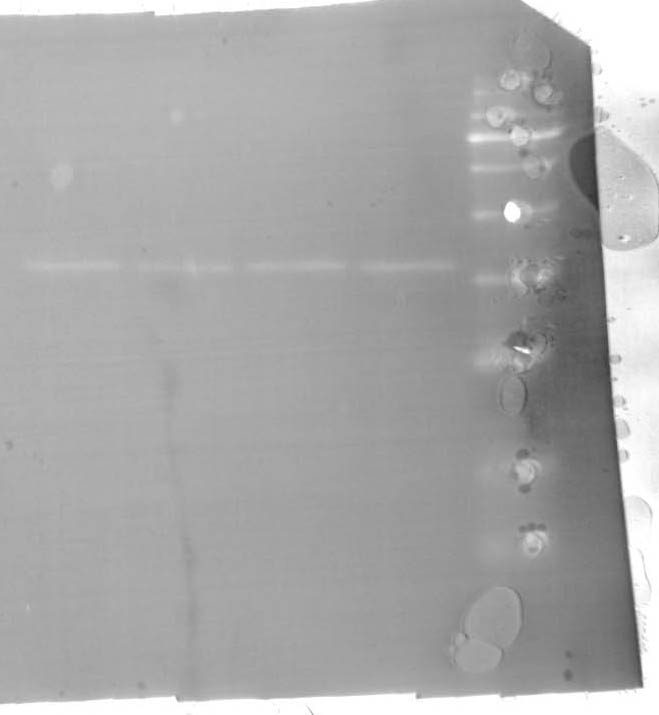

Supplement: Supplementary file 1 [file biomolecules-16-00689-s001.zip › File S1. original WB images/biomolecules-4275260_Original blots/Figure S18_pp38, p38, pHSP27, GAPDH/Figure S18B/markers GAPDH.jpg]

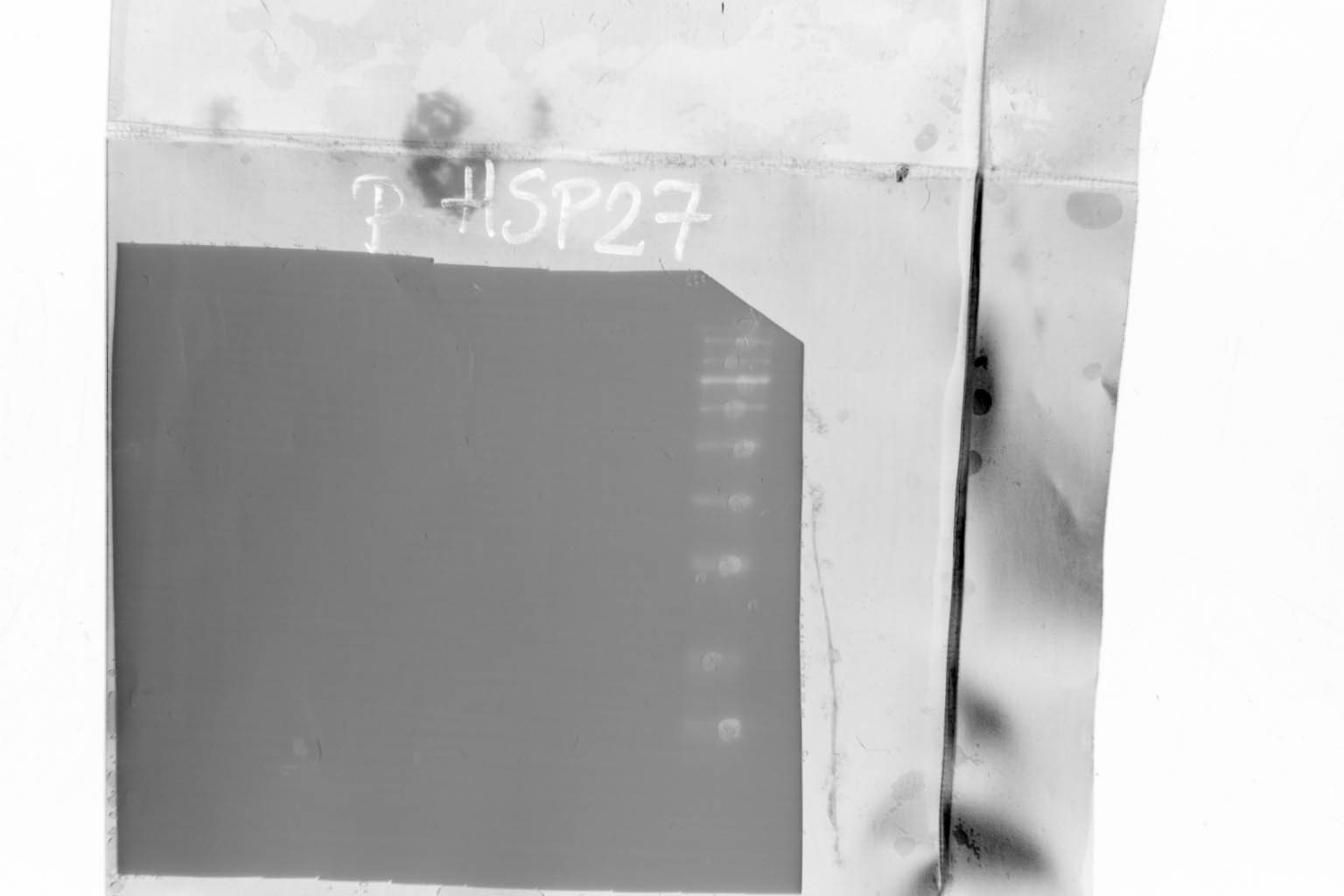

Supplement: Supplementary file 1 [file biomolecules-16-00689-s001.zip › File S1. original WB images/biomolecules-4275260_Original blots/Figure S18_pp38, p38, pHSP27, GAPDH/Figure S18B/markers pHSP27.jpg]

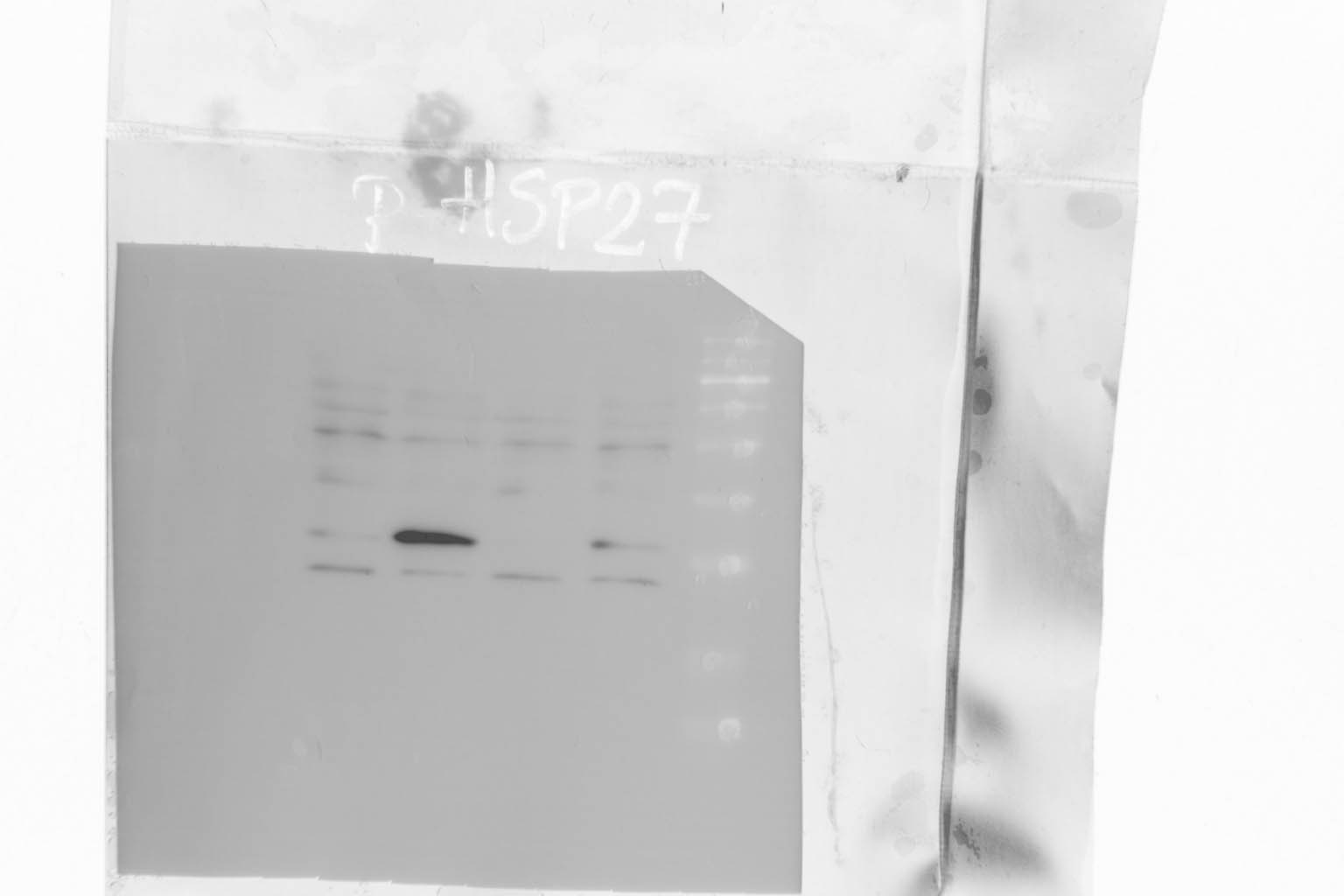

Supplement: Supplementary file 1 [file biomolecules-16-00689-s001.zip › File S1. original WB images/biomolecules-4275260_Original blots/Figure S18_pp38, p38, pHSP27, GAPDH/Figure S18B/pHSP27 and markers overlay.jpg]

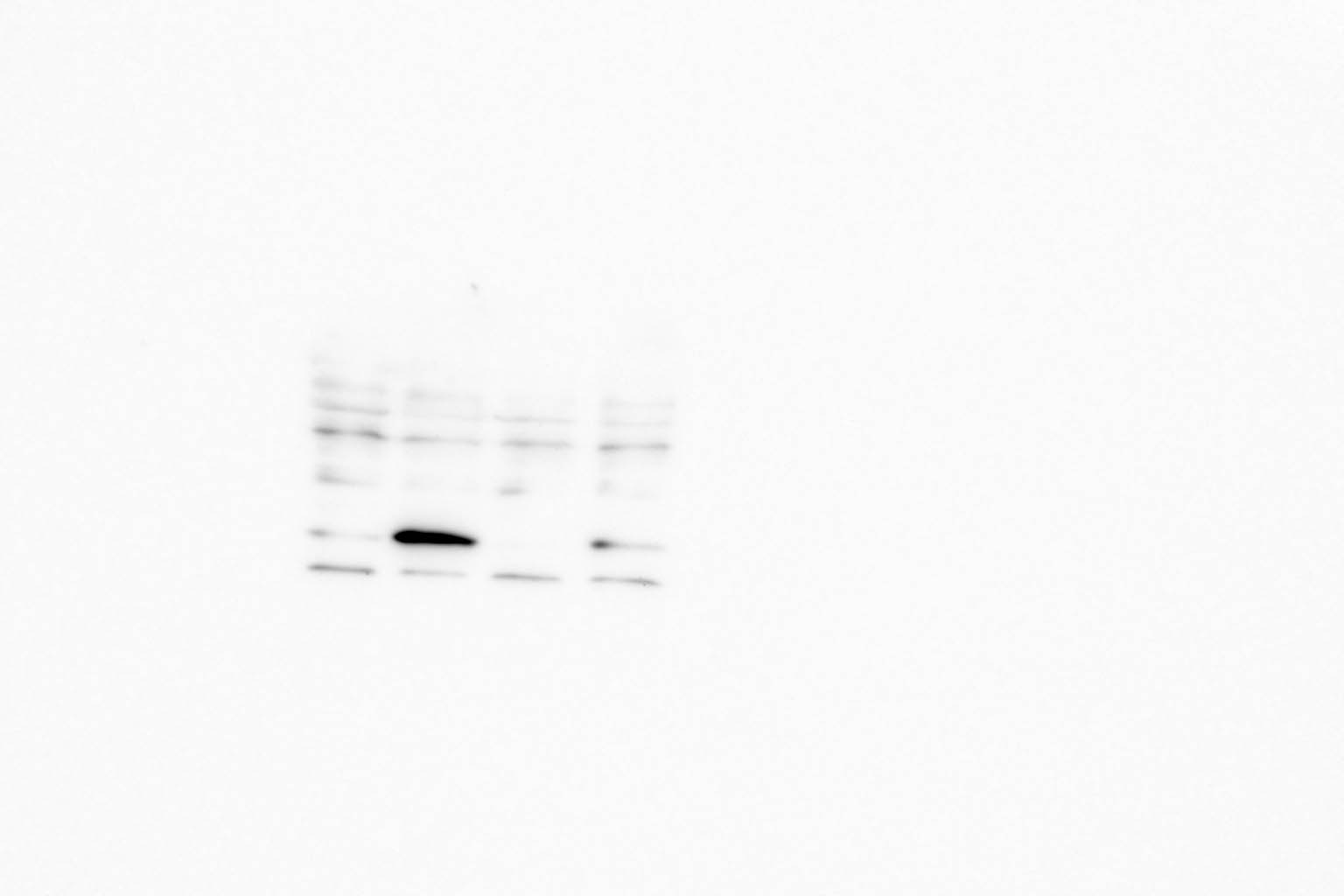

Supplement: Supplementary file 1 [file biomolecules-16-00689-s001.zip › File S1. original WB images/biomolecules-4275260_Original blots/Figure S18_pp38, p38, pHSP27, GAPDH/Figure S18B/pHSP27.jpg]
